# Aromatic‐Carbonyl Interactions as an Emerging Type of Non‐Covalent Interactions

**DOI:** 10.1002/advs.202310337

**Published:** 2024-04-01

**Authors:** Chaowei Yin, Hebo Ye, Yu Hai, Hanxun Zou, Lei You

**Affiliations:** ^1^ State Key Laboratory of Structural Chemistry Fujian Institute of Research on the Structure of Matter Chinese Academy of Sciences Fuzhou Fujian 350002 China; ^2^ University of Chinese Academy of Sciences Chinese Academy of Sciences Beijing 100049 China

**Keywords:** carbonyl, dynamic covalent chemistry, non‐covalent interactions, solvent effects ;supramolecular chemistry

## Abstract

Aromatic‐carbonyl (Ar···C═O) interactions, attractive interactions between the arene plane and the carbon atom of carbonyl, are in the infancy as one type of new supramolecular bonding forces. Here the study and functionalization of aromatic‐carbonyl interactions in solution is reported. A combination of aromatic‐carbonyl interactions and dynamic covalent chemistry provided a versatile avenue. The stabilizing role and mechanism of arene‐aldehyde/imine interactions are elucidated through crystal structures, NMR studies, and computational evidence. The movement of imine exchange equilibria further allowed the quantification of the interplay between arene‐aldehyde/imine interactions and dynamic imine chemistry, with solvent effects offering another handle and matching the electrostatic feature of the interactions. Moreover, arene‐aldehyde/imine interactions enabled the reversal of kinetic and thermodynamic selectivity and sorting of dynamic covalent libraries. To show the functional utility diverse modulation of fluorescence signals is realized with arene‐aldehyde/imine interactions. The results should find applications in many aspects, including molecular recognition, assemblies, catalysis, and intelligent materials.

## Graphical abstract

A convergence of aromatic‐carbonyl interactions and dynamic covalent chemistry allowed the examination and quantification of this class of under‐explored supramolecular bonding forces in solution, with underlying structural and mechanistic insights elucidated. The functional utility of arene‐aldehyde/imine interactions in controlling dynamic libraries and fluorescence signals is further demonstrated, enriching the collection of non‐covalent interactions.

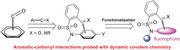

## 1 Introduction

**Figure 1:**
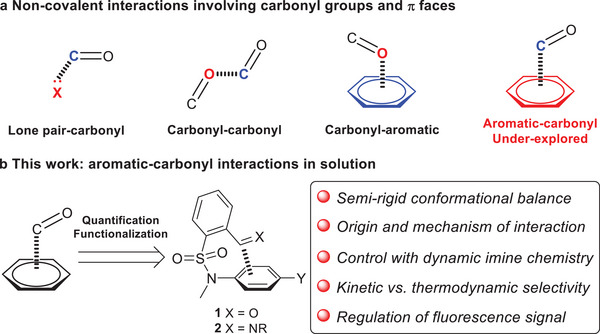
The illustration of non‐covalent interactions invoked by carbonyl groups and π faces a) and aromatic‐carbonyl interactions as well as the interplay with dynamic imine chemistry in the current work b).

Non‐covalent interactions of π faces play a notable role in chemistry and biology.^[^ ^1^ ^]^ Supramolecular bonding forces involving π faces of carbonyl groups are generating increasing attention, including lone pair‐carbonyl (n···C═O) and carbonyl‐carbonyl (C═O···C═O) interactions (**Figure** **1**a).^[^ ^2^ ^]^ Both electrostatics and n→π^*^ orbital delocalization make the contribution, with the strength distance‐dependent.^[^ ^3^ ^]^ These n···C═O and C═O···C═O interactions have been shown to control molecular conformations,^[^ ^4^ ^]^ stabilize peptide/protein structures,^[^ ^5^ ^]^ regulate reversible covalent systems,^[^ ^6^ ^]^ and accelerate molecular rotors.^[^ ^7^ ^]^ Carbonyl oxygen lone pair‐π aromatic (C═O···Ar) interactions (Figure 1a) also exist in crystal structures and were found to be electrostatically driven.^[^ ^8^ ^]^ The supramolecular interactions including carbonyl or aromatic faces in metal complexes were explored.^[^ ^9^ ^]^ Despite diverse non‐covalent interactions of carbonyl groups, aromatic‐carbonyl (Ar···C═O) interactions, with arene and carbonyl as the electron donor and acceptor, respectively, are scarce (Figure 1a). Recently the existence of arene‐carbonyl interaction was reported in an adduct from benzofuran and formaldehyde in the gas phase.^[^ ^10^ ^]^ The development of synthetic scaffolds exhibiting aromatic‐carbonyl interactions in the solid‐state and solution hasn't been reported, and their functionalization remains untouched.

By combining the dynamic nature of non‐covalent interactions and the strength of covalent bonds in the form of reversible covalent bonds, research on dynamic covalent chemistry (DCC) has been thriving,^[^ ^11^ ^]^ finding wide utility in the construction of functional assemblies and materials.^[^ ^12^ ^]^ The molecular diversity of dynamic covalent systems renders them a versatile avenue for probing and quantifying non‐covalent interactions.^[^ ^13^ ^]^ The goal of this work is the investigation of arene‐carbonyl interactions and their intercorrelation with DCC in solution. With tetrahedral tertiary sulfonamide serving as a semi‐rigid linker to impose restrained conformation we envisaged that the proximity of the arene plane and aldehyde (**1**) would offer an ideal platform for studying aromatic‐carbonyl interactions (Figure 1b). As one class of the most employed dynamic covalent reactions (DCRs),^[^ ^14^ ^]^ the formation and exchange of imines (**2**) would afford a barometer for examining arene‐aldehyde/imine interactions. Furthermore, the influence of Ar···C═X (X = O, NR) interactions on DCC systems would provide opportunities for functionalization.

In the current work, a series of *ortho*‐tertiary sulfonamide substituted aromatic aldehydes were developed for exploring aromatic‐carbonyl interactions. A collection of experimental and computational evidence revealed that arene···C═X (X = O, NR) interactions have a stabilizing role on aldehydes/imines, with electrostatic and dispersion components mainly accounting for the attractive forces. The effects of arene‐aldehyde/imine interactions on shift imine exchange equilibria were then elucidated and correlated, and solvent effects enabled further regulation. Moreover, by leveraging the difference between imines incorporating secondary and tertiary sulfonamides the reversal of kinetic and thermodynamic selectivity allowed the sorting of dynamic covalent libraries. Finally, the functional utility of aromatic‐carbonyl interactions was demonstrated with the modulation of fluorescence signals. The structural and mechanistic insights revealed enrich the toolbox of non‐covalent interactions and pave the way for future endeavors.

## 2 Results and Discussion

### 2.1 Synthesis and Structures

**Figure 2:**
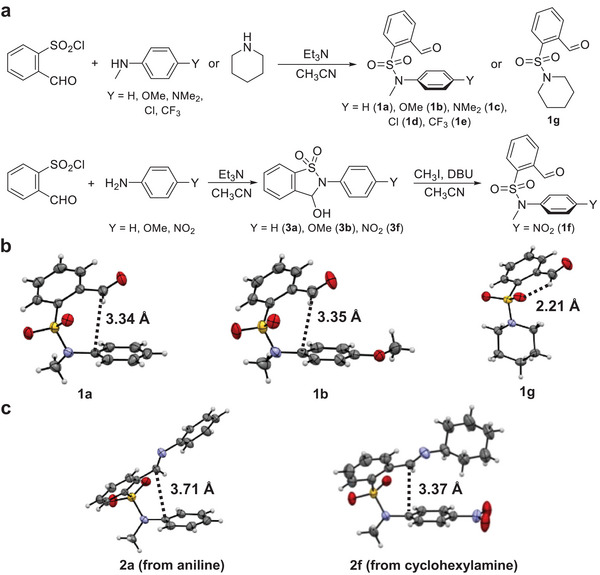
a) Synthesis of 2‐formylbenzenesulfonamide compounds. b) Crystal structures of aldehydes **1a**, **1b**, and **1** **g**. c) Crystal structures of imines **2a** (from aniline) and **2f** (from cyclohexylamine). The C···C or CH···O distances are listed.

With the strategy in place, 2‐formylbenzenesulfonamide derivatives (**1**) were synthesized by the reactions of 2‐formylbenzenesulfonyl chloride and corresponding amines (**Figure** **2**a). In an alternative method, the methylation of secondary sulfonamides (**3**) with base present afforded their tertiary counterparts (**1**). In addition to aromatic sulfonamides bearing varying substituents (**1a**‐**1f**) an aliphatic analog (**1** **g**) was employed as a control. To contrast dynamic covalent reactivity with tertiary sulfonamides (**1**) and associated imines (**2**), representative secondary sulfonamides (**3**) and their imines (**4**) were also studied.

X‐ray crystal analysis offered valuable structural insights, with **1a**‐**1f** sharing common geometric features (Figure 2b; Figure S29, Supporting Information). Notably, the formyl group is placed above the sulfonamide arene plane, engaging in potential arene‐aldehyde interaction. The C···C distances of 3.34 (**1a**) and 3.35 (**1b**) Å fall within the sum of van der Waals radius and are thus indicative of attractive interactions. The formyl group rotates in order to interact with an aromatic plane on sulfonamide nitrogen. Other substituents gave similar results (Figure S29, Supporting Information). With the conjugation between nitrogen and attached arene sabotaged, the varying substituent (Y) allows the modulation of aromatic‐carbonyl interactions. Moreover, the hydrogen bonding between formyl CH and sulfonamide oxygen was found in **1a**‐**1f** as well as **1** **g** (CH···O 2.21 Å). This is important as the maintenance of CH···O hydrogen bonds throughout **1a**‐**1g** would largely offset their interference. The interaction between arene plane and imine bond was also found in crystal structures of aniline‐derived imine **2a** (C···C 3.71 Å) as well as cyclohexylamine‐derived imine **2f** (C···C 3.37 Å) (Figure 2c).

There is a significant difference in ^1^H NMR chemical shift of CHO for **1a** (9.81 ppm), **1b** (9.87 ppm), **1c** (9.78 ppm), **1d** (10.03 ppm), **1e** (10.11 ppm), **1f** (10.18 ppm), and **1** **g** (10.81 ppm) in CDCl_3_ solution. Moreover, a good linear correlation was obtained between the chemical shift value of the formyl proton of **1a**‐**1f** and Hammett parameter σ_m_ (R^2^ = 0.961, Figures S30 and S31, Supporting Information). Such a trend further supports the existence of through‐space arene‐formyl interaction: while phenyl (**1a**) and *p*‐dimethylaminophenyl (**1c**) could decrease the electrophilicity of the aldehyde, the electron‐withdrawing capability of *p*‐nitro group (**1f**) would attenuate such an effect. Without arene‐formyl interaction, **1** **g** had the largest chemical shift value.

### 2.2 DFT Calculations

**Figure 3:**
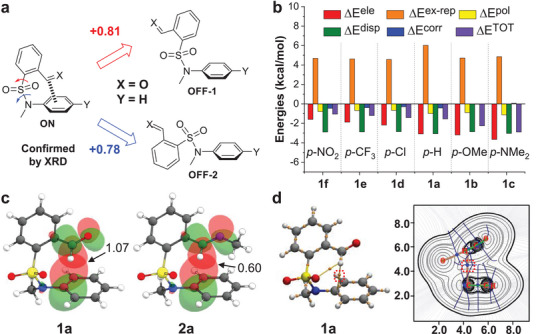
Mechanistic insights of arene‐aldehyde/imine interactions in computational studies. a) Conformational isomers with or without the interaction, with the relative free energies (kcal mol^−1^) of rotamers of **1a** shown. b) Total and dissected interaction energies for ON conformer of aldehyde **1** in GKS‐EDA analysis. c) NBO orbitals and stabilization energies (kcal mol^−1^) of π→π^*^ interactions for **1a** and **2a**. d) Topological analysis of the electron density distribution for **1a**, with bond critical points (BCP) of (3, −1) and Laplacian of electron density of arene‐aldehyde interaction (with red dot frame) shown.

To further gauge the feasibility of arene‐aldehyde/imine interactions and unravel underlying mechanistic foundation density functional theory (DFT) calculations were conducted. The function of B3LYP‐D3 and the basis set of def2‐TZVP were employed, with a polarized continuum model (PCM) for acetonitrile. Gratifyingly, conformational analysis of **1a** revealed that the most stable rotamer (ON) falls in line with the crystal structure (**Figure** **3**a; Figure S32, Supporting Information). Furthermore, two more conformers by virtue of the rotation about S‐C and S‐N bonds were found, affording relative energies of 0.81 (OFF‐1) and 0.78 (OFF‐2) kcal mol^−1^, respectively (Figure 3a). The thermodynamic stabilization of the ON conformer was further validated with aldehydes (**1a**‐**1f**) and their imines from methyl amine (**2a**‐**2f**) (Table S3, Supporting Information). Methyl amine‐derived imines were chosen as simplified models to limit potential interfering contacts between amine substituent and the arene plane. The favoring of the isomer adopting aromatic‐carbonyl interaction (ON) over those without it (OFF) echoes structurally rigid conformational balances employed for measuring non‐covalent interactions.^[^ ^15^ ^]^

Generalized Kohn‐Sham energy decomposition analysis (GKS‐EDA) ^[^ ^16^ ^]^ was then performed to calculate the total interaction energy (ΔE^TOT^) of intramolecular arene‐aldehyde/imine interactions in ON conformation of **1**/**2** and dissect the contributing components, such as electrostatic (ΔE^ele^), polarization (ΔE^pol^), and dispersion (ΔE^disp^) (Table S4, Supporting Information). The values of ΔE^TOT^ of aldehyde **1** are sensitive to substituent change, with a decreasing sequence (i.e, more negative) from electron‐withdrawing NO_2_ (**1f**) to electron‐donating Me_2_N (**1c**) (Figure 3b). Electrostatic (ΔE^ele^) and dispersion (ΔE^disp^) terms make major contributions to ΔE^TOT^, with relatively small polarization (ΔE^pol^) term. Furthermore, the substituent‐dependent change in ΔE^TOT^ is largely due to the variation of electrostatic, with ΔE^ele^ overtaking ΔE^disp^ for **1b** and **1c**. The domination of electrostatic and dispersion components was also revealed for **2**, though ΔE^disp^ surpasses ΔE^ele^ for all substituents studied. While minor, the contribution of polarization (ΔE^pol^) is more significant for aldehyde **1** than imine **2** (less than 10%). These findings match the enhanced dipolar nature of the aldehydes over their imines.

Despite the small percentage of orbital delocalization (i.e., polarization term ΔE^pol^) in GKS‐EDA analysis, natural bond orbital (NBO) analysis allowed the identification of participating orbitals (Table S7, Supporting Information).^[^ ^17^ ^]^ Taking **1a** as an example, the alignment of π orbital along C1‐C2 of the phenyl ring and π^*^ orbital of carbonyl leads to the partial overlap (Figure 3c). The π→π^*^ orbital interaction energy was found to be 1.07 kcal mol^−1^ according to the second‐order perturbation theory (ΔE^(2)^). Due to the distance‐ and orientation‐dependent feature of orbital interactions, the aldehyde group rotates out of the attached phenyl plane to accommodate π→π^*^ interaction (Table S8, Supporting Information). The π→π^*^ interaction was also found for imine **2a**, affording an NBO energy of 0.60 kcal mol^−1^. These results confirm through‐space π→π^*^ interactions within aldehydes/imines. The existence of bond critical points (BCP) (3, −1) in the topological analysis of the electron density distribution ^[^ ^18^ ^]^ further verified arene‐aldehyde/imine interactions (Figure 3d; Figure S35 and Table S9, Supporting Information), as also non‐covalent interaction (NCI) analysis (Figure S36, Supporting Information).^[^ ^19^ ^]^ The location of BCP between C1 and aldehyde/imine carbon supports attractive Ar···C═X (X = O, NR) interactions.

### 2.3 Correlation with Imine Exchange

We next set to perform DCRs of imine formation (Figures S37–S54, Supporting Information). The reaction of **1a** and 1‐butylamine proceeded pretty slowly, creating **2a** quantitatively after 6 days without molecular sieves (Figures S37–S39, Supporting Information). The sluggish imine formation was also attained with **1b** and **1f** to give **2b** and **2f**, respectively (Figures S40–S42 and S49–S51, Supporting Information). One rationalization likely comes from the kinetic deactivation of the aldehyde arising from arene‐formyl interaction. In this regard, such a “protecting” effect is reminiscent of the role of n→π^*^ interaction in decelerating imine formation.^[6a]^

**Figure 4:**
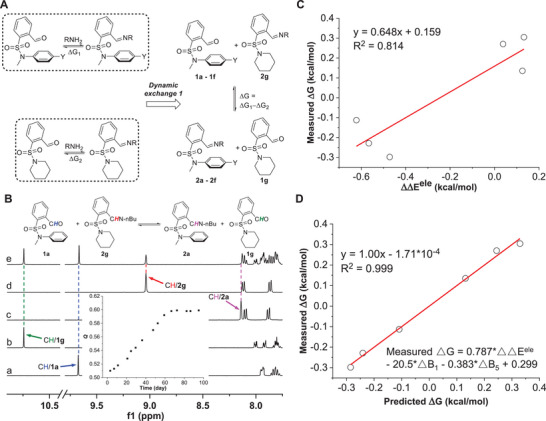
A) Quantification of arene‐aldehyde/imine interactions with imine exchange equilibria. B) ^1^H NMR spectra of **1a** (a), **1** **g** (b), **2a** (c), **2** **g** (d), and imine exchange (e) in CD_3_CN, with the kinetic profile shown in the inset. *Q* is the reaction quotient. C) Plot of experimental ΔG values of imine exchange 1 versus ΔΔE^ele^. D) Multivariate linear correlation of experimental ΔG values of imine exchange 1 versus ΔΔE^ele^, ΔB_1_, and ΔB_5_ values. ΔΔE^ele^, ΔL, and ΔB_1_ are the differences between ΔE^ele^, B_1_, and B_5_ values of aldehyde **1** and imine **2**. For the measurement of the parameters’ significance, the standardized coefficient was found to be 1.10, −0.467, and −0.230 for ΔΔE^ele^, ΔB_1_, and ΔB_5_, respectively.

Attention was then turned to the thermodynamic effect of arene‐carbonyl interaction on imine DCC. Instead of measuring individual imine creation reactions, dynamic aldehyde exchange reactions were set up as a facile methodology for the quantification. The free energy change (ΔG) of imine exchange 1, which is the difference between ΔG of imine formation equilibria, would be reflective of the impact of weak interactions on imine chemistry (**Figure** **4**A). For example, a mixture of **1a**, **1** **g**, and 1‐butylamine was subjected to equilibration, and the component distribution was tracked. The equilibrium was reached after 66 days, affording an equilibrium constant (*K*) of 0.598 and in favor of **2** **g** (Figure 4B; Figures S55–S56, Supporting Information).

The preference of imine **2** **g** was maintained for analogous aldehyde exchange reaction of **1b** (*K* = 0.634) and **1c** (*K* = 0.796) (Figures S57–S60, Supporting Information). Differently, a *K* value of 1.47, 1.66, and 1.21 was revealed for **1d**, **1e**, and **1f**, slightly favoring imines **2d**, **2e**, and **2f**, respectively (Table S10; Figures S61–S66, Supporting Information). To probe whether the change in equilibrium constants of imine exchange reactions is caused by arene‐aldehyde/imine interactions, experimental results were correlated with theoretical data. A linear line was not obtained when ΔG values of imine exchange 1 were plotted against the difference between ΔE^ele^ values of aldehyde **1** and imine **2** (ΔΔE^ele^) (Figure 4C). Considering arene‐aldehyde/imine interactions would be influenced by the bulkiness and orientation of interacting functional groups, multi‐dimensional sterimol parameters of aldehyde/imine fragments were calculated (Table S5, Supporting Information).^[^ ^20^ ^]^ Gratifyingly, multivariate analysis afforded a linear correlation of experimental ΔG values versus ΔΔE^ele^, ΔB_1_, and ΔB_5_ values (Figure 4D). B_1_ and B_5_ represent the shortest and longest distance perpendicular to the primary axis of attachment and reflect the steric interaction imposed by the aldehyde/imine group. The quality of correlation was further confirmed with an excellent linear relationship between measured and predicted ΔG values (slope = 1.00, R^2^ = 0.999) (Figure 4D). Moreover, by using a standardization method of regression coefficient the major contribution of ΔΔE^ele^ on multivariate correlation was validated (Table S6, Supporting Information). These results reinforce the electrostatic feature of arene‐aldehyde/imine interactions and their critical role in dictating imine DCC.

### 2.4 Solvent Effects

**Figure 5:**
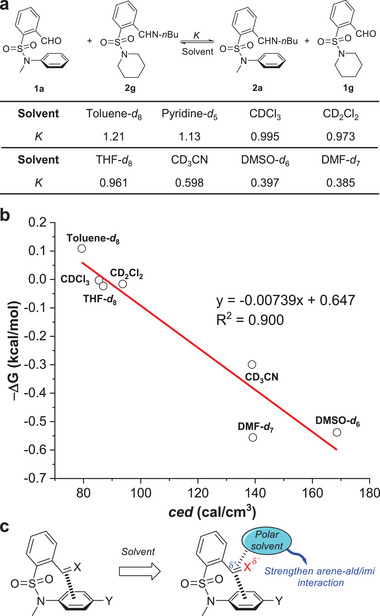
a) The summary of *K* values of imine exchange 1 in different solvents. b) Correlation of ‐Δ*G* of imine exchange in panel a with the cohesive energy density (*ced*) of the solvent. c) Proposed arene‐aldehyde/imine interactions in polar aprotic solvents (X = O, NR).

The impact of solvent on non‐covalent interactions and molecular recognition is an essential and longstanding topic in supramolecular chemistry.^[^ ^21^ ^]^ Toward this end, the aldehyde exchange reactions were run in different solvents, including toluene, pyridine, chloroform, dichloromethane, tetrahydrofuran (THF), acetonitrile, dimethylformamide (DMF), and dimethyl sulfoxide (DMSO). The change of solvent had a significant thermodynamic effect on imine exchange (**Figure** **5**a; Figures S67–S74, Supporting Information). Non‐polar solvent toluene gave the largest *K* value of 1.21, while polar aprotic solvents DMF and DMSO afforded the smallest *K* value (0.385 and 0.397). The data in other solvents fell in the middle. One consideration comes from the competition from solvent‐arene interactions, such as those in the form of π stacking, CH/π, and polar/π interactions for toluene, chloroform, and THF, respectively. Since those interfering interactions involving arene planes are possible for both aldehyde (**1a**) and its imine (**2a**) on two sides of exchange 1, the effects could be offset. It is notable that toluene and pyridine had similar *K* values (1.21 and 1.13). This is reasonable since π electron density is responsible for the stacking interactions.

The influence of solvents was then quantified by correlation with empirical parameters. The ‐Δ*G* values of imine exchange reactions in different solvents were plotted against the cohesive energy density (*ced*) of the solvent, which is reflective of intermolecular interactions between solvent molecules. The data in pyridine was not included due to the additional factors as described above. A linear trendline was obtained, with a negative slope (R^2^ = 0.900) (Figure 5b; Table S11, Supporting Information). There is a decrease in ‐Δ*G* (smaller *K* value) as *ced* parameter increases for the aprotic solvent tested, as the case for DMF and DMSO. On the other end, toluene has the lowest *ced* value (79.4) and afforded the biggest ‐Δ*G* value (largest *K*). The bond polarization of the formyl group (C═O) in more polar aprotic solvents could result in a stronger arene‐aldehyde interaction and hence a decrease in the equilibrium constant (Figure 5c). As reflected in natural population analysis (NPA) charge on aldehyde carbon in calculated **1a**, an increase in more polar solvent is apparent (Table S12, Supporting Information). A similar trend was found for imine **2a**, though the C═N bond polarization is modest in relative to aldehyde **1a**. Tying it all together, solvent effects offered mechanistic insights on arene‐aldehyde/imine interactions and afforded a facile handle for manipulating imine DCC.

### 2.5 Kinetic Versus Thermodynamic Selectivity

**Figure 6:**
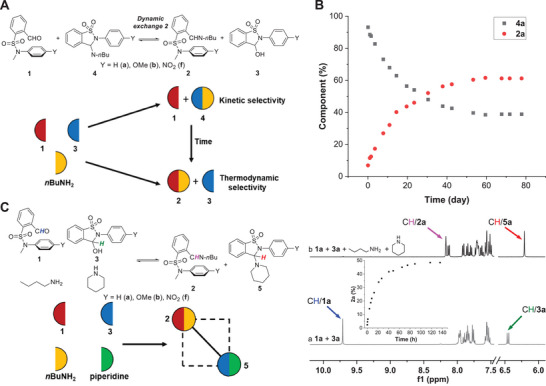
A) Illustration of control of kinetic and thermodynamic selectivity for the reactions of **1**, **3**, and 1‐butylamine with imine exchange 2. B) Kinetic profile for a mixture of **1a**, **3a** (1.0 equiv.), and 1‐butylamine (1.0 equiv.) to monitor the formation of imines **2a** and **4a** in CD_3_CN. C) Self‐sorting for a dynamic library of **1**, **3** (1.0 equiv.), 1‐butylamine (1.0 equiv.), and piperidine (1.0 equiv.) in CD_3_CN, with ^1^H NMR spectra of the initial mixture of **1a** and **3a** (a) and after reaching equilibrium with amines (b) shown. The kinetic profile is in the inset.

Having proved arene‐aldehyde/imine interactions and their intercorrelation with DCC the competition between secondary (**3**) and tertiary (**1**) sulfonamides for DCRs with amines was attempted (imine exchange 2, **Figure** **6**A). Secondary sulfonamide‐derived aldehydes (**3**) show high reactivity toward amines via internal catalysis imparted by the adjacent acidic sulfonamide, since open aldehyde and its cyclic hemiaminal are in equilibrium with base (i.e., amine) present.^[^ ^22^ ^]^ The competition between **1** and **3** for DCRs with primary amines was probed by imine exchange 2 (Figure 6A). The mixture of **1a**, **3a**, and 1‐butylamine was monitored, with imine **4a** preferred at an early stage and imine **2a** preferred upon equilibration after 50 days (*K* = 1.73) (Figure 6B; Figures S75 and S76, Supporting Information). Moreover, **1b** and **1f** also gave slow kinetics in analogous aldehyde exchange, and tertiary sulfonamide‐derived imines **2b** (*K* = 1.94) and **2c** (*K* = 2.74) were preferred after reaching equilibrium (Table S13; Figures S77–S80, Supporting Information). Therefore, the imines derived from secondary and tertiary sulfonamides were preferred kinetically and thermodynamically, respectively. In essence, the reversal of kinetic and thermodynamic selectivity of dynamic covalent libraries was achieved.

To further enhance the discrimination competitive selection toward self‐sorting within dynamic covalent libraries was explored (Figure 6C).^[^ ^23^ ^]^ The varying dynamic covalent reactivity of **1** and **3** toward secondary amines was utilized to amplify their preference for primary amines. A mixture of **1a**, **3a**, 1‐butylamine, and piperidine in equimolar amounts was tracked, and the equilibrium was readily reached after 4 days, notoriously faster than the competition without piperidine. The catalytic effect of the secondary amine could contribute to the strong acceleration. Furthermore, only the imine (**2a**) incorporating **1a** and 1‐butylamine and the cyclic aminal (**5a**) from **3a** and piperidine were observed (Figure 6C; Figures S81 and S82, Supporting Information). In essence, the selectivity for the primary amine between tertiary and secondary sulfonamides was enhanced, leading to the simplification and reactional self‐sorting within the [2 × 2] library. The kinetic acceleration as well as selectivity enhancement was also corroborated with an exchanging network from **1b**/**3b** or **1f**/**3f** (Figures S83–S86, Supporting Information).

### 2.6 Utility of Aromatic‐Carbonyl Interactions

**Figure 7:**
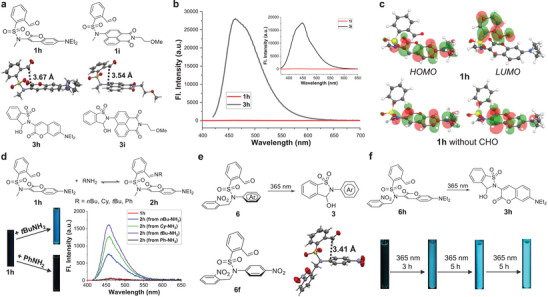
Regulation of fluorescence with arene‐aldehyde/imine interactions. a) Aromatic fluorophore containing tertiary (**1**) and secondary (**3**) sulfonamides, with crystal structure of **1** shown. b) The comparison of the fluorescence of **1** **h** and **3** **h**, with the data of **1i** and **3i** in the inset. c) HOMO and LUMO orbitals of **1** **h** and control compound without the formyl group. d) The comparison of the fluorescence of **1** **h** and imine **2** **h** with varied bulkiness and conjugation. e) Design of photoactivatable tertiary sulfonamides **6**, with crystal structure of **6f** shown. f) Photoinduced fluorescence upon irradiation of **6** **h** in CD_3_CN at 365 nm.

By taking advantage of the tunable platform for controlling arene‐aldehyde/imine interactions and imine DCC the functional utility was pursued. Toward this end, the emission of sulfonamide‐modified luminogens was regulated. In our previous work, a series of 2‐formylbenzenesulfonamide attached fluorophores allowed the realization of diverse signaling patterns and multistate switching ^[22b]^ Two fluorophores, namely 3‐amino‐7‐diethylaminocoumarin and 4‐amino‐1,8‐naphthalimide, were chosen, and their secondary (**3** **h** and **3i**) and tertiary (**1** **h** and **1i**) sulfonamides were prepared (**Figure** **7**a). The crystal structures of **1** **h** and **1i** validated arene‐formyl interactions, as the aldehyde group resides above the fluorophore plane (C···C 3.67 and 3.54 Å). While **3** **h** and **3i** afforded strong blue emission at 463 and 450 nm, respectively, **1** **h** and **1i** were nearly non‐fluorescent (Figure 7b; Figures S87 and S88, Supporting Information). This is likely due to quenching induced by arene‐formyl interactions. Moreover, DFT calculations supported the interpretation, as shown by the location of HOMO and LUMO orbitals of **1** **h** mainly on the coumarin plane and the arylaldehyde unit, respectively (Figure 7c; Figures S89 and S90; Tables S14 and S15, Supporting Information).

When DCRs of **1** **h** with primary amines were conducted, the fluorescence was turned on for aliphatic amine‐derived imines (**2** **h**), including 1‐butylamine, cyclohexylamine, and *t*‐butylamine (Figure 7d; Figures S91 and S92, Supporting Information). Moreover, there was an increase in the emission with enhanced steric hindrance. Differently, the emission remained suppressed for aniline‐derived imine **2** **h** (Figure 7d). These results are reasonable as the rise of bulkiness would attenuate arene‐imine interaction, while an opposite effect would be invoked by the extended conjugation. A photoresponsive aldehyde was further designed for the control of emission. Specifically, the photoremovable protecting group 2‐nitrobenzyl ^[^ ^24^ ^]^ was incorporated into tertiary sulfonamide‐derived aldehyde (**6**, Figure 7e; Figure S93, Supporting Information). The existence of arene‐aldehyde interaction was confirmed by the crystal structure of a model compound (**6f**). Upon the irradiation of a solution of **6** **h** in CD_3_CN for 3 h at 365 nm the fluorescence was toggled on, since the cleavage of 2‐nitrobenzyl unit by UV light led to the generation of **3** **h** (Figure 7f; Figure S94, Supporting Information). The prolonged illumination further activated the emission. NMR analysis confirmed the appearance of **3** **h** at the expense of **6** **h**. Therefore, the manipulation of non‐covalent interactions allows a facile approach for photoinduced fluorescence.

## 3 Conclusion

In summary, we developed a versatile platform for probing aromatic‐carbonyl (Ar···C═O) interactions and their interrelation with dynamic imine chemistry. A series of *ortho*‐tertiary sulfonamide restrained aromatic aldehydes were prepared in a modular way, and the stabilizing effects of arene‐aldehyde/imine interactions were elucidated through combined experimental and computational results. The electrostatic and dispersion components primarily make up the attractive forces, with the contribution of π→π^*^ orbital interaction minor. The thermodynamic impact of arene‐aldehyde/imine interactions on shift imine exchange equilibria was further quantified and regulated with solvent effects, falling in line with the electrostatic feature of the interactions. In addition, the competition between imines incorporating secondary and tertiary sulfonamides enabled the reversal of kinetic and thermodynamic selectivity and the sorting of dynamic covalent libraries. Finally, the utility of arene‐aldehyde/imine interactions was showcased with the diverse modulation of fluorescence signals. The cooperativity of different non‐covalent interactions within molecular assemblies will be pursued in the future to amplify the effect of arene‐aldehyde/imine interactions. The results here add aromatic‐carbonyl interactions into the collection of non‐covalent bonding forces and would lay the foundation for future endeavors of molecular recognition, dynamic assemblies, and smart materials.

Deposition numbers 2312606–2312614 (for **1a‐1i**), 2 312 615 (for **2a**), 2 312 616 (for **2f**), and 2 312 617 (for **6f**) contain the supplementary crystallography data for this paper. These data are provided free of charge by the joint Cambridge Crystallographic Data Centre and Fachinformationszentrum Karlsruhe Access Structures service.

## Conflict of Interest

The authors declare no conflict of interest.

## Supporting information

Supporting Information

Supporting Information

## Data Availability

The data that support the findings of this study are available in the supplementary material of this article.
