# Supplementary material for: Aromatic‐Carbonyl Interactions as an Emerging Type of Non‐Covalent Interactions
Source: Adv Sci (Weinh). 2024 Apr 1;11(22):2310337. doi: 10.1002/advs.202310337 (PMC11165483; doi:10.1002/advs.202310337)
Supplement: Supplementary file 1 — Supporting Information [file ADVS-11-2310337-s001.pdf]

ADVANCED SCIENCE
Open Access

Supporting Information

for *Adv. Sci.*, DOI 10.1002/advs.202310337

Aromatic-Carbonyl Interactions as an Emerging Type of Non-Covalent Interactions

*Chaowei Yin, Hebo Ye, Yu Hai, Hanxun Zou and Lei You**

Supporting Information

Aromatic-Carbonyl Interactions as an Emerging Type of Non-Covalent Interactions

Chaowei Yin,[a],[b],+ Hebo Ye,[a],+ Yu Hai,[a]

Hanxun Zou,[a] and Lei You [a],[b],*

[a] State Key Laboratory of Structural Chemistry, Fujian Institute of Research on the Structure of Matter, Chinese Academy of Sciences, Fuzhou 350002, China.

[b] University of Chinese Academy of Sciences, Beijing 100049, China.

TABLE OF CONTENTS

1. General Methods……………………..……………….…………S3-S4

2. Synthesis and Characterization………………..……….……..S5-S32

3. DFT Calculations………………………….……………..…....S33-S38

4. Dynamic Covalent Reactions

(1) Imine formation…………………………………...S39-S47

(2) Imine exchange……………………………………S48-S54

5. Solvent Effect…………………………………………….…S55-S59

6. Control over Thermodynamic and Kinetic Selectivity……..S60-S66

7. Regulation of Fluorescence………………………………S67-S74

8. Molecular Coordinates……………………………………….S75-S95

9. References……………………………………………………..S96

1. General Methods

^{1}H NMR and ^{13}C NMR spectra were recorded on a 400 MHz Bruker Biospin avance III spectrometer. Deuterated reagents for characterization and *in situ* reactions were purchased from Sigma-Aldrich Chemical Co. and Cambridge Isotope Laboratories, Inc. (purity ≥ 99.9%). The chemical shifts (δ) for ^{1}H NMR spectra, given in ppm, are referenced to the residual proton signal of the deuterated solvent. Mass spectra were recorded on a Bruker IMPACT–II spectrometer. Crystallographic data was collected on a Mercury single crystal diffractometer at room temperature. The structures were solved with direct methods by using OlexSys or SHELXS-97 and refined with the full-matrix least-squares technique based on F2 by using the OlexSys or SHELXL-97. All other reagents were obtained from commercial sources and were used without further purification, unless indicated otherwise.

Dynamic covalent reactions. Dynamic Covalent Reactions (DCRs) were performed in *situ* in CD_3CN at room temperature without isolation and purification. For imine formation reactions, to a solution of **1** (12 mM, 1.0 equiv.) in CD_3CN (0.60 mL) was added 1-butylamine (1.2 equiv.). The mixture was characterized by ^{1}H NMR and ESI mass spectral analysis. For competition experiments, both aldehydes (12 mM each, 1.0 equiv.) were mixed with 1-butylamine (1.0 equiv.) in different solvents (0.60 mL). The mixture was tracked by ^{1}H NMR until the equilibrium was reached. All competition experiments were performed twice to ensure reproducibility. For self-sorting experiments, both aldehydes (12 mM each, 1.0 equiv.) were mixed with 1-butylamine (1.0 equiv.) and piperidine (1.0 equiv.) in CD_3CN (0.60 mL). The mixture was tracked by ^{1}H NMR until the equilibrium was reached. See specific conditions in figure captions of the main text or supporting information if necessary.

Fluorescence experiments in solution. Fluorescence spectra in solution were recorded on a microplate reader (BioTek SYNERGY H4) at a concentration of 50 μM of each probe in acetonitrile. Stock solutions of components were prepared. Aldehyde and amine components were mixed in a similar way as NMR studies to create imines, and fluorescence spectra of imines were recorded after dilution. For NMR and fluorescence

analysis, please see specific conditions in figure captions of the main text or supporting information if necessary.

DFT Calculations. Geometry optimization and frequency calculations were performed by using Gaussian 09 (G09) packages,[S1] with the DFT method and basis set of B3LYP-D3/def2-TZVP. An ultrafine integration grid was also employed during the optimization and frequency analysis. Geometries were determined without imaginary frequencies. Through the conformational search of **1a**, three major conformers (ON, OFF-1, and OFF-2) were found with the percentage population over 5%, which were selected for the calculation of the conformers of aldehyde **1** and imine **2**. For imine **2** methyl amine derived imines were chosen as simplified models to limit potential interfering contacts between amine substituent and the aromatic plane. Generalized Kohn-Sham energy decomposition analysis (GKS-EDA) was employed to calculate the total interaction energy in ON conformation of **1**/**2** and dissect the contributing factors to aromatic-aldehyde/imine interactions by the modified GAMESS (version: 2020-R2) from XMVB team.[S2] Firstly, three fragments of ON conformers from the previous optimization were specified: the intramolecular interacting fragment 1 (a_1: C=X group), intramolecular interacting fragment 2 (a_2: *para*-substituted phenyl group), and the environmental fragment (e: the atoms except from a_1 and a_2 fragments). With the same DFT method and the basis set, three combinations of fragments including {a_1, e}, {a_2, e}, and {a_1, a_2, e} were employed in the GKS-EDA calculation, respectively. The intramolecular interaction free energy was finally obtained from the results of the total fragment interaction {a_1, a_2, e} subtracting that of {a_1, e} and {a_2, e}. The NBO analysis[S3] was implemented by NBO 3.1 module in G09. The electronic density cubes for Atoms in Molecules (AIM)[S4] and Non-covalent Interaction (NCI)[S5] analysis were generated by Multiwfn 3.80[S6] and presented by VMD 1.90.[S7] For UV and fluorescence property of **1h**, **2h**, and the control without formyl group, TD-DFT calculations were conducted with the same method and basis set. See more details in associated Figures and Tables if necessary.

2. Synthesis and Characterization

Scheme S1. General synthetic routes of **1** and **3**.

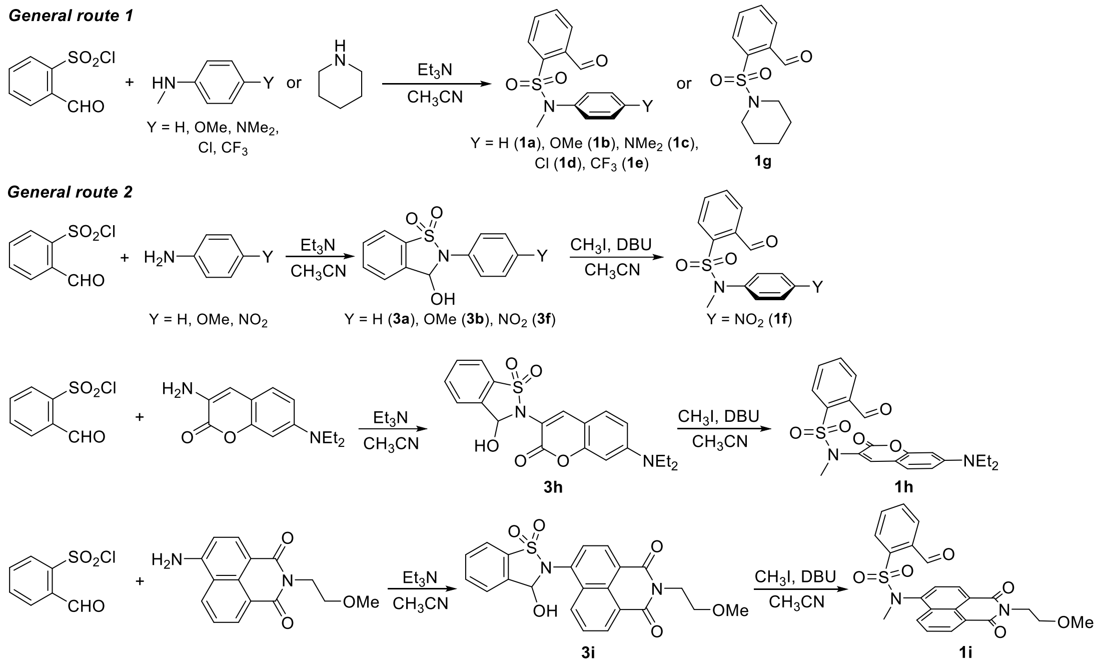

***General route 1*:** To a solution of one secondary amine (1.0 equiv.) in anhydrous acetonitrile was added triethylamine (1.0 equiv.). The mixed solution was then added to a stirring solution of 2-formylbenzenesulfonyl chloride (prepared according to the literature procedure,[S8] 1.5 equiv.) in acetonitrile dropwise. The mixture was stirred at room temperature for 5 hours and extracted with ethyl acetate. The combined organic layers were washed with brine and dried over Na_2SO_4, and the solvents were evaporated *in vacuo*. The residue was purified by silica gel column chromatography to afford compounds **1a**-**1e** and **1g**.

***General route 2*:** To a solution of 2-formylbenzenesulfonamide **3** (prepared according to the literature procedure,[S9,S10] 1.0 equiv.) in anhydrous acetonitrile was added 1,8-diazabicyclo[5.4.0]undec-7-ene (DBU, 1.8 equiv.) and iodomethane (3.0 equiv.) at room temperature. The mixture was then stirred at room temperature for 10 hours and extracted with ethyl acetate. The combined organic layers were washed with brine and dried over Na_2SO_4, and the solvents were evaporated *in vacuo*. The residue was purified by silica gel column chromatography to afford compounds **1f**, **1h**, and **1i**.

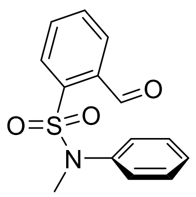

1a: The compound was synthesized from *N*-methylaniline (200 mg, 1.9 mmol) and 2-formylbenzenesulfonyl chloride (593 mg, 2.9 mmol) according to the general route 1. The crude product was purified by silica gel column chromatography using petroleum ether/ethyl acetate (10:1, v/v) as eluent to give the product as a white solid (449 mg, 86%). ^{1}H NMR ($CDCl_3$): δ 9.81 (s, 1H), 8.03-8.00 (m, 1H), 7.97-7.94 (m, 1H), 7.76-7.69 (m, 2H), 7.33-7.31 (m, 3H), 7.08-7.06 (m, 2H), 3.21 (s, 3H). ^{13}C NMR ($CDCl_3$): δ 189.7, 140.1, 136.9, 134.8, 133.3, 133.3, 130.4, 129.5, 129.1, 128.5, 127.3, 38.4. ESI-HRMS: *m/z* calculated for $C_{14}H_{13}NO_3SNa$ $[M + Na]^+$: 298.0514; found: 298.0511.

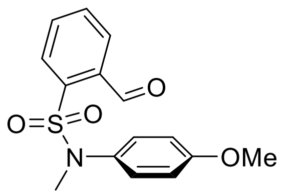

1b: The compound was synthesized from 4-methoxy-*N*-methylaniline (150 mg, 1.1 mmol) and 2-formylbenzenesulfonyl chloride (348 mg, 1.7 mmol) according to the general route 1. The crude product was purified by silica gel column chromatography using petroleum ether/ethyl acetate (10:1, v/v) as eluent to give the product as a white solid (299 mg, 89%). ^{1}H NMR ($CDCl_3$): δ 9.87 (s, 1H), 8.04-8.00 (m, 1H), 7.97-7.92 (m, 1H), 7.75-7.69 (m, 2H), 6.98-6.94 (m, 2H), 6.82-6.78 (m, 2H), 3.79 (s, 3H), 3.17 (s, 3H). ^{13}C NMR ($CDCl_3$): δ 189.9, 159.5, 137.2, 134.8, 133.3, 133.2, 132.6, 130.4, 129.0, 128.7, 114.6, 55.6, 38.7. ESI-HRMS: *m/z* calculated for $C_{15}H_{15}NO_4SNa$ $[M + Na]^+$: 328.0619; found: 328.0616.

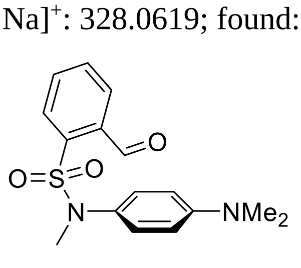

1c: The compound was synthesized from 4-(dimethylamino)-*N*-methylaniline (150 mg, 1.0 mmol) and 2-formylbenzenesulfonyl chloride (307 mg, 1.5 mmol) according to the

general route 1. The crude product was purified by silica gel column chromatography using petroleum ether/ethyl acetate (5:1, v/v) as eluent to give the product as a yellow solid (239 mg, 75%). ^{1}H NMR ($CDCl_3$): δ 9.78 (s, 1H), 8.02 (d, *J* = 7.2, 1H), 7.97 (d, *J* = 7.2, 1H), 7.75-7.68 (m, 2H), 6.87 (d, *J* = 8.8 Hz, 2H), 6.56 (d, *J* = 8.8 Hz, 2H), 3.16 (s, 3H), 2.94 (s, 6H). ^{13}C NMR ($CDCl_3$): δ 190.0, 150.2, 137.3, 134.7, 133.1, 132.8, 130.3, 128.7, 128.2, 127.9, 112.2, 40.4, 38.6. ESI-HRMS: *m/z* calculated for $C_{16}H_{18}N_2O_3SNa$ $[M + Na]^+$: 341.0936; found: 341.0939.

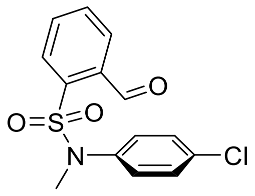

1d: The compound was synthesized from 4-chloro-*N*-methylaniline (200 mg, 1.4 mmol) and 2-formylbenzenesulfonyl chloride (430 mg, 2.1 mmol) according to the general route 1. The crude product was purified by silica gel column chromatography using petroleum ether/ethyl acetate (10:1, v/v) as eluent to give the product as a white solid (403 mg, 93%). ^{1}H NMR ($CDCl_3$): δ 10.03 (s, 1H), 8.04-8.01 (m, 1H), 7.91-7.88 (m, 1H), 7.74-7.72 (m, 2H), 7.28 (d, *J* = 8.4 Hz, 2H), 7.01 (d, *J* = 8.4 Hz, 2H), 3.17 (s, 3H). ^{13}C NMR ($CDCl_3$): δ 189.6, 138.6, 136.8, 134.6, 134.1, 133.4, 133.3, 130.4, 129.5, 129.2, 128.3, 38.2. ESI-HRMS: *m/z* calculated for $C_{14}H_{12}NO_3SClNa$ $[M + Na]^+$: 332.0124; found: 332.0121.

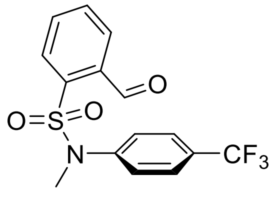

1e: The compound was synthesized from 4-trifluoromethyl-*N*-methylaniline (100 mg, 0.6 mmol) and 2-formylbenzenesulfonyl chloride (184 mg, 0.9 mmol) according to the general route 1. The crude product was purified by silica gel column chromatography using petroleum ether/ethyl acetate (5:1, v/v) as eluent to give the product as a white solid (152 mg, 74%). ^{1}H NMR ($CDCl_3$): δ 10.11 (s, 1H), 8.05-8.02 (m, 1H), 7.89-7.87 (m, 1H), 7.75-7.72 (m, 2H), 7.59 (d, *J* = 8.4 Hz, 2H), 7.23 (d, *J* = 8.4 Hz, 2H), 3.23 (s, 3H). ^{19}F NMR ($CDCl_3$): δ -62.6. ^{13}C NMR ($CDCl_3$): δ 189.4, 143.4, 136.8, 134.6, 133.5,

133.4, 130.3, 130.0, 129.3, 126.9, 126.5, 123.6, 38.0. ESI-HRMS: *m/z* calculated for $C_{15}H_{12}F_3NO_3SNa$ [M + Na]$^+$: 366.0388; found: 366.0385.

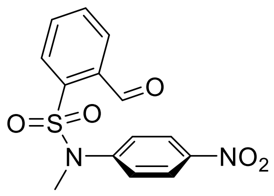

1f: The compound was synthesized from **3f** (150 mg, 0.5 mmol) and iodomethane (213 mg, 1.5 mmol) according to the general route 2. The crude product was purified by silica gel column chromatography using petroleum ether/ethyl acetate (5:1, v/v) as eluent to give the product as a yellow solid (139 mg, 87%). ^{1}H NMR ($CDCl_3$): δ 10.18 (s, 1H), 8.20 (d, *J* = 8.8 Hz, 2H), 8.05-8.02 (m, 1H), 7.88-7.86 (m, 1H), 7.78-7.72 (m, 2H), 7.31 (d, *J* = 8.8 Hz, 2H), 3.26 (s, 3H). ^{13}C NMR ($CDCl_3$): δ 189.4, 146.4, 146.0, 136.8, 134.7, 133.9, 133.6, 130.3, 129.7, 126.7, 124.8, 37.9. ESI-HRMS: *m/z* calculated for $C_{14}H_{12}N_2O_5SNa$ [M + Na]$^+$: 343.0365; found: 343.0361.

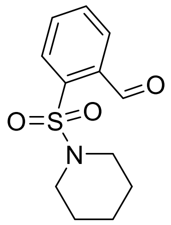

1g: The compound was synthesized from piperidine (150 mg, 1.8 mmol) and 2-formylbenzenesulfonyl chloride (552 mg, 2.7 mmol) according to the general route 1. The crude product was purified by silica gel column chromatography using petroleum ether/ethyl acetate (10:1, v/v) as eluent to give the product as a white solid (365 mg, 80%). ^{1}H NMR ($CDCl_3$): δ 10.81 (s, 1H), 8.07-8.05 (m, 1H), 7.95-7.93 (m, 1H), 7.74-7.69 (m, 2H), 3.07 (t, *J* = 5.6 Hz, 4H), 1.61 (quin, *J* = 5.6 Hz, 4H), 1.47 (quin, *J* = 5.6 Hz, 2H). ^{13}C NMR ($CDCl_3$): δ 190.9, 138.4, 134.4, 133.2, 133.1, 130.1, 129.2, 46.2, 25.1, 23.5. ESI-HRMS: *m/z* calculated for $C_{12}H_{15}NO_3SNa$ [M + Na]$^+$: 276.0670; found: 276.0673.

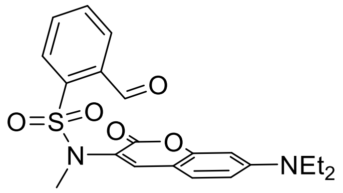

1h: The compound was synthesized from **3h** (260 mg, 0.6 mmol) and iodomethane (255 mg, 1.8 mmol) according to the general route 2. The crude product was purified by silica gel column chromatography using petroleum ether/ethyl acetate (3:1, v/v) as eluent to give the product as a yellow solid (189 mg, 76%). ^{1}H NMR (CD_3CN): δ 10.52 (s, 1H), 8.01-7.97 (m, 2H), 7.84 (s, 1H), 7.79-7.75 (m, 2H), 7.39 (d, J = 8.8 Hz, 1H), 6.72 (dd, J = 9.2, 2.4 Hz, 1H), 6.49 (d, J = 2.4 Hz, 1H), 3.45 (q, J = 7.2 Hz, 4H), 3.19 (s, 3H), 1.16 (t, J = 6.8 Hz, 6H). ^{13}C NMR ($CDCl_3$): δ 190.3, 159.0, 156.3, 151.5, 145.4, 139.6, 134.4, 133.2, 133.1, 129.9, 129.8, 129.3, 118.0, 109.5, 107.4, 97.0, 44.9, 36.8, 12.4. ESI-HRMS: *m/z* calculated for $C_{21}H_{22}N_2O_5SNa$ $[M + Na]^+$: 437.1147; found: 437.1145.

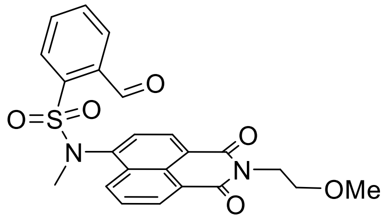

1i: The compound was synthesized from **3i** (100 mg, 0.2 mmol) and iodomethane (85 mg, 0.6 mmol) according to the general route 2. The crude product was purified by silica gel column chromatography using petroleum ether/ethyl acetate (3:1, v/v) as eluent to give the product as a yellow solid (62 mg, 69%). ^{1}H NMR (DMSO-d_6): δ 9.89 (s, 1H), 8.58-8.55 (m, 2H), 8.38 (d, J = 8.0 Hz, 1H), 7.99-7.95 (m, 5H), 7.41 (d, J = 8.0 Hz, 1H), 4.25 (t, J = 6.4 Hz, 2H), 3.59 (t, J = 6.4 Hz, 2H), 3.39 (s, 3H), 3.26 (s, 3H). ^{13}C NMR (DMSO-d_6): δ 190.6, 163.7, 163.2, 143.0, 136.5, 134.9, 134.8, 132.0, 131.1, 131.0, 130.9, 130.6, 129.6, 129.0, 128.5, 127.0, 122.9, 122.8, 69.0, 58.4. ESI-HRMS: *m/z* calculated for $C_{23}H_{20}N_2O_6SNa$ $[M + Na]^+$: 475.0940; found: 475.0942.

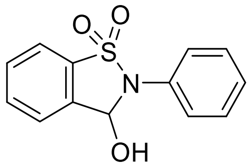

3a: The reported procedure[S9] was used to afford the title compound as a white solid (yield: 82%).

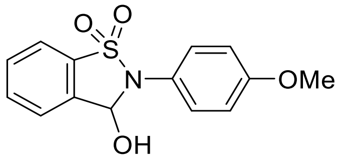

3b: The reported procedure[S9] was used to afford the title compound as a white solid (yield: 80%).

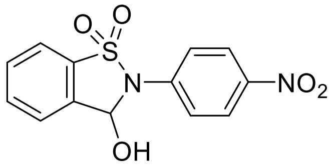

3f: The compound was synthesized from 4-nitroaniline (200 mg, 1.4 mmol) and 2-formylbenzenesulfonyl chloride (429 mg, 2.1 mmol) according to the literature procedure.[S9] The crude product was purified by silica gel column chromatography using petroleum ether/ethyl acetate (3:1, v/v) as eluent to give the product as a yellow solid (300 mg, 70%). ^{1}H NMR (CD_3CN): δ 8.33 (d, *J* = 8.8 Hz, 2H), 7.94 (d, *J* = 7.6 Hz, 1H), 7.90-7.86 (m, 1H), 7.80-7.76 (m, 2H), 7.71 (d, *J* = 8.8 Hz, 2H), 6.65 (d, *J* = 8.8 Hz, 1H), 5.27 (d, *J* = 8.8 Hz, 1H). ^{13}C NMR (DMSO-d_6): δ 142.6, 141.9, 135.7, 134.6, 133.3, 131.4, 126.0, 125.4, 120.8, 118.3, 80.4. ESI-HRMS: *m/z* calculated for $C_{13}H_{10}N_2O_5SNa$ [M + Na]$^+$: 329.0208; found: 329.0206.

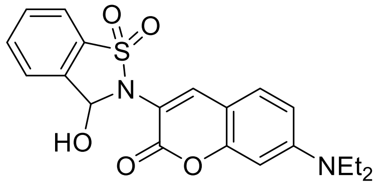

3h: The reported procedure[S10] was used to afford the title compound as a yellow solid (yield: 70%).

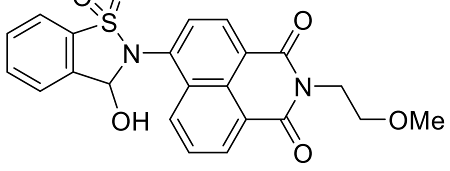

3i: The reported procedure[S10] was used to afford the title compound as a yellow solid (yield: 75%).

Scheme S2. General synthetic routes of **6f** and **6h**.

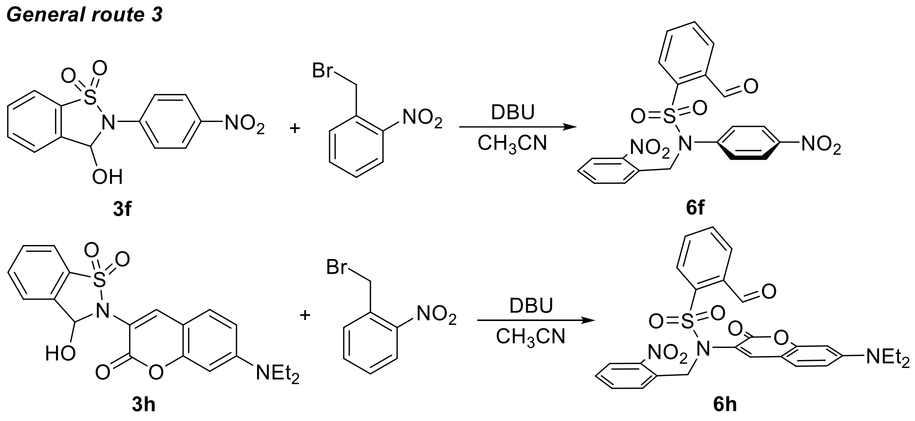

***General route 3*:** To a solution of 2-formylbenzenesulfonamide **3** (1.0 equiv.) in anhydrous acetonitrile was added 1,8-diazabicyclo[5.4.0]undec-7-ene (DBU, 1.8 equiv.) and 2-nitrophenylmethyl bromide (1.5 equiv.) at room temperature. The mixture was then stirred at room temperature for 10 hours and extracted with ethyl acetate. The combined organic layers were washed with brine and dried over Na_2SO_4, and the solvents were evaporated *in vacuo*. The residue was purified by silica gel column chromatography to afford compounds **6f** and **6h**.

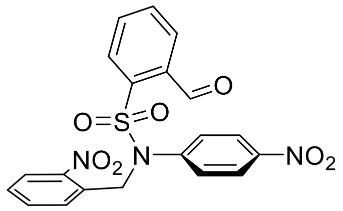

6f: The compound was synthesized from **3f** (240 mg, 0.8 mmol) and 2-nitrophenylmethyl bromide (259 mg, 1.2 mmol) according to the general route 3. The crude product was purified by silica gel column chromatography using petroleum ether/ethyl acetate (5:1, v/v) as eluent to give the product as a white solid (258 mg, 73%). ^{1}H NMR ($CDCl_3$): δ 10.11 (s, 1H), 8.14 (d, *J* = 9.2 Hz, 2H), 8.07-8.04 (m, 1H), 7.99-7.95 (m, 2H), 7.83-7.78 (m, 3H), 7.66 (t, *J* = 7.6 Hz, 1H), 7.45 (t, *J* = 7.6 Hz, 1H), 7.30 (d, *J* = 9.2 Hz, 2H), 5.26 (s, 2H). ^{13}C NMR ($CDCl_3$): δ 189.0, 148.2, 146.9, 143.8, 137.7, 134.5, 134.1, 133.9, 133.8, 130.7, 130.2, 129.9, 129.8, 129.2, 128.6, 125.2, 124.9, 51.4. ESI-HRMS: *m/z* calculated for $C_{20}H_{15}N_3O_7SNa$ $[M + Na]^+$: 464.0528; found: 464.0525.

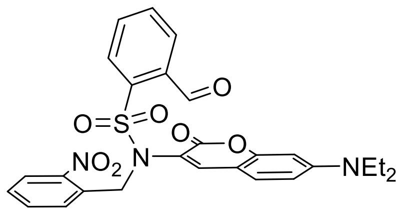

6h: The compound was synthesized from **3h** (260 mg, 0.6 mmol) and 2-nitrophenylmethyl bromide (194 mg, 0.9 mmol) according to the general route 3. The crude product was purified by silica gel column chromatography using petroleum ether/ethyl acetate (3:1, v/v) as eluent to give the product as a yellow solid (205 mg, 64%). ^{1}H NMR ($CDCl_3$): δ 10.57 (s, 1H), 8.08-8.05 (m, 2H), 7.90 (d, *J* = 8.0 Hz, 1H), 7.84 (d, *J* = 8.0 Hz, 1H), 7.74-7.70 (m, 3H), 7.63 (t, *J* = 7.6 Hz, 1H), 7.42 (t, *J* = 7.6 Hz, 1H), 7.24 (d, *J* = 9.2 Hz, 1H), 6.57 (dd, *J* = 9.2, 2.4 Hz, 1H), 6.36 (d, *J* = 2.4 Hz, 1H), 5.26 (s, 2H), 3.39 (q, *J* = 7.2 Hz, 4H), 1.19 (t, *J* = 7.2 Hz, 6H). ^{13}C NMR ($CDCl_3$): δ 190.0, 158.9, 156.4, 151.7, 148.9, 146.8, 139.5, 134.4, 133.5, 133.4, 133.4, 131.6, 131.1, 130.0, 130.0, 129.4, 128.8, 124.8, 115.4, 109.5, 107.2, 97.0, 49.4, 44.9, 12.4. ESI-HRMS: *m/z* calculated for $C_{27}H_{25}N_3O_7SNa$ $[M + Na]^+$: 558.1131; found: 558.1133.

Scheme S3. Synthetic route of **8i**.

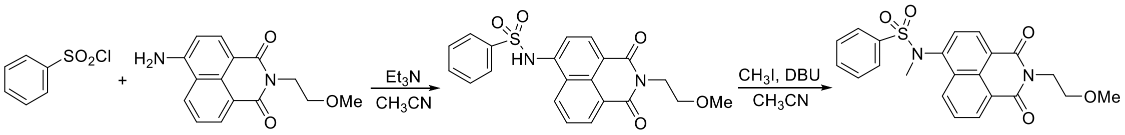

7i

8i

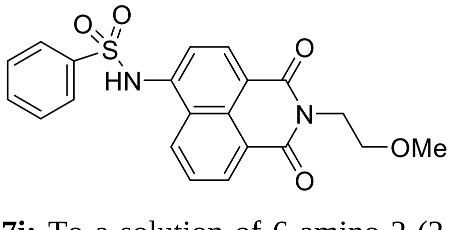

7i: To a solution of 6-amino-2-(2-methoxyethyl)-1H-benzo[de]isoquinoline-1,3(2H)-dione (200 mg, 0.7 mmol) in anhydrous acetonitrile (10 mL) was added triethylamine (0.1 ml, 0.7 mmol). The mixed solution was then added to a stirring solution of benzenesulfonyl chloride (194 mg, 1.1 mmol) in acetonitrile dropwise. The mixture was stirred at room temperature for 10 hours and extracted with ethyl acetate (3 × 20 mL). The combined organic layers were washed with brine and dried over Na_2SO_4, and

the solvents were evaporated *in vacuo*. The crude product was purified by silica gel column chromatography using petroleum ether/ethyl acetate (2:1, v/v) as eluent to give the product as a yellow solid (172 mg, 60%). ^{1}H NMR ($CDCl_3$): δ 8.59 (d, *J* = 7.2 Hz, 1H), 8.47 (d, *J* = 8.0 Hz, 1H), 8.17 (d, *J* = 8.4 Hz, 1H), 7.82 (d, *J* = 7.6 Hz, 2H), 7.72 (t, *J* = 8.0 Hz, 1H), 7.62 (d, *J* = 8.0 Hz, 1H), 7.58-7.52 (m, 1H), 7.45 (t, *J* = 7.6 Hz, 1H), 7.30 (br, 1H), 4.41 (t, *J* = 5.6 Hz, 2H), 3.72 (t, *J* = 5.6 Hz, 2H), 3.37 (s, 3H). ^{13}C NMR ($CDCl_3$): δ 164.0, 163.5, 138.5, 138.0, 133.7, 131.6, 129.4, 128.8, 127.5, 127.3, 127.1, 125.3, 122.8, 119.7, 119.5, 69.7, 58.7, 39.2. ESI-HRMS: *m/z* calculated for $C_{21}H_{18}N_2O_5SNa$ [M + Na]$^+$: 433.0834; found: 433.0836.

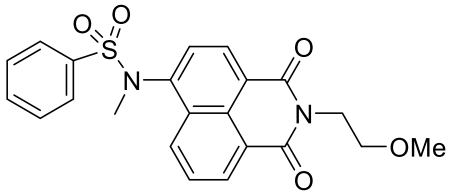

8i: To a solution of **7i** (170 mg, 0.4 mmol) in anhydrous acetonitrile (10 mL) was added 1,8-diazabicyclo[5.4.0]undec-7-ene (DBU, 0.2 ml, 0.9 mmol) and iodomethane (170 mg, 1.2 mmol) at room temperature. The mixture was then stirred at room temperature for 10 hours and extracted with ethyl acetate (3 × 20 mL). The combined organic layers were washed with brine and dried over Na_2SO_4, and the solvents were evaporated *in vacuo*. The crude product was purified by silica gel column chromatography using petroleum ether/ethyl acetate (3:1, v/v) as eluent to give the product as a yellow solid (139 mg, 82%). ^{1}H NMR ($CDCl_3$): δ 8.69 (d, *J* = 8.4 Hz, 1H), 8.66 (d, *J* = 7.6 Hz, 1H), 8.44 (d, *J* = 7.6 Hz, 1H), 7.84 (t, *J* = 7.6 Hz, 1H), 7.70-7.66 (m, 3H), 7.57-7.53 (m, 2H), 6.98 (d, *J* = 7.6 Hz, 1H), 4.44 (t, *J* = 5.6 Hz, 2H), 3.72 (t, *J* = 5.6 Hz, 2H), 3.37 (s, 3H), 3.34 (s, 3H). ^{13}C NMR ($CDCl_3$): δ 164.1, 163.7, 144.4, 135.9, 133.5, 132.2, 131.1, 131.0, 130.7, 129.4, 129.2, 128.2, 127.6, 125.0, 122.6, 122.6, 69.6, 58.9, 39.9, 39.4. ESI-HRMS: *m/z* calculated for $C_{22}H_{20}N_2O_5SNa$ [M + Na]$^+$: 447.0991; found: 447.0996.

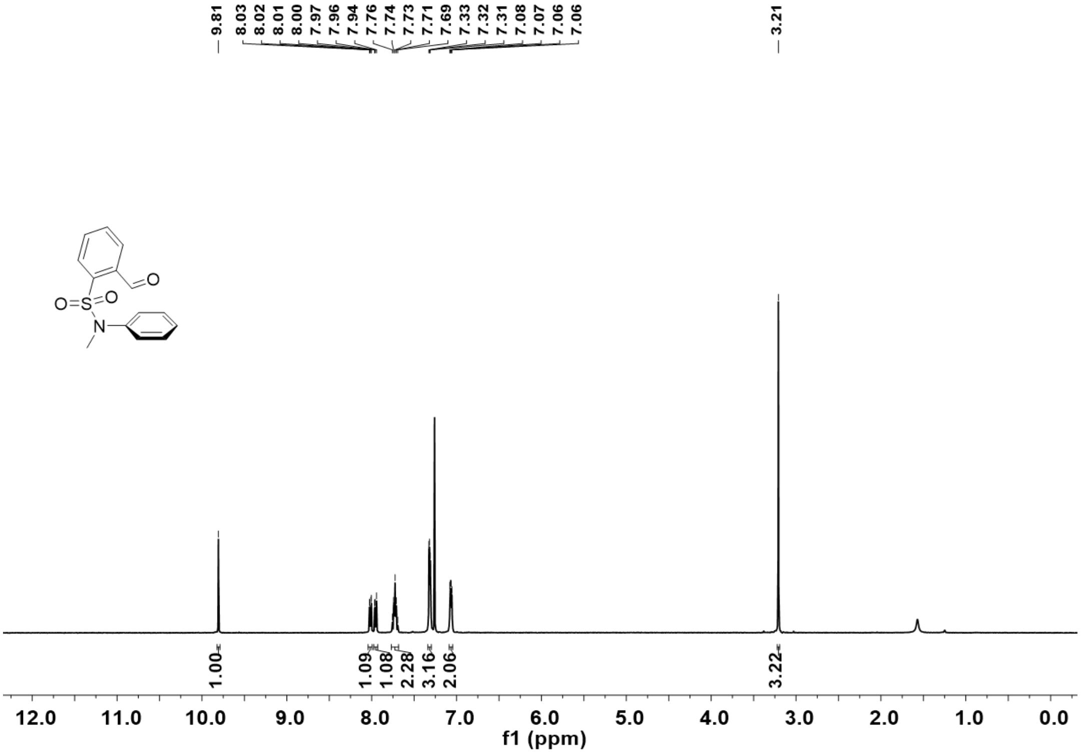

Figure S1. ^{1}H NMR spectrum of **1a** in $CDCl_3$.

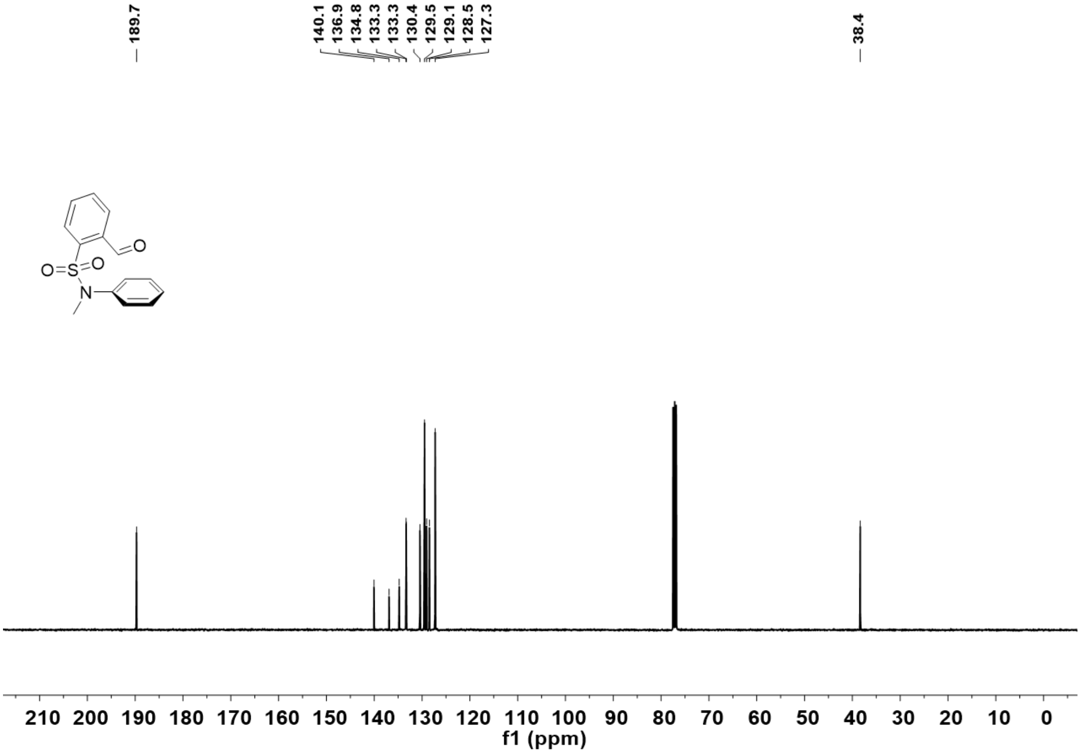

Figure S2. ^{13}C NMR spectrum of **1a** in $CDCl_3$.

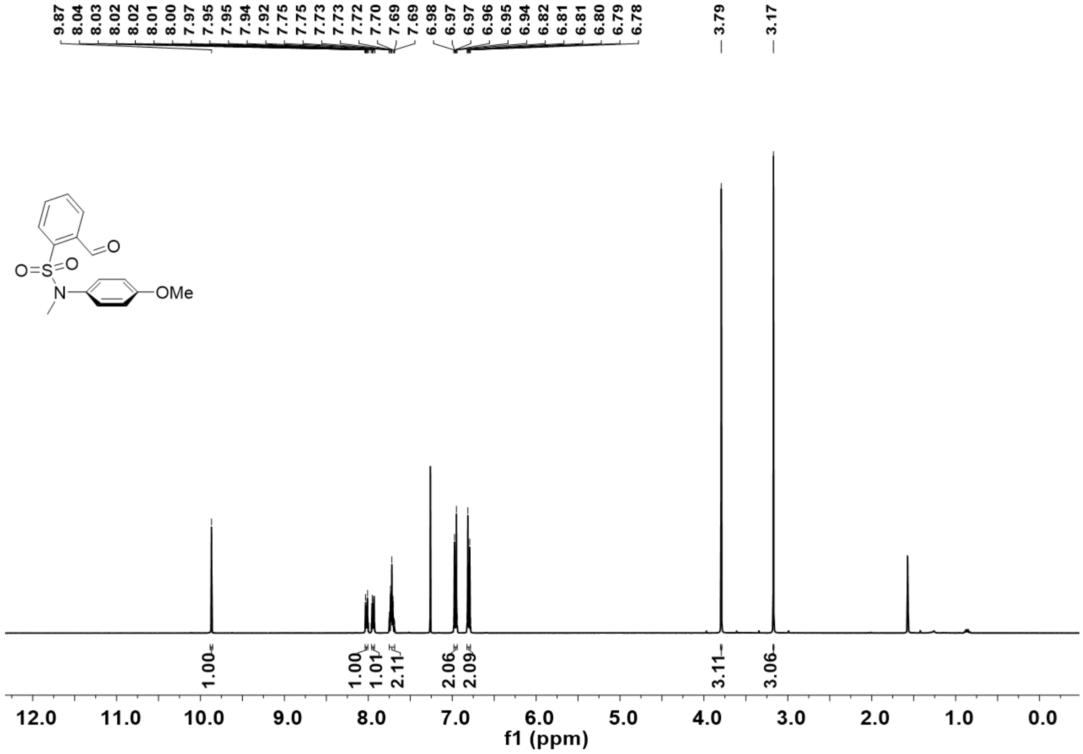

Figure S3. ^{1}H NMR spectrum of **1b** in $CDCl_3$.

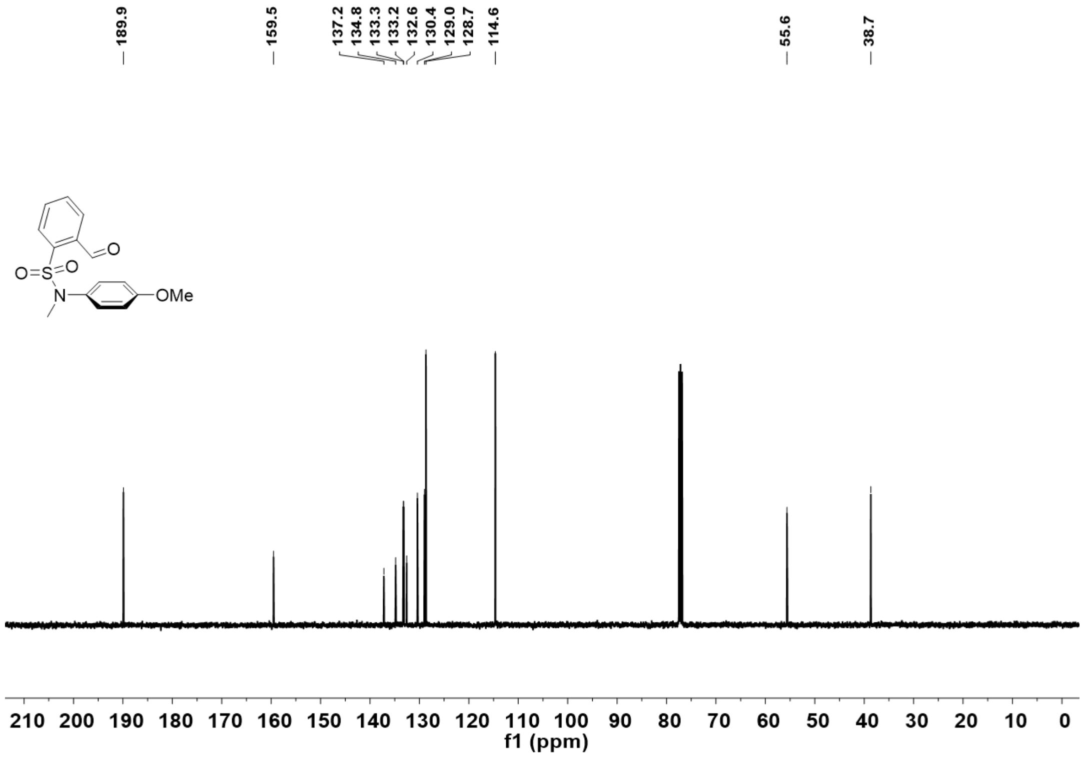

Figure S4. ^{13}C NMR spectrum of **1b** in $CDCl_3$.

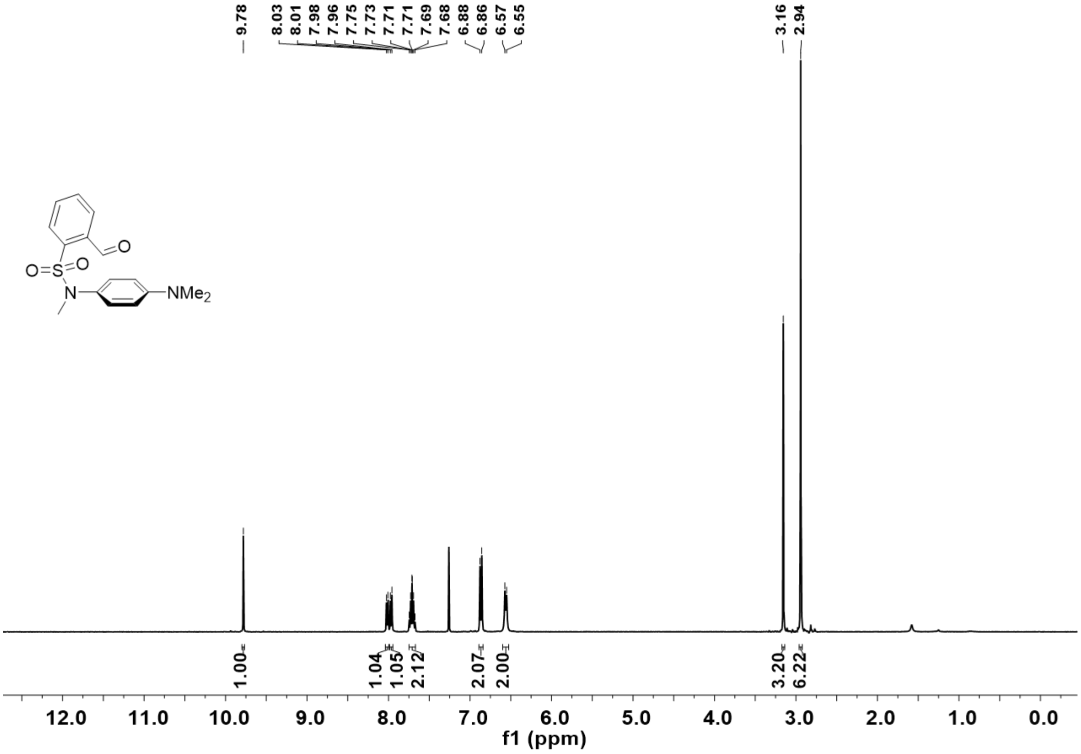

Figure S5. ^{1}H NMR spectrum of **1c** in $CDCl_3$.

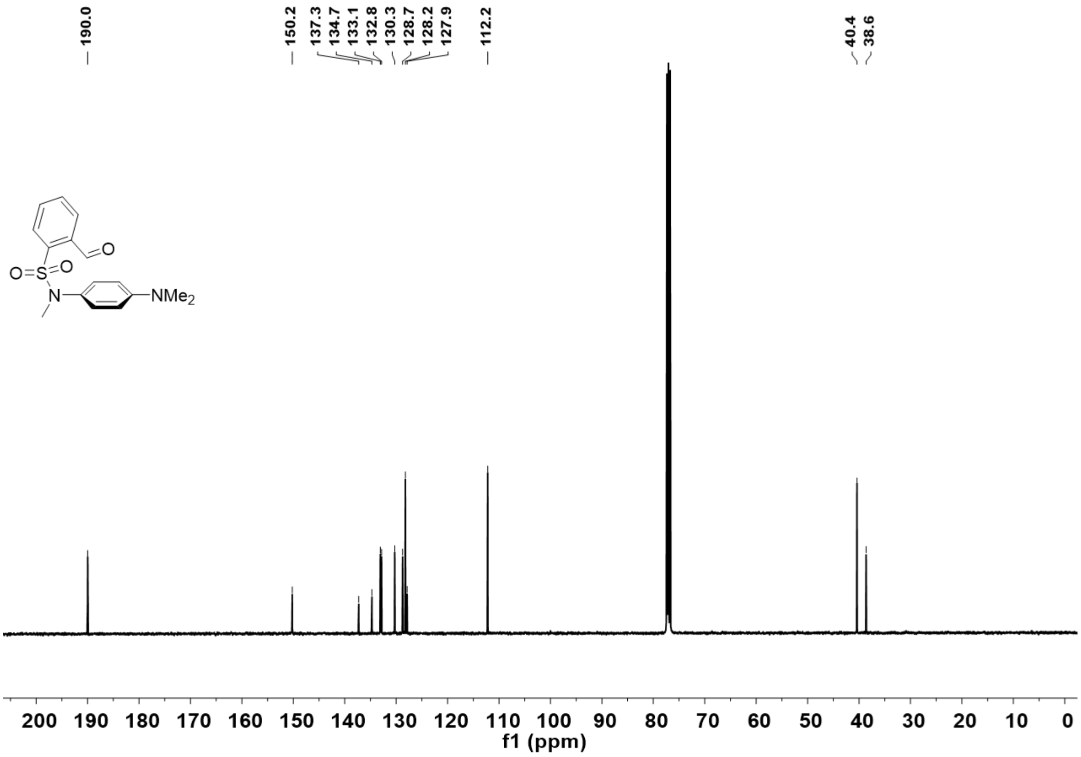

Figure S6. ^{13}C NMR spectrum of **1c** in $CDCl_3$.

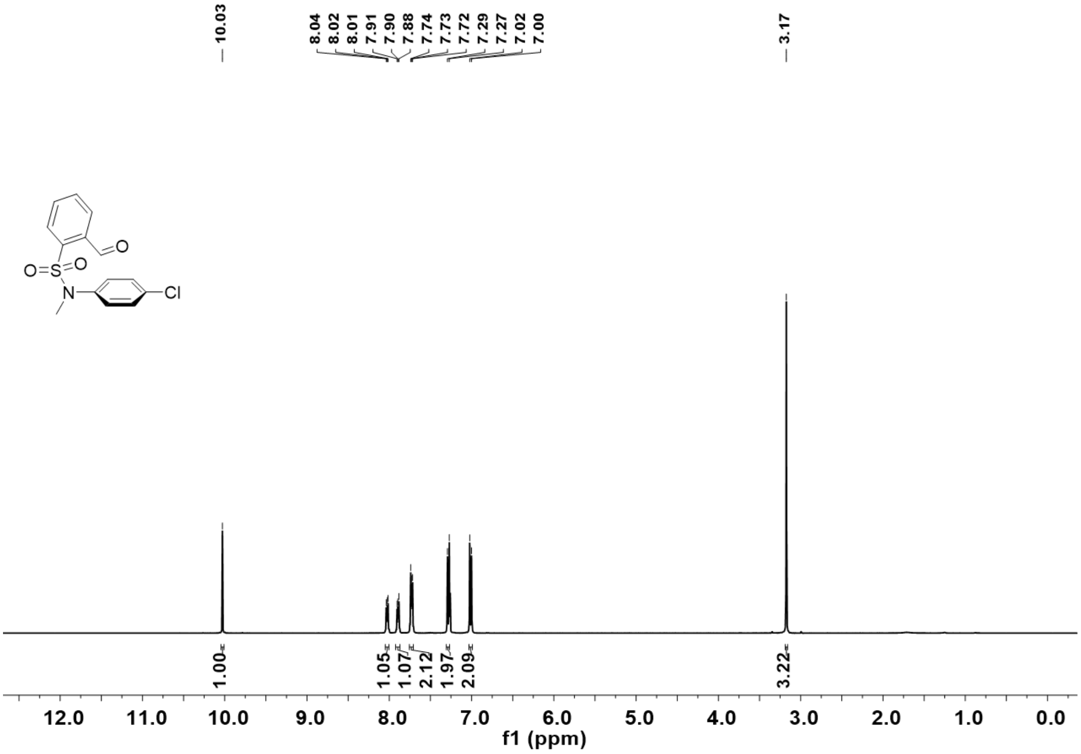

Figure S7. ^{1}H NMR spectrum of **1d** in $CDCl_3$.

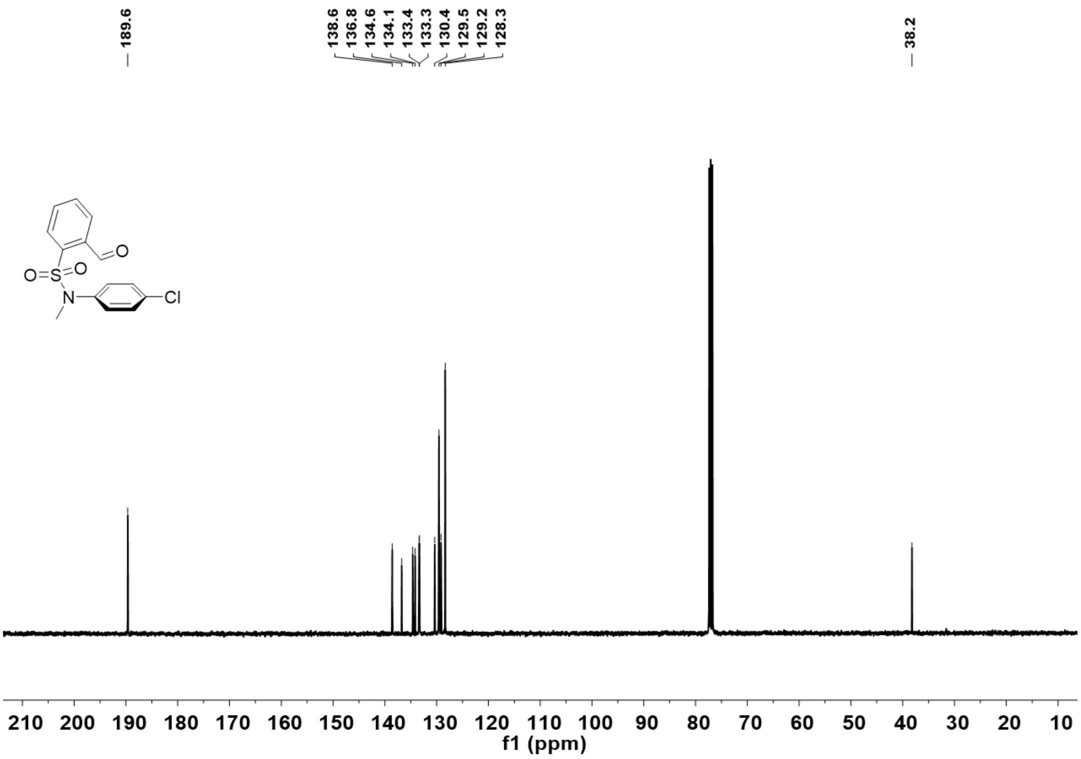

Figure S8. ^{13}C NMR spectrum of **1d** in $CDCl_3$.

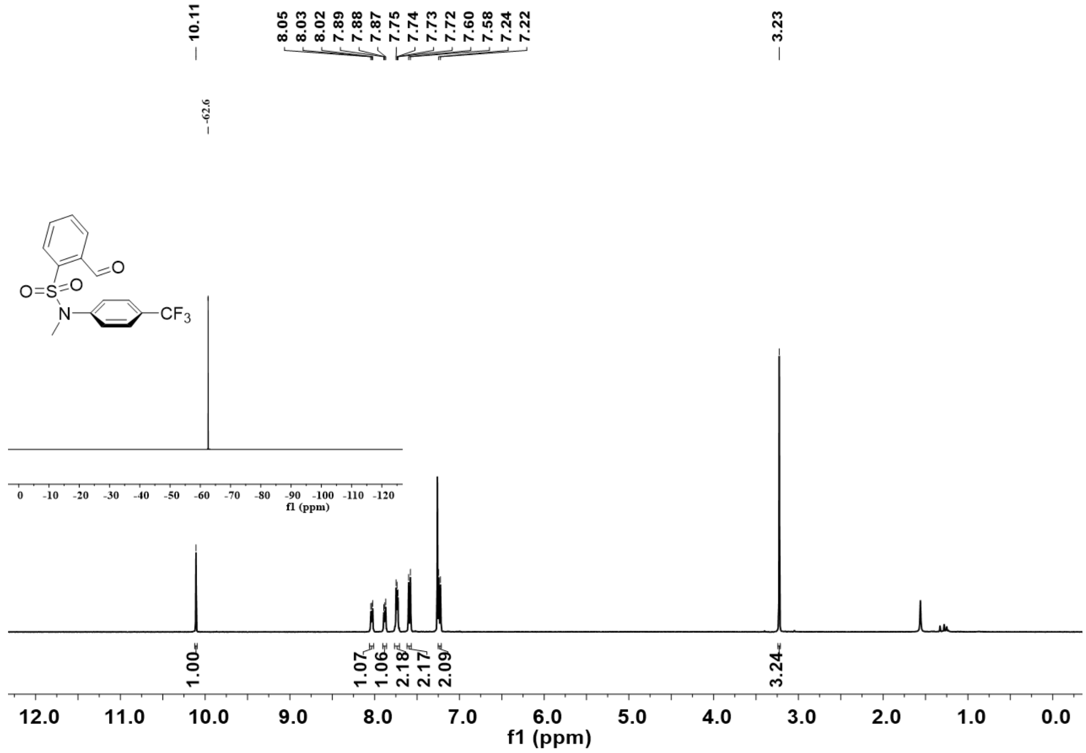

Figure S9. ^{1}H NMR spectrum of **1e** in $CDCl_3$. Inset: ^{19}F NMR spectrum of **1e** in $CDCl_3$.

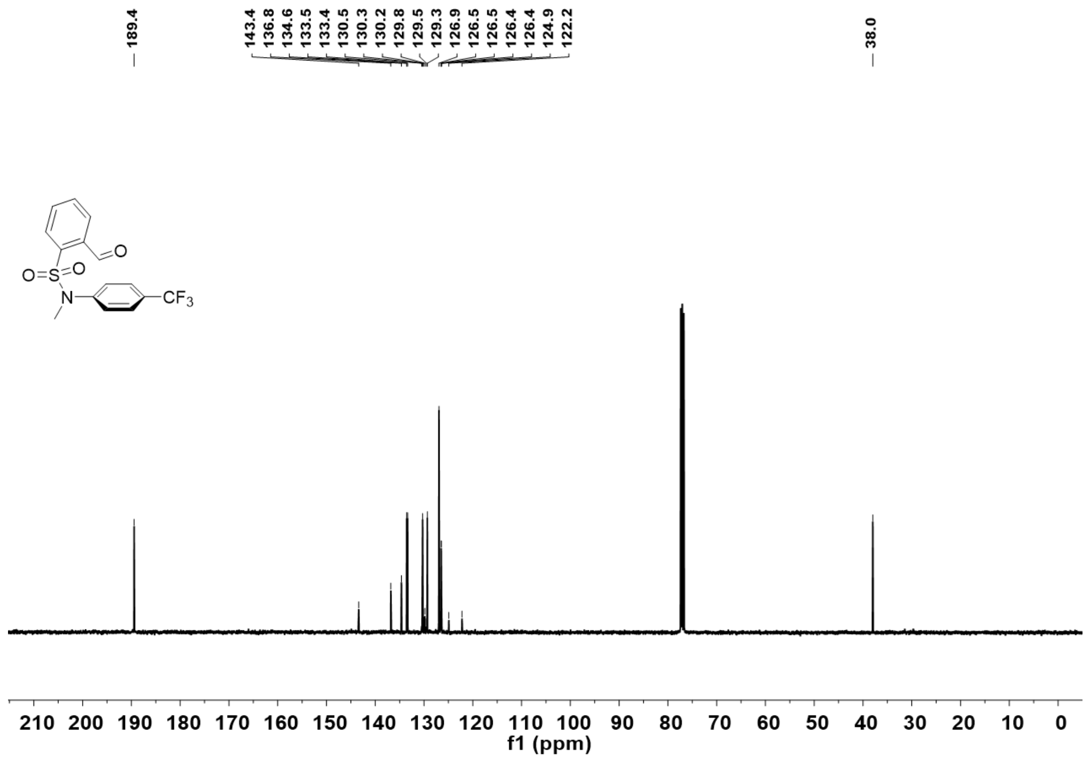

Figure S10. ^{13}C NMR spectrum of **1e** in $CDCl_3$.

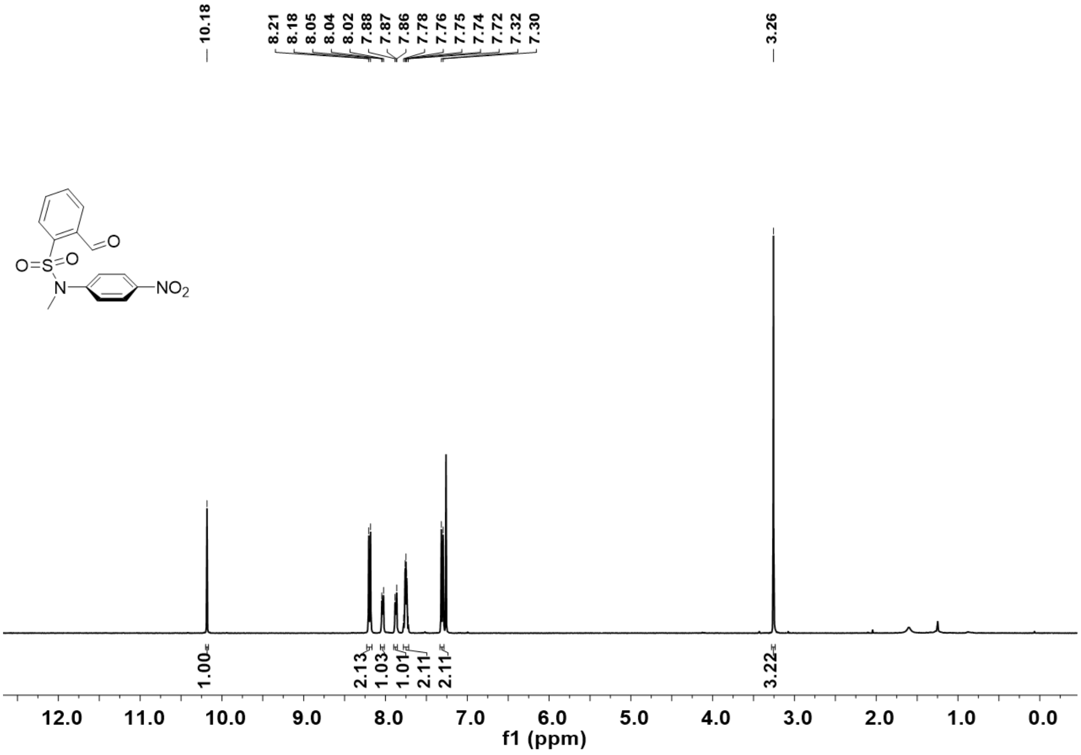

Figure S11. ^{1}H NMR spectrum of **1f** in $CDCl_3$.

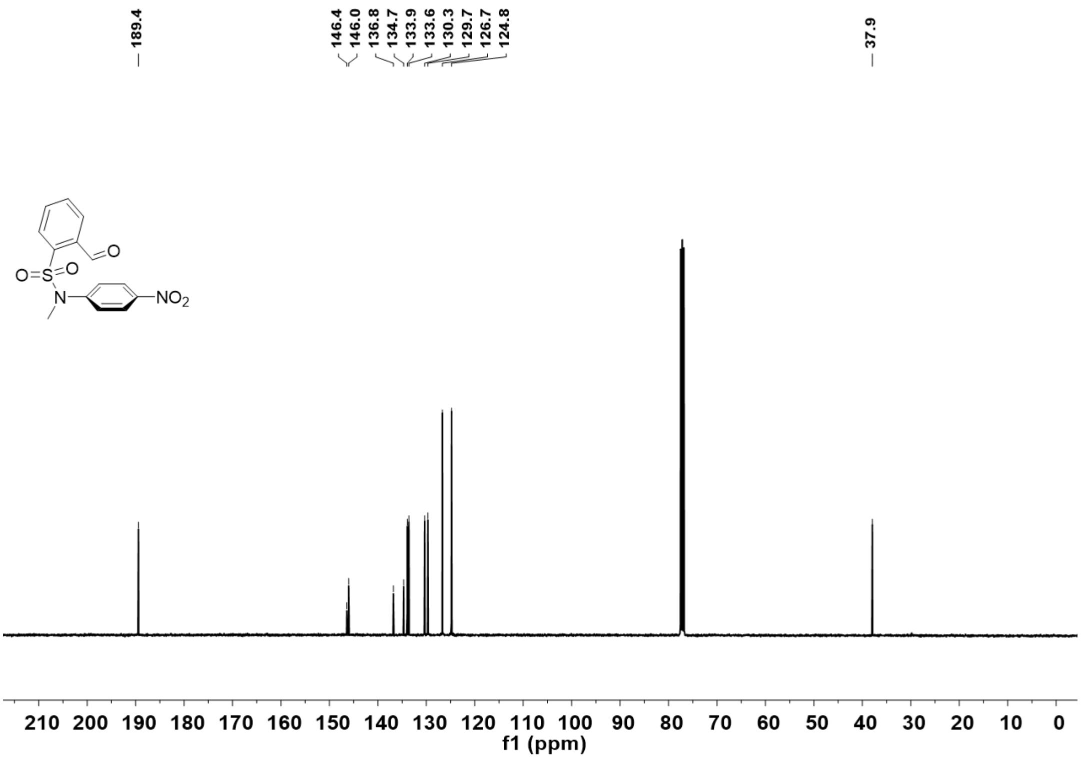

Figure S12. ^{13}C NMR spectrum of **1f** in $CDCl_3$.

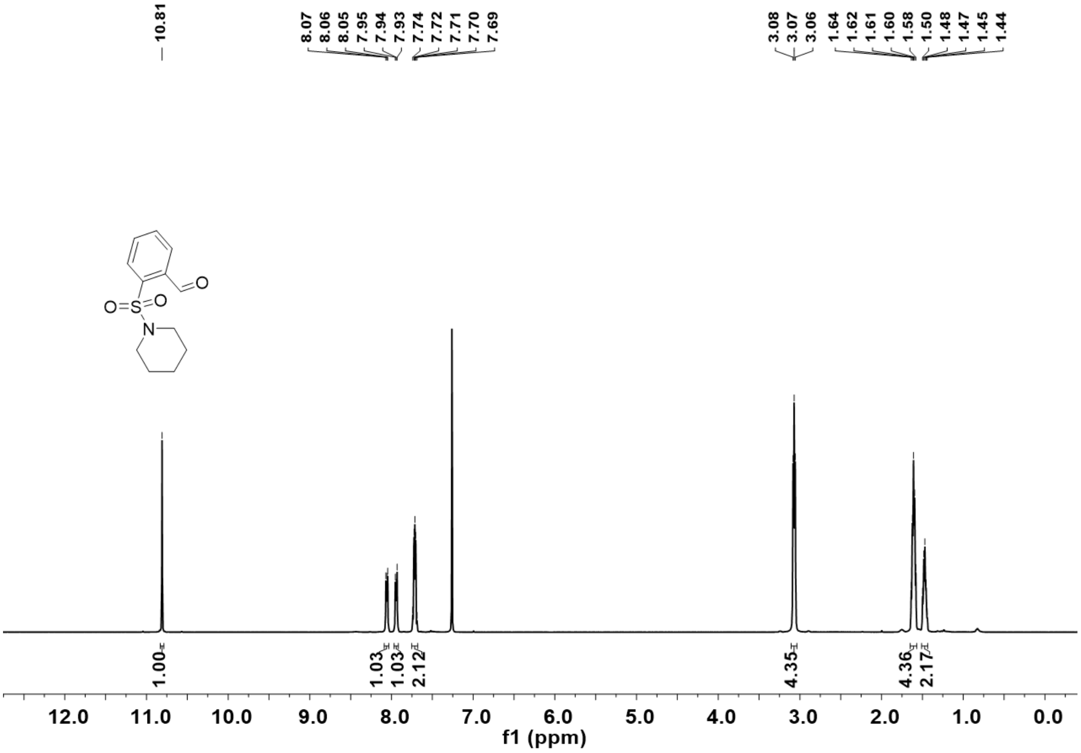

Figure S13. ^{1}H NMR spectrum of **1g** in $CDCl_3$.

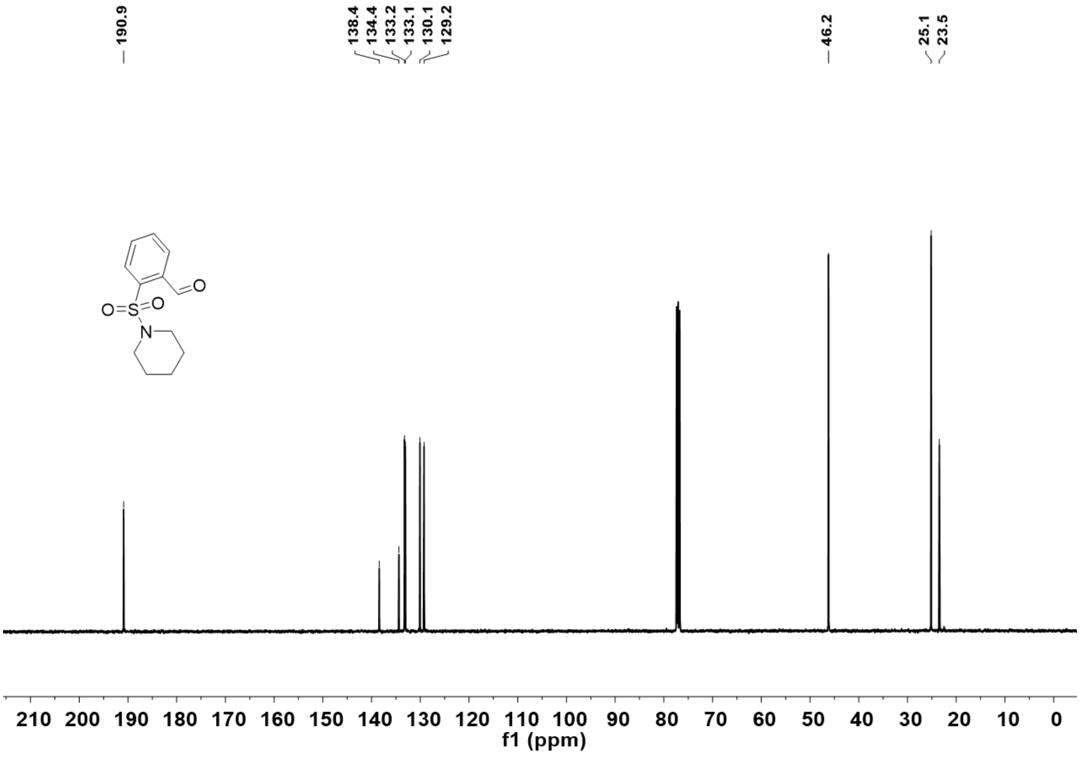

Figure S14. ^{13}C NMR spectrum of **1g** in $CDCl_3$.

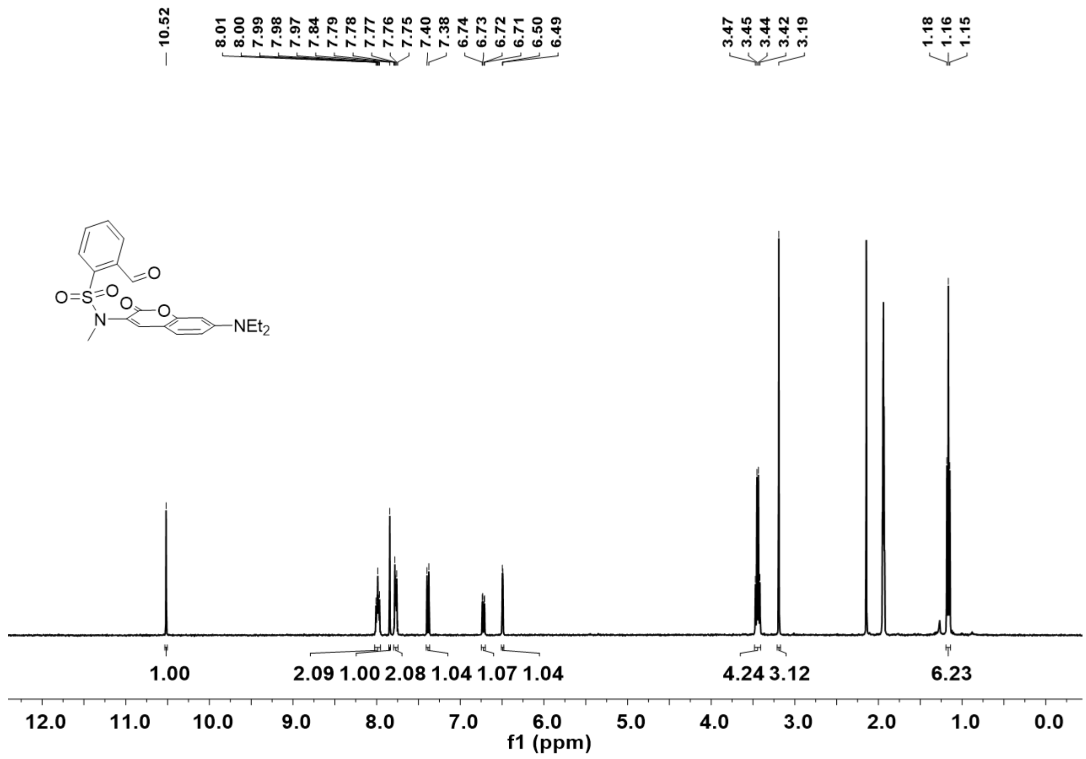

Figure S15. ^{1}H NMR spectrum of **1h** in CD_3CN.

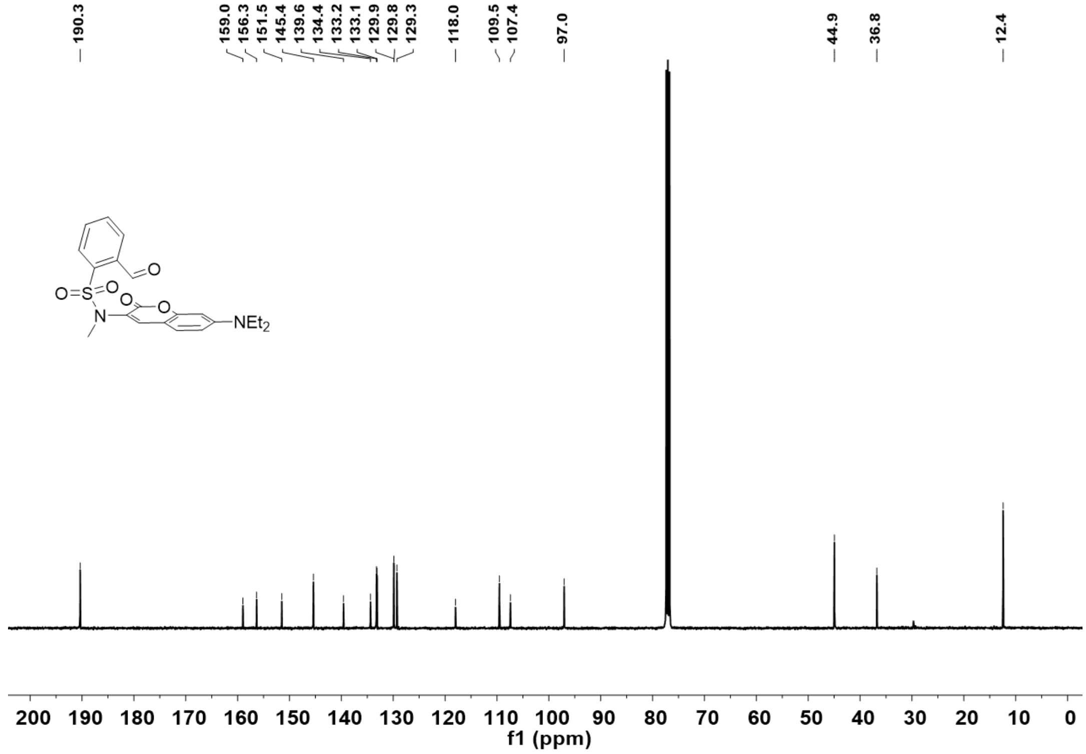

Figure S16. ^{13}C NMR spectrum of **1h** in $CDCl_3$.

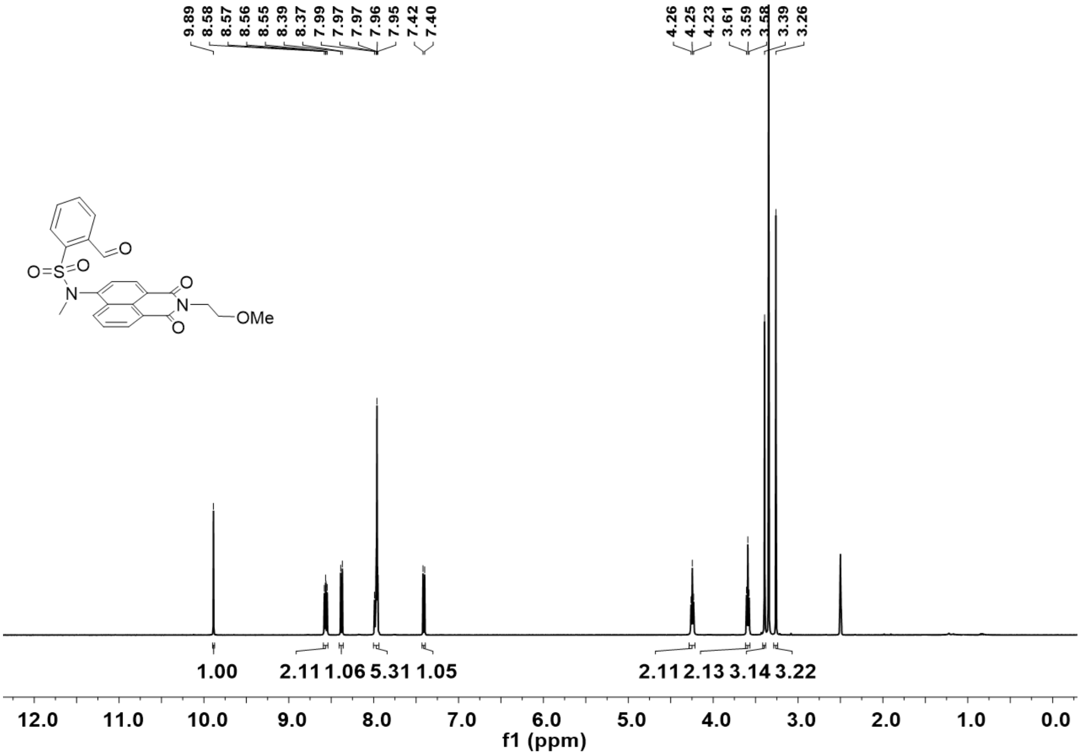

Figure S17. ^{1}H NMR spectrum of **1i** in DMSO-d_6.

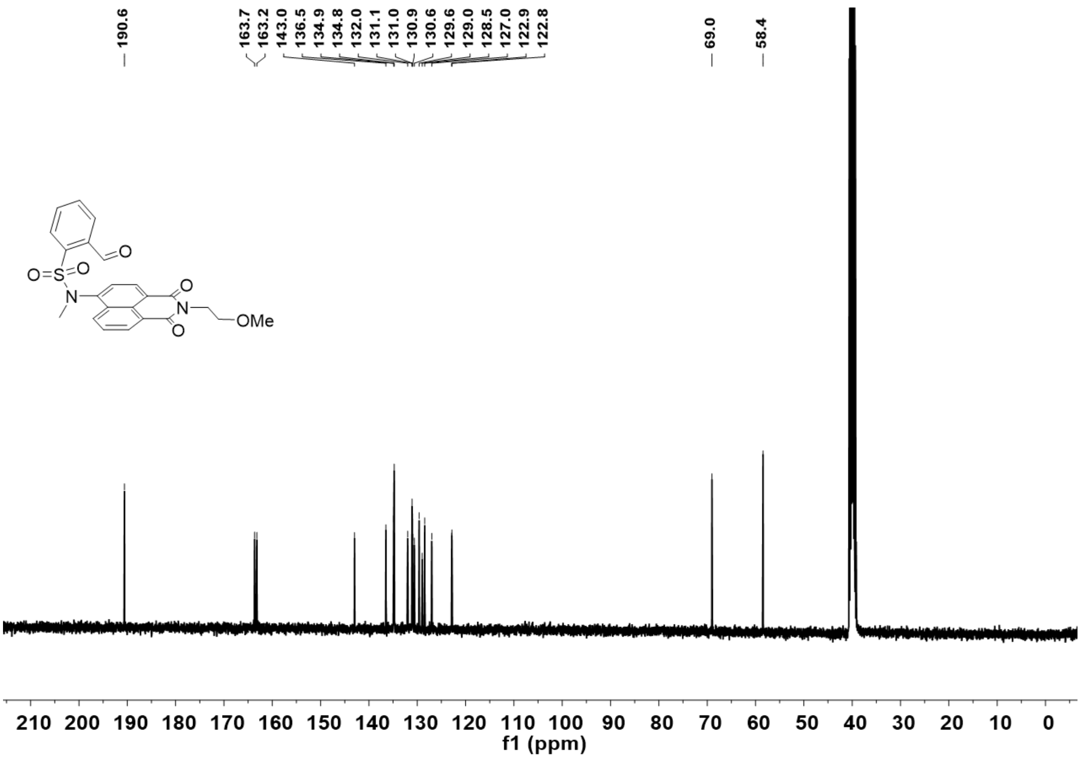

Figure S18. ^{13}C NMR spectrum of **1i** in DMSO-d_6.

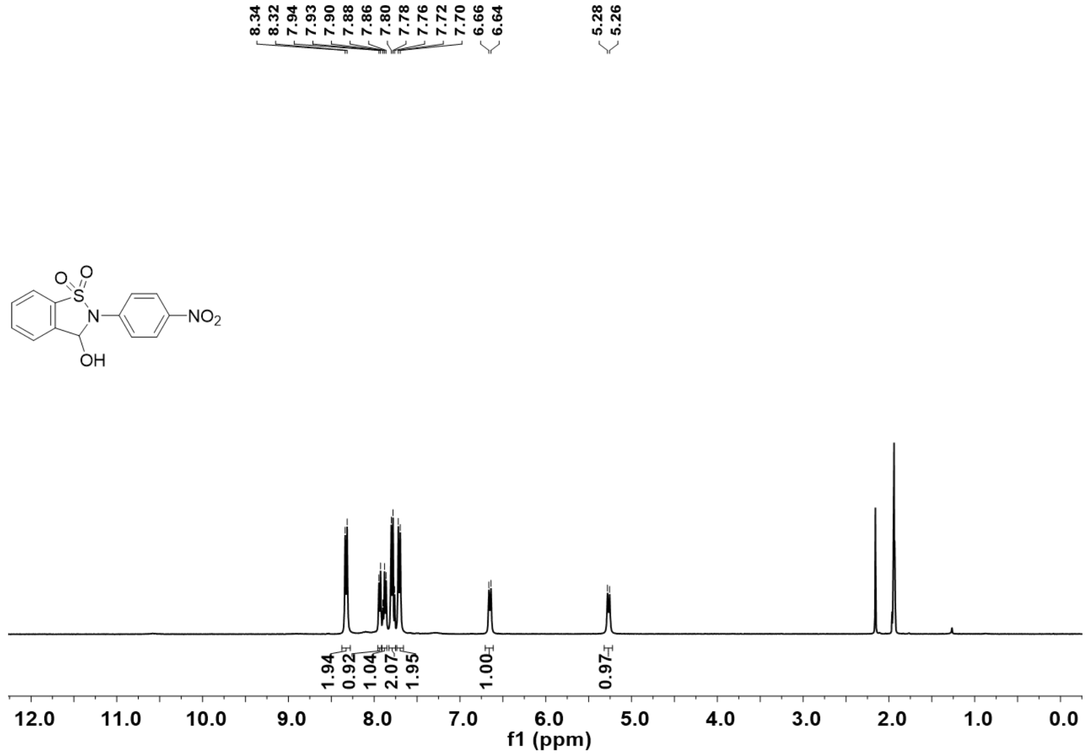

Figure S19. ^{1}H NMR spectrum of **3f** in CD_3CN.

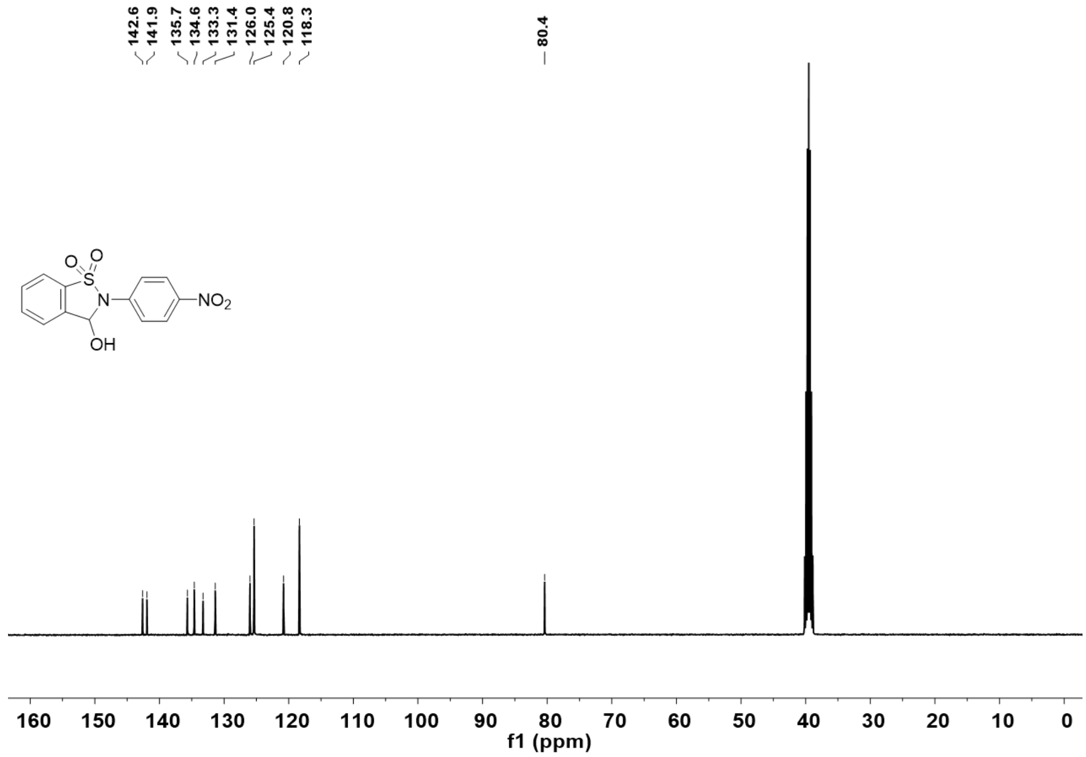

Figure S20. ^{13}C NMR spectrum of **3f** in DMSO-d_6.

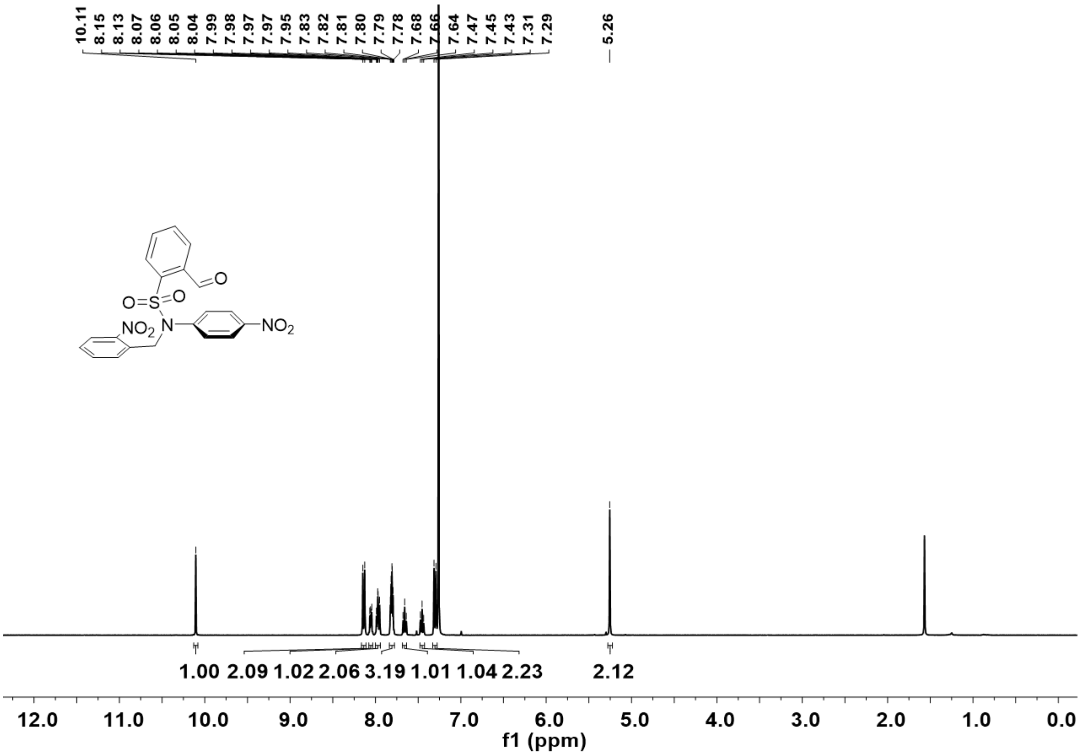

Figure S21. ^{1}H NMR spectrum of **6f** in $CDCl_3$.

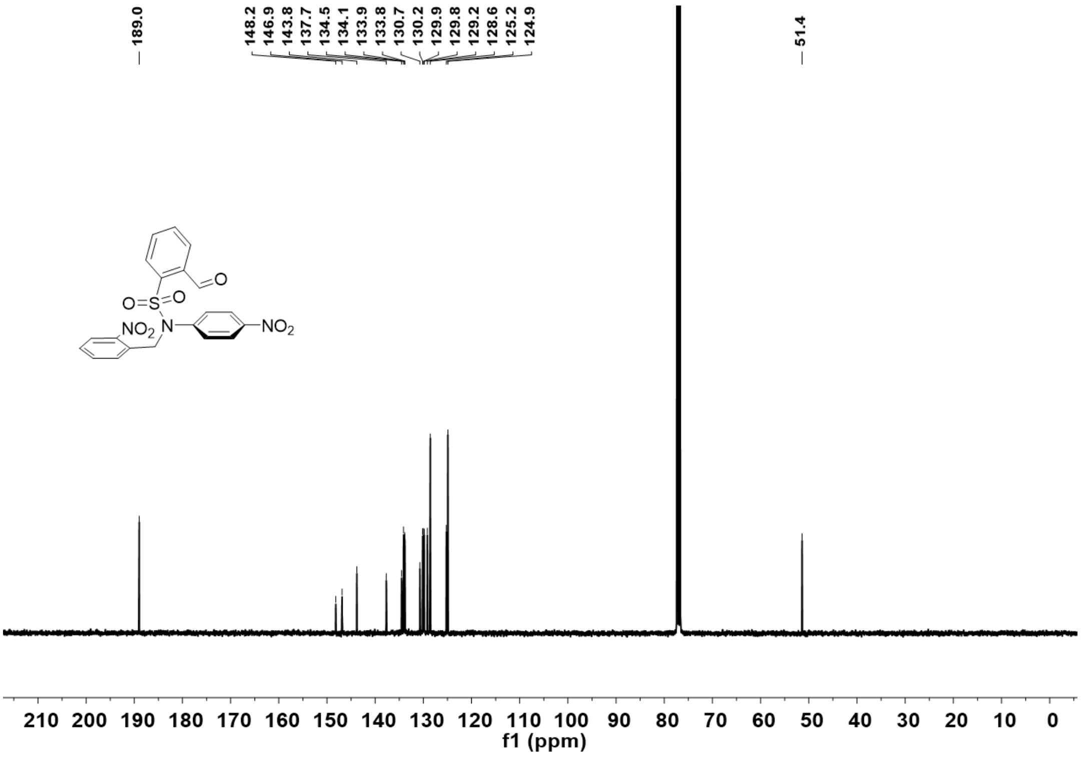

Figure S22. ^{13}C NMR spectrum of **6f** in $CDCl_3$.

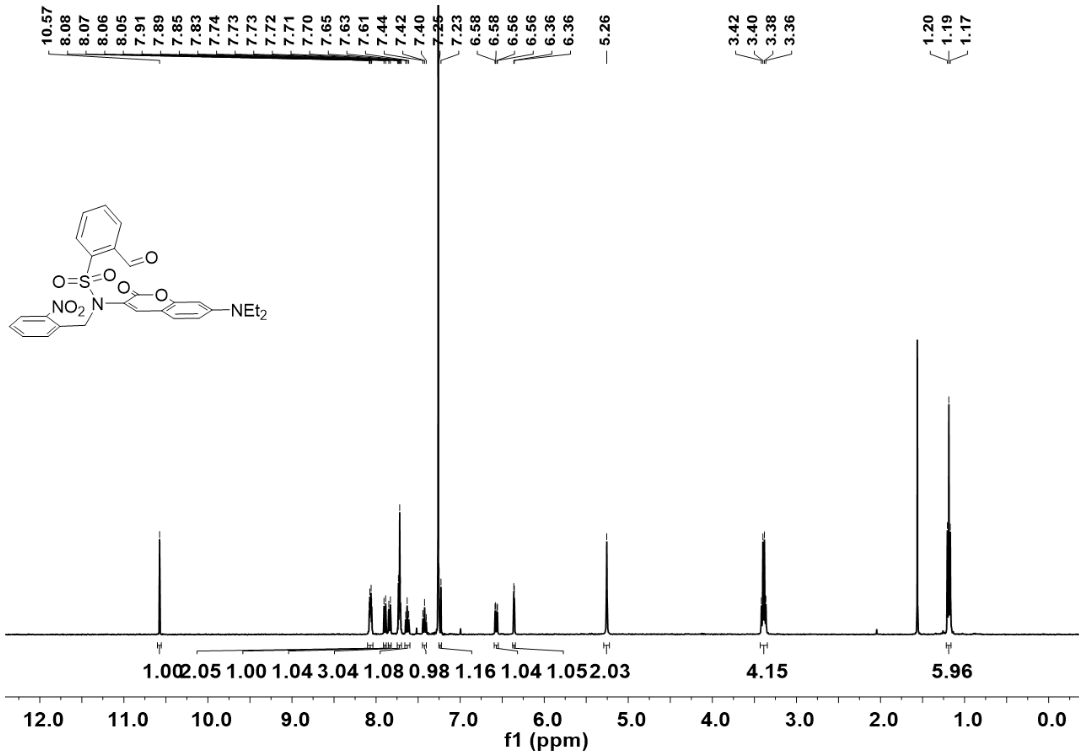

Figure S23. ^{1}H NMR spectrum of **6h** in $CDCl_3$.

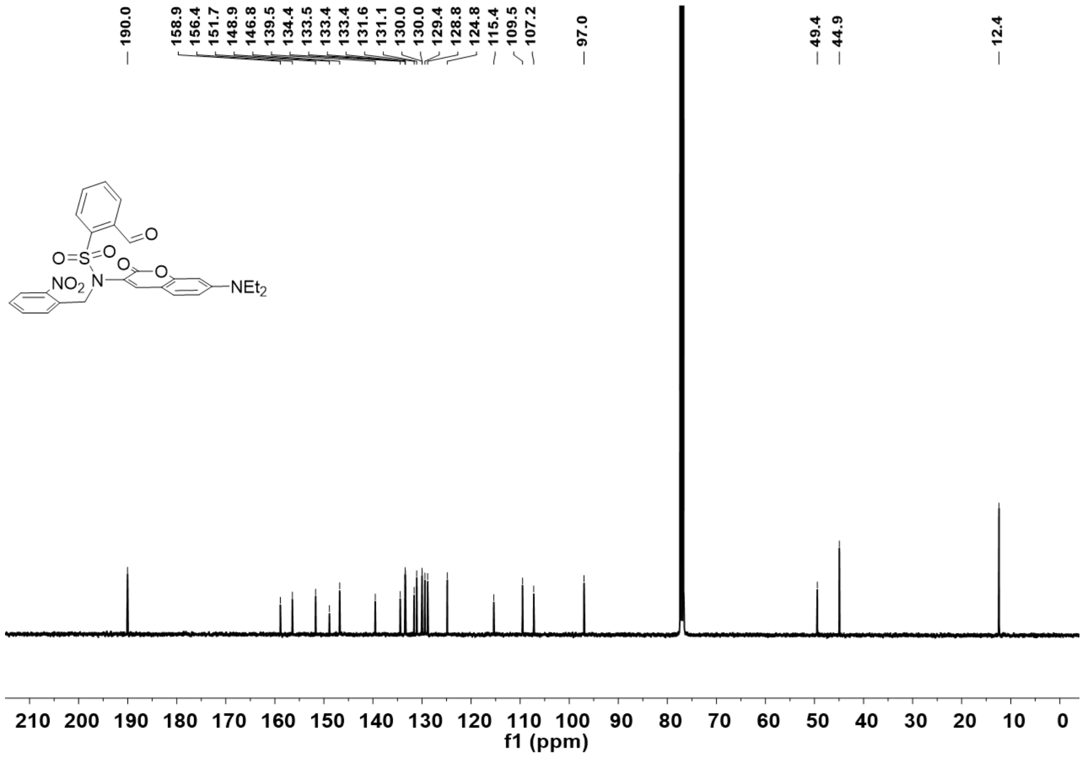

Figure S24. ^{13}C NMR spectrum of **6h** in $CDCl_3$.

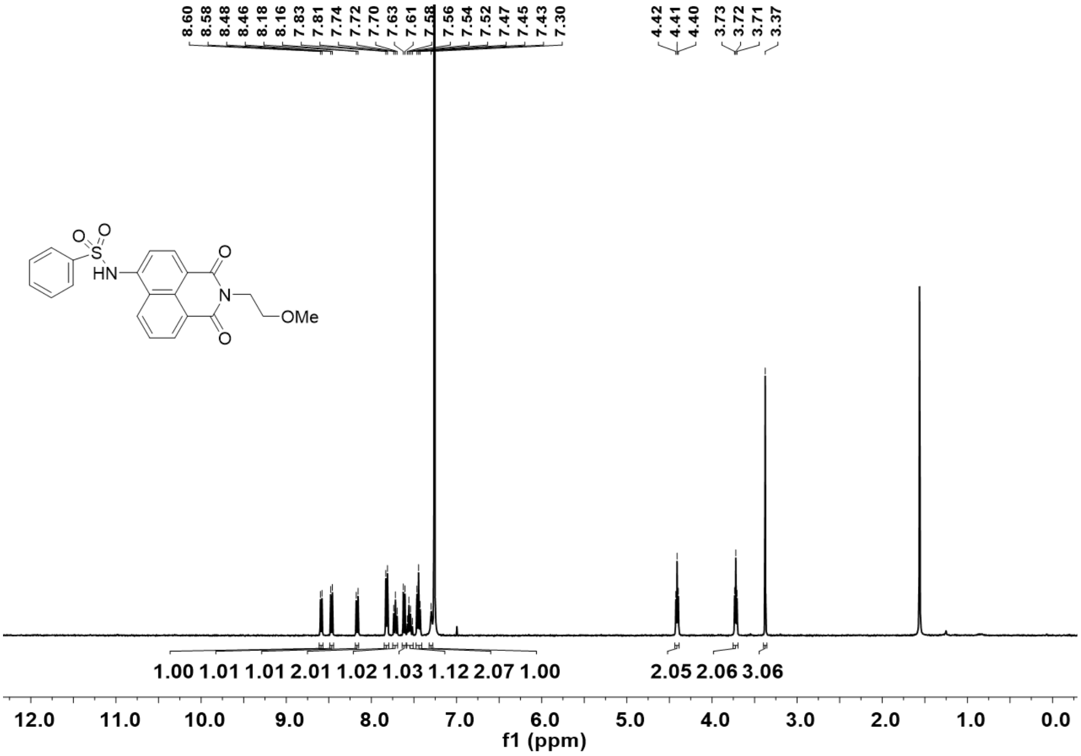

Figure S25. ^{1}H NMR spectrum of **7i** in $CDCl_3$.

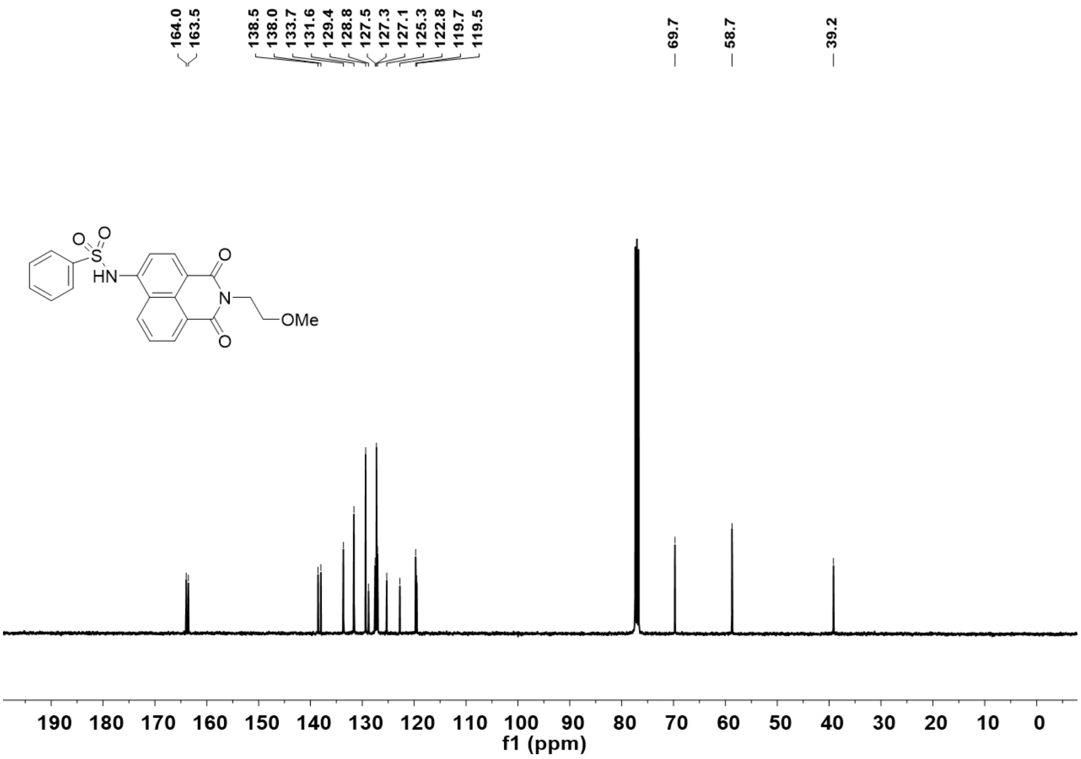

Figure S26. ^{13}C NMR spectrum of **7i** in $CDCl_3$.

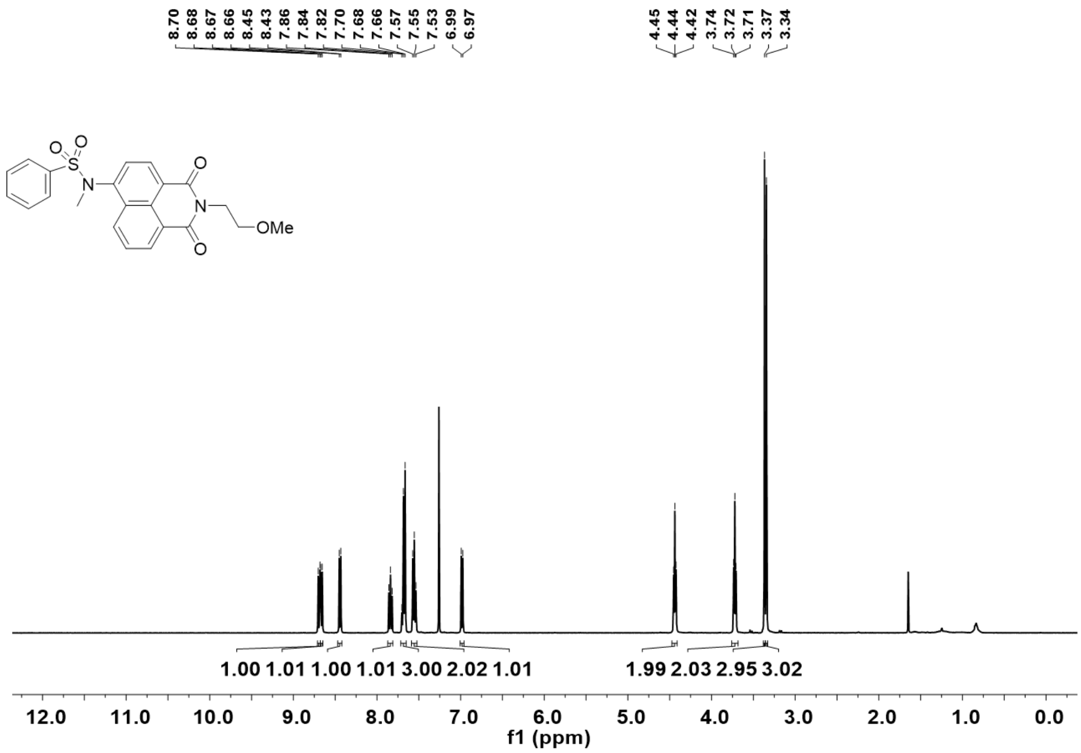

Figure S27. ^{1}H NMR spectrum of **8i** in $CDCl_3$.

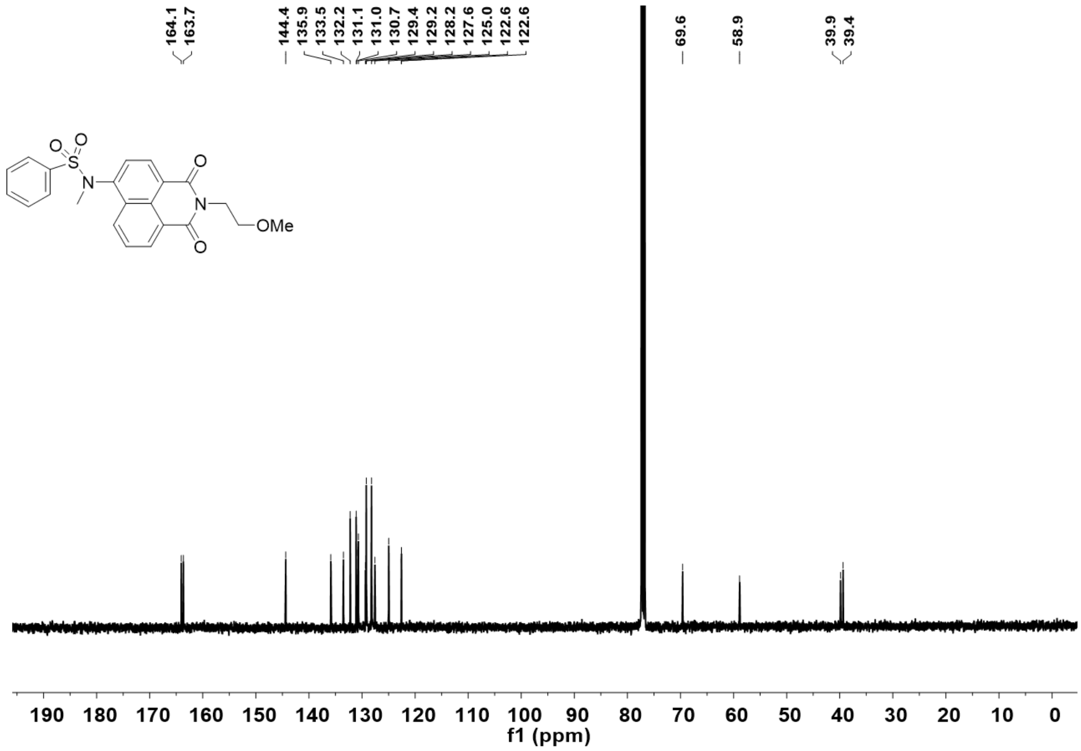

Figure S28. ^{13}C NMR spectrum of **8i** in $CDCl_3$.

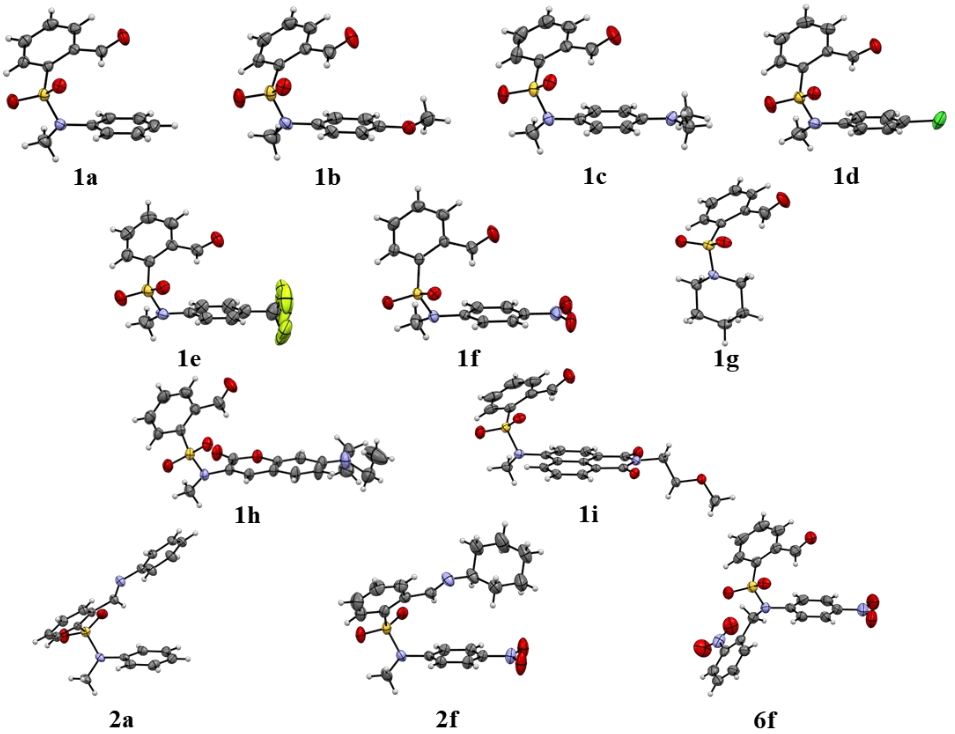

Figure S29. Crystal structures of **1a-1i**, **2a**, **2f**, and **6f**. The thermal ellipsoids were scaled to the 30% probability level.

Table S1. Summary of crystallographic structural parameters of representative aldehydes and imines.[a]

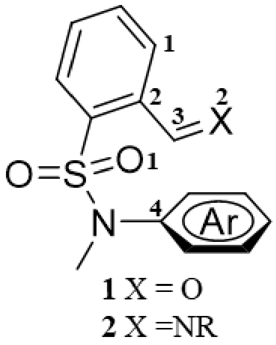

	d_1 (Å)	d_2 (Å)	d_3 (Å)	D (Å)	θ_1 (°)	θ_2 (°)
**1a**	3.341	2.344	1.196	3.215	-4.55	30.36
**1b**	3.352	2.299	1.189	3.302	-1.72	34.81
**1c**	3.258	2.304	1.192	3.145	-1.67	30.12
**1d**	3.248	2.299	1.208	3.219	-16.24	25.88
**1e**	3.269	2.299	1.211	3.253	-16.03	27.20
**1f**	3.349	2.316	1.170	3.320	-11.77	38.56
**1g**	--	2.210	1.171	--	--	--
**1h**	3.666	2.190	1.169	3.538	5.71	44.32
**1i**	3.538	2.283	1.208	3.369	-2.46	36.82
**2a**	3.711	2.343	1.239	3.639	-27.18	69.32
**2f**	3.372	2.316	1.249	3.370	-0.79	49.98

[a] d_1 = C3 ··C4; d_2 = O1 ··C3; d_3 = C3 ··X2; θ_1= C1-C2-C3-X2; D is the shortest distance between C3 and aryl ring plane. θ_2 is the dihedral angle between the planes of CO/CN group and arene.

Table S2. Summary of crystallographic data.

**Compound**	**1a**	**1b**	**1c**	**1d**
Formula	$C_{14}H_{13}NO_3S$	$C_{15}H_{15}NO_4S$	$C_{16}H_{18}N_2O_3S$	$C_{14}H_{12}ClNO_3S$
Formula weight	275.31	305.34	318.38	309.76
T/K	293(2)	293(2)	293(2)	293(2)
Crystallization solvent	acetonitrile	acetonitrile	acetonitrile	acetonitrile
Color	colorless	white	yellow	colorless
Crystal system	monoclinic	monoclinic	monoclinic	monoclinic
Space group	$P2_1/n$	$P2_1/c$	$P2_1/c$	$P2_1/c$
a / Å	9.5117(5)	14.1451(15)	6.53336(18)	12.3811(3)
b / Å	11.2079(5)	8.0558(4)	23.3765(5)	13.2518(4)
c / Å	12.8350(6)	14.2675(15)	10.4889(3)	8.8737(3)
α / °	90	90	90	90
β / °	106.032(5)	115.163(4)	96.741(2)	97.742(3)
γ / °	90	90	90	90
V/ Å^3	1315.07(11)	1471.5(2)	1590.86(7)	1442.65(7)
Z	4	4	4	4
D_x / g cm^{-3}	1.391	1.378	1.329	1.426
μ / mm^{-1}	2.227	0.235	1.931	3.760
F (000)	576	640	672	640
θ range / °	5.17 to 76.88	2.98 to 27.49	3.78 to 76.55	3.60 to 76.88
GOF on F^2	1.076	1.116	1.074	1.258
R_1 [$I > 2\sigma(I)$]	0.0362	0.0443	0.0588	0.0950
wR_2 (all data)	0.0951	0.1419	0.1671	0.2893

**Compound**	**1e**	**1f**	**1g**	**1h**
Formula	$C_{15}H_{12}F_3NO_3S$	$C_{42}H_{36}N_6O_{15}S_3$	$C_{12}H_{15}NO_3S$	$C_{21}H_{22}N_2O_5S$
Formula weight	343.32	960.95	253.31	414.46
T/*K*	293(2)	293(2)	293(2)	293(2)
Crystallization solvent	acetonitrile	acetonitrile	acetonitrile	acetonitrile
Color	colorless	yellow	colorless	colorless
Crystal system	monoclinic	monoclinic	monoclinic	monoclinic
Space group	$P2_1/c$	Pc	$P2_1/n$	$P2_1/c$
a / Å	12.7880(8)	16.3097(7)	10.0590(3)	15.5534(5)
b / Å	13.8067(7)	11.7465(3)	8.2626(3)	8.3572(3)
c / Å	8.8344(6)	11.6340(4)	15.2156(5)	16.2716(6)
α / °	90	90	90	90
β / °	99.869(6)	106.181(4)	101.154(3)	97.664(3)
γ / °	90	90	90	90
V/ Å^3	1536.73(16)	2140.57(14)	1240.73(7)	2096.14(13)
Z	4	2	4	4
D_x / g cm^{-3}	1.484	1.491	1.356	1.313
μ / mm^{-1}	2.318	2.271	2.303	1.668
F (000)	704	996	536	872
θ range / °	3.51 to 78.56	2.82 to 76.71	4.87 to 76.59	2.87 to 76.49
GOF on F^2	1.331	1.062	1.068	1.135
R_1 [$I > 2\sigma(I)$]	0.1152	0.0477	0.0451	0.0785
wR_2 (all data)	0.3470	0.1433	0.1344	0.2602

**Compound**	**1i**	**2a**	**2f**	**6f**
Formula	$C_{23}H_{19}N_2O_6S$	$C_{20}H_{18}N_2O_2S$	$C_{36}H_{41}N_9O_9S_2$	$C_{20}H_{15}N_3O_7S$
Formula weight	451.46	350.42	807.90	441.41
T/K	293(2)	293(2)	293(2)	293(2)
Crystallization solvent	acetonitrile	acetonitrile	acetonitrile	acetonitrile
Color	yellow	white	yellow	yellow
Crystal system	orthorhombic	monoclinic	monoclinic	monoclinic
Space group	*Pbca*	$P2_1$	$P2_1/c$	$P2_1/n$
a / Å	16.89290(10)	5.95041(12)	16.3618(3)	7.4052(6)
b / Å	13.74290(10)	13.2602(3)	15.0785(2)	24.9995(12)
c / Å	18.3234(2)	11.1645(2)	17.6103(2)	11.2814(8)
α / °	90	90	90	90
β / °	90	93.6083(19)	104.624(2)	107.855(8)
γ / °	90	90	90	90
V/ Å^3	4253.91(6)	879.18(3)	4203.91(11)	1987.9(2)
Z	8	2	4	4
D_x / g cm^{-3}	1.410	1.324	1.276	1.475
μ / mm^{-1}	1.734	1.759	1.665	1.896
F (000)	1880	368	1696	912
θ range / °	4.80 to 76.64	3.97 to 76.74	2.79 to 76.98	3.54 to 66.59
GOF on F^2	1.079	1.039	1.068	1.086
R_1 [$I > 2\sigma(I)$]	0.0416	0.0402	0.0447	0.1219
wR_2 (all data)	0.1331	0.1062	0.1366	0.3268

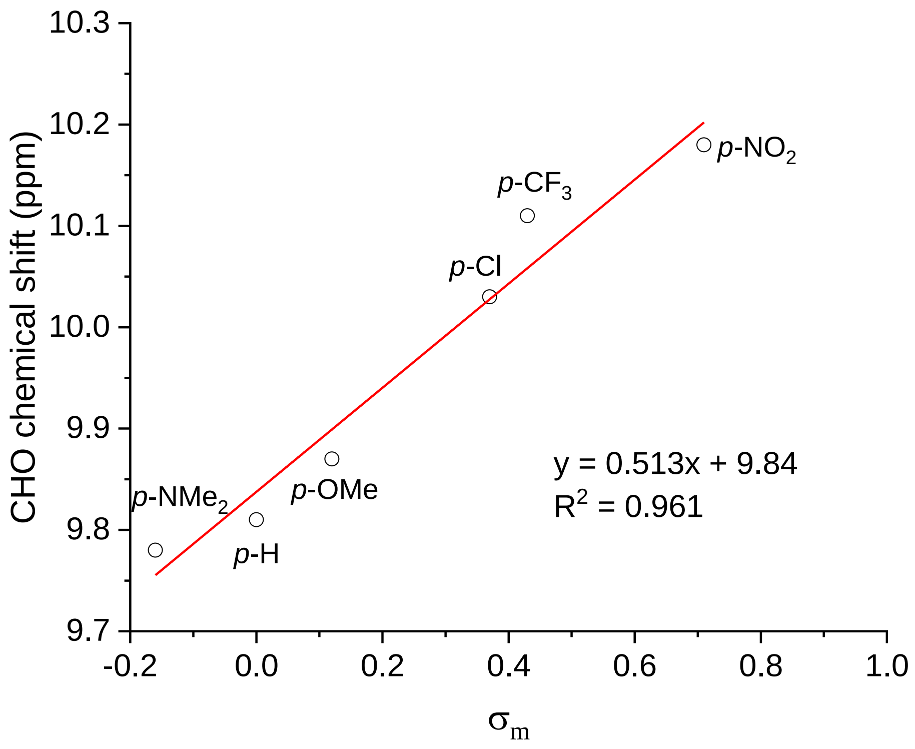

Figure S30. Hammett plot of ^{1}H NMR chemical shift of CHO for **1a-1f** versus σ_m.

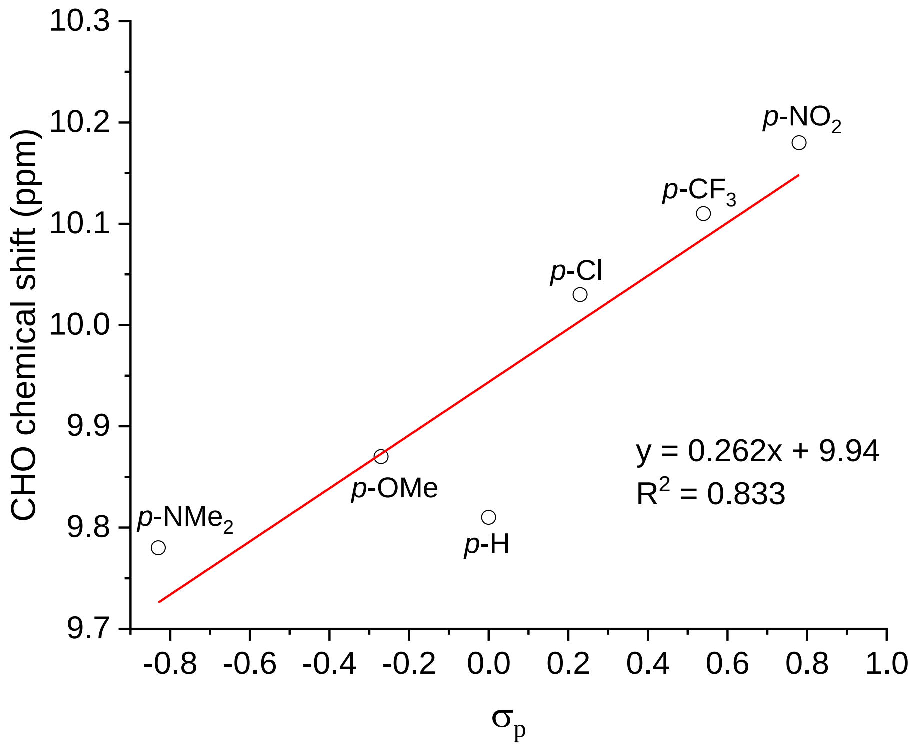

Figure S31. Hammett plot of ^{1}H NMR chemical shift of CHO for **1a-1f** versus σ_p.

3. DFT Calculations

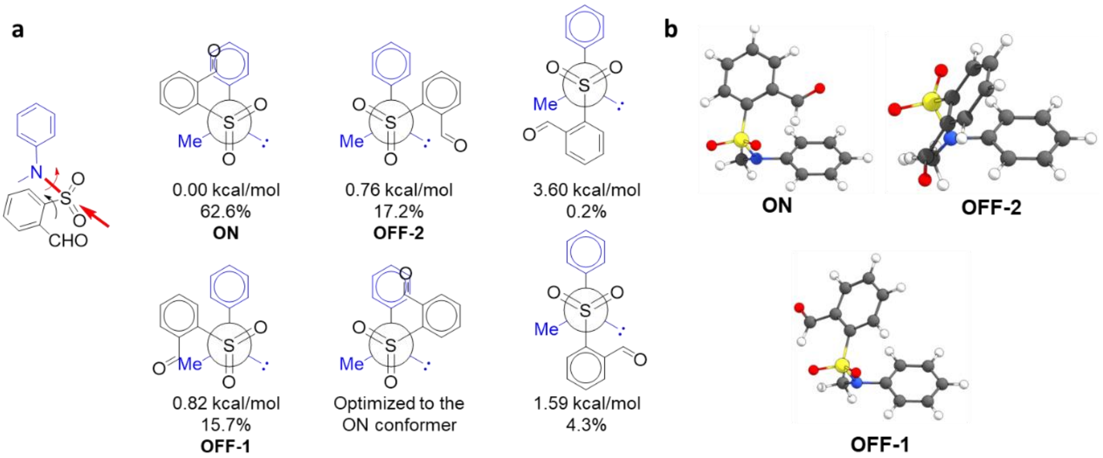

Figure S32. (a) Generation of conformers of **1a** by the rotation of S-N and S-C bonds, with their relative Gibbs free energy and conformational population listed. (b) Calculated structures of ON, OFF-1, OFF-2 conformers for **1a**.

Table S3. Relative Gibbs free energy and conformational population for **1** and **2**.

	ΔG (kcal/mol)	Percentage		ΔG (kcal/mol)	Percentage
**1a**-ON	0.00	65.6%	**2a**-ON	0.00	55.1%
**1a**-OFF-1	0.81	16.7%	**2a**-OFF-1	0.67	17.8%
**1a**-OFF-2	0.78	17.6%	**2a**-OFF-2	0.42	27.1%
**1b**-ON	0.00	72.5%	**2b**-ON	0.00	55.5%
**1b**-OFF-1	1.01	13.2%	**2b**-OFF-1	0.59	20.4%
**1b**-OFF-2	0.96	14.2%	**2b**-OFF-2	0.49	24.1%
**1c**-ON	0.00	66.9%	**2c**-ON	0.00	68.9%
**1c**-OFF-1	1.21	8.7%	**2c**-OFF-1	0.82	17.2%
**1c**-OFF-2	0.60	24.3%	**2c**-OFF-2	0.95	13.8%
**1d**-ON	0.00	74.1%	**2d**-ON	0.00	59.5%
**1d**-OFF-1	1.15	10.6%	**2d**-OFF-1	0.52	24.9%
**1d**-OFF-2	0.94	15.3%	**2d**-OFF-2	0.79	15.7%
**1e**-ON	0.00	60.3%	**2e**-ON	0.00	60.4%
**1e**-OFF-1	0.73	17.7%	**2e**-OFF-1	0.97	11.7%
**1e**-OFF-2	0.60	22.0%	**2e**-OFF-2	0.46	27.8%
**1f**-ON	0.00	70.5%	**2f**-ON	0.00	79.2%
**1f**-OFF-1	0.80	18.2%	**2f**-OFF-1	1.22	10.0%
**1f**-OFF-2	1.08	11.4%	**2f**-OFF-2	1.18	10.7%

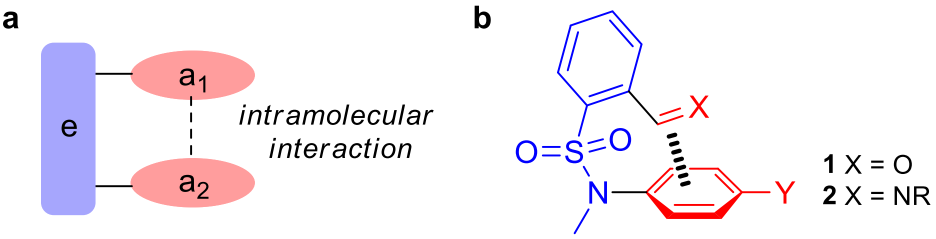

Figure S33. (a) The schematic division of GKS-EDA including the intramolecular interacting fragments {a1, a2} and the environmental fragment {e}. (b) The division for our system with the intramolecular interacting fragments {a1: C=X group, a2: para-substituted phenyl group} marked in red and the environmental fragment {e: the atoms except from a1 and a2 fragments} marked in blue, respectively.

Table S4. GKS-EDA analysis for the ON conformer of **1** and **2** with the structural fragments highlighted in red.

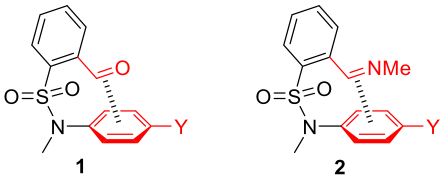

	ΔE^{ele}	ΔE^{ex+rep}	ΔE^{pol}	ΔE^{disp}	ΔE^{corr}	ΔE^{TOT}
**1a**	-3.09	6.02	-0.99	-3.07	-0.42	-1.54
**1b**	-3.19	4.72	-0.89	-2.86	-0.03	-2.25
**1c**	-3.66	4.86	-1.12	-3.03	0.06	-2.89
**1d**	-2.18	4.57	-0.67	-2.85	-0.29	-1.41
**1e**	-1.88	4.62	-0.68	-2.88	-0.39	-1.20
**1f**	-1.59	4.69	-0.79	-2.89	-0.46	-1.05
**2a**	-2.95	7.15	-0.69	-4.70	-0.18	-1.36
**2b**	-3.15	6.20	-0.78	-5.15	0.21	-2.67
**2c**	-3.53	7.67	-1.04	-6.10	0.20	-2.80
**2d**	-2.74	6.05	-0.50	-4.96	-0.16	-2.32
**2e**	-2.35	5.56	-0.29	-4.78	-0.11	-1.98
**2f**	-2.22	5.65	-0.41	-4.84	-0.28	-2.11

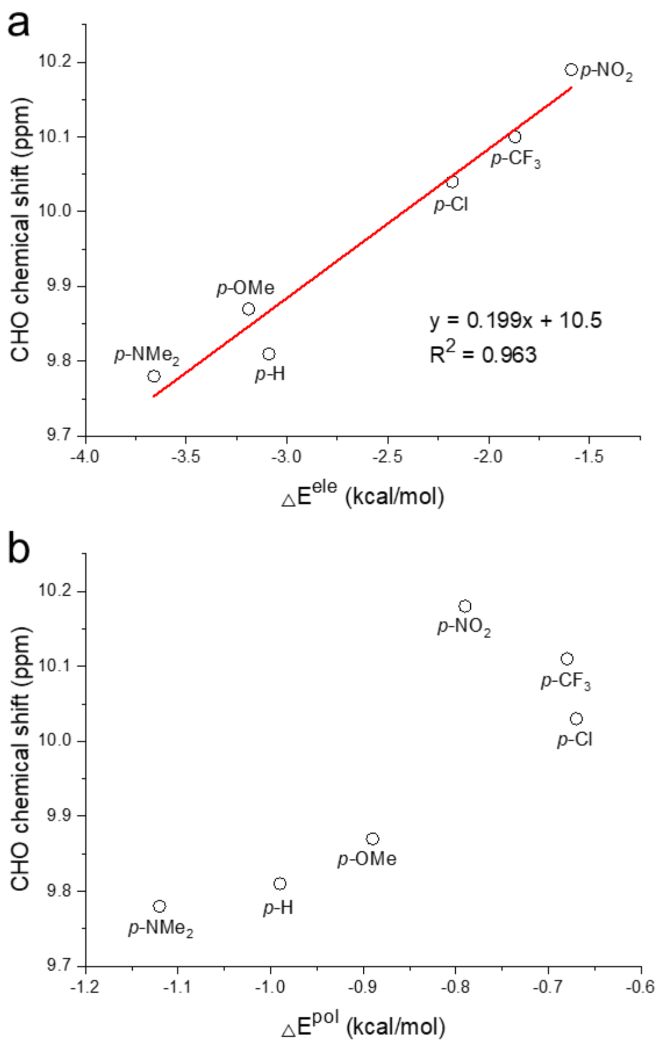

Figure S34. (a) Correlation of ^{1}H NMR chemical shift of CHO for **1a-1f** with ΔE^{ele}. (b) Correlation of ^{1}H NMR chemical shift of CHO for **1a-1f** with ΔE^{pol}.

Table S5. The sterimol parameters of aldehyde/imine fragment for **1** and **2**.

	L	B_1	B_5		L	B_1	B_5
**1a**	6.076	1.776	5.988	**2a**	5.959	1.779	6.020
**1b**	6.861	1.772	6.391	**2b**	6.312	1.772	6.611
**1c**	7.929	1.768	6.350	**2c**	7.714	1.775	6.670
**1d**	6.956	1.772	5.969	**2d**	6.649	1.776	6.000
**1e**	7.353	1.775	6.290	**2e**	7.214	1.778	6.684
**1f**	7.391	1.796	5.958	**2f**	7.173	1.786	6.279

Table S6. The electrostatic energy difference ($\Delta\Delta E^{ele}$)[a] between compounds **1** and **2**, the difference of the sterimol parameters (ΔB_1 and ΔB_5, in 10^{-10} m)[b] between compounds **1** and **2**, as well as the predicted and measured ΔG[c] of imine exchange. The units of $\Delta\Delta E^{ele}$ and ΔG are kcal/mol. The predicted ΔG value was calculated based on multivariate linear correlation of measured ΔG versus $\Delta\Delta E^{ele}$, ΔB_1, and ΔB_5.

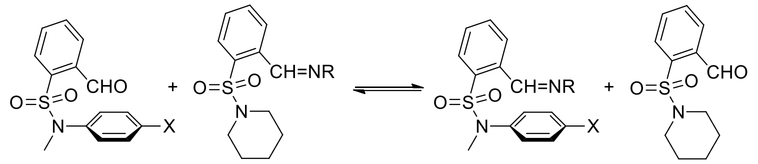

X	$\Delta\Delta E^{ele}$	ΔB_1	ΔB_5	Predicted ΔG	Measured ΔG
p-NO_2	-0.622	-0.010	0.321	-0.113	-0.109
p-CF_3	-0.472	0.003	0.394	-0.298	-0.285
p-Cl	-0.566	0.004	0.031	-0.229	-0.241
p-H	0.132	0.003	0.032	0.305	0.329
p-OMe	0.038	0.000	0.220	0.270	0.245
p-NMe_2	0.126	0.007	0.320	0.135	0.131

[a] The electrostatic energy difference was calculated by the electrostatic energy of **1** subtracting that of **2** from Table S4.

[b] The difference of the sterimol parameters B_1 and B_5 was calculated by the corresponding values of **1** subtracting that of **2** from Table S5.

[c] Measured $\Delta G = 0.787*\Delta\Delta E^{ele} - 20.5*\Delta B_1 - 0.383*\Delta B_5 + 0.299$

The quality of multivariate linear regression of measured ΔG versus $\Delta\Delta E^{ele}$, ΔB_1, and ΔB_5 was supported by a good linear relationship (Measured ΔG = 1.00*(Predicted ΔG) - $1.71*10^{-4}$, R^2 = 0.999, as shown in Figure 4D) between the experimental and the predicted data. By using a standardization method of regression coefficient, the distribution of $\Delta\Delta E^{ele}$, ΔB_1, and ΔB_5 was determined by their corresponding values of standard regression coefficient of 1.10, -0.467, and -0.230, respectively.

Table S7. NBO stabilization energies of arene-aldehyde/imine interactions for **1** and **2**.

	NBO (kcal/mol)		NBO (kcal/mol)
**1a**	1.07	**2a**	0.60
**1b**	1.25	**2b**	0.68
**1c**	1.33	**2c**	0.80
**1d**	0.92	**2d**	0.50
**1e**	1.02	**2e**	0.55
**1f**	0.80	**2f**	0.40

Table S8. Calculated dihedral angle of the aldehyde/imine fragment with the connected aromatic ring.

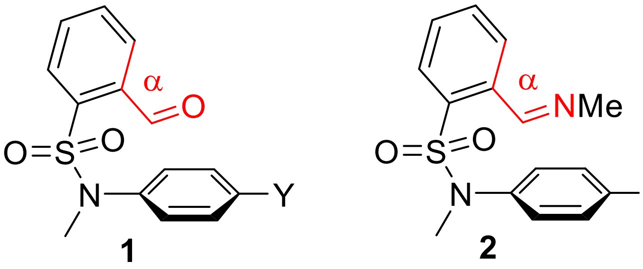

	α (°)		α (°)
**1a**	5.998	**2a**	15.529
**1b**	7.664	**2b**	17.213
**1c**	5.998	**2c**	5.998
**1d**	11.168	**2d**	18.510
**1e**	12.613	**2e**	17.327
**1f**	16.261	**2f**	20.899

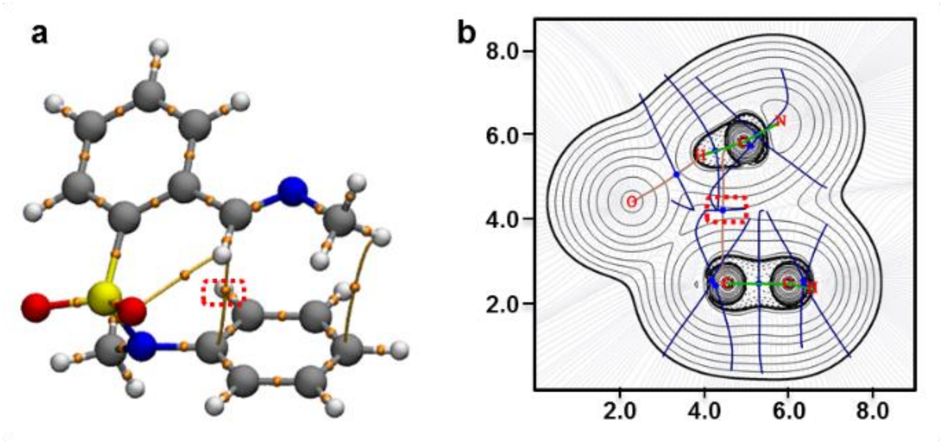

Figure S35. (a) BCP of (3, -1) highlighted with red dot frame and the related path of aromatic-imine interaction for **2a**. (b) Laplacian of electron density for the aromatic-imine interaction highlighted with red dot frame.

Table S9. Topological analysis of the electron density distribution for the BCP (3, -1) of arene-aldehyde/imine interactions within **1** and **2**.

	ρ(r)	$\nabla^2\rho(r)$	H(r)	V(r)	G(r)	-G/V	$\lambda_1, \lambda_2, \lambda_3$
**1a**	8.47E-03	2.67E-02	1.03E-03	-4.62E-03	5.65E-03	1.22	-5.71E-03, -2.17E-03, 3.46E-02
**1b**	8.68E-03	2.75E-02	1.06E-03	-4.76E-03	5.82E-03	1.22	-5.94E-03, -1.82E-03, 3.53E-02
**1c**	9.04E-03	2.82E-02	1.05E-03	-4.95E-03	6.00E-03	1.21	-6.23E-03, -2.02E-03, 3.64E-02
**1d**	8.14E-03	2.59E-02	1.05E-03	-4.38E-03	5.43E-03	1.24	-5.19E-03, -1.74E-03, 3.28E-02
**1e**	8.06E-03	2.56E-02	1.04E-03	-4.30E-03	5.35E-03	1.24	-5.02E-03, -1.83E-03, 3.24E-02
**1f**	7.90E-03	2.51E-02	1.05E-03	-4.17E-03	5.22E-03	1.25	-4.72E-03, -1.76E-03, 3.15E-02
**2a**	6.89E-03	2.28E-02	1.05E-03	-3.60E-03	4.65E-03	1.29	-3.84E-03, -6.08E-04, 2.72E-02
**2b**	7.30E-03	2.36E-02	1.03E-03	-3.82E-03	4.86E-03	1.27	-3.22E-03, -4.99E-04, 2.73E-02
**2c**	7.62E-03	2.47E-02	1.06E-03	-4.05E-03	5.11E-03	1.26	-4.29E-03, -2.08E-04, 2.92E-02
**2d**	6.90E-03	2.26E-02	1.02E-03	-3.60E-03	4.62E-03	1.28	-3.69E-03, -2.50E-04, 2.65E-02
**2e**	6.70E-03	2.21E-02	1.03E-03	-3.46E-03	4.49E-03	1.30	-3.60E-03, -5.29E-04, 2.62E-02
**2f**	6.73E-03	2.17E-02	1.01E-03	-3.42E-03	4.43E-03	1.29	-3.47E-03, -7.01E-04, 2.59E-02

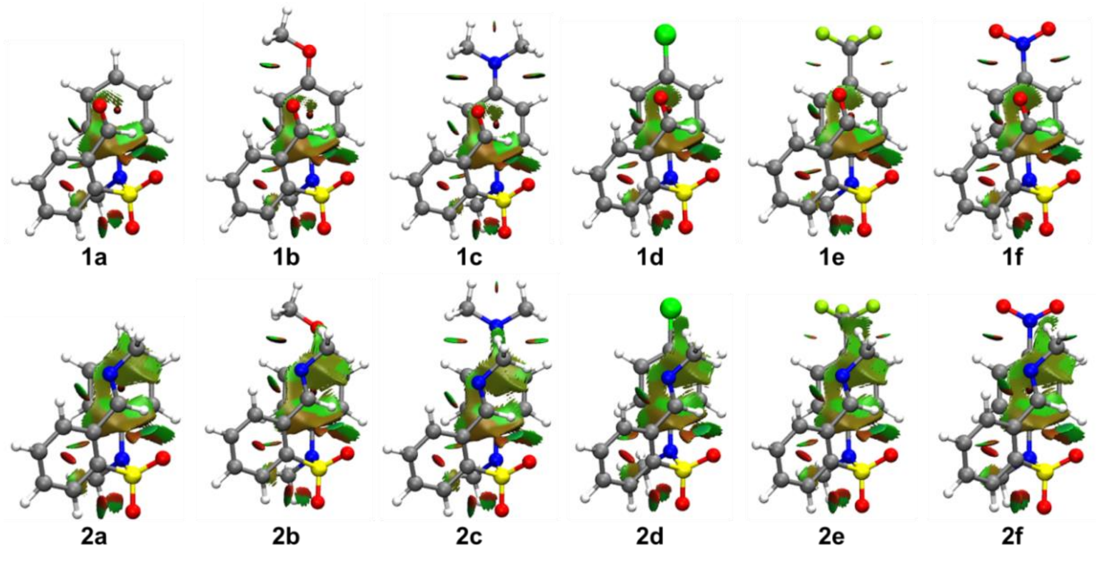

Figure S36. NCI plots for arene-aldehyde/imine interactions for **1** and **2** with different substituents.

4. Dynamic Covalent Reactions

(1) Imine formation

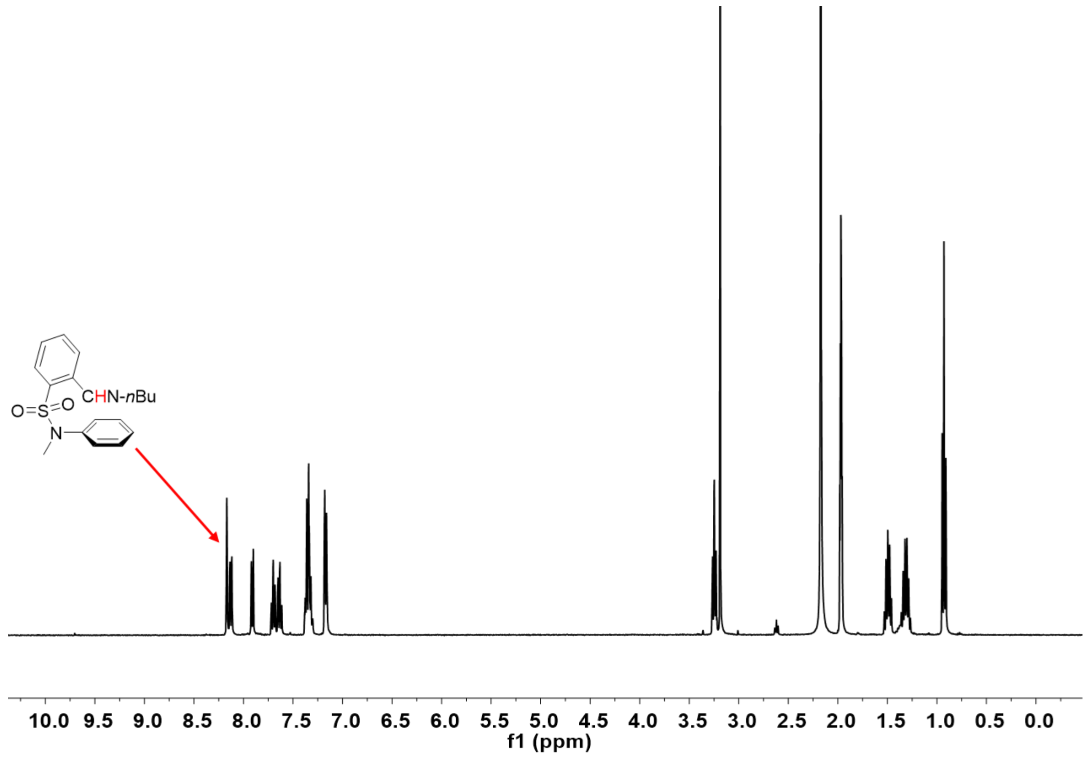

Figure S37. ^{1}H NMR spectrum of the reaction of **1a** and 1-butylamine in CD_3CN.

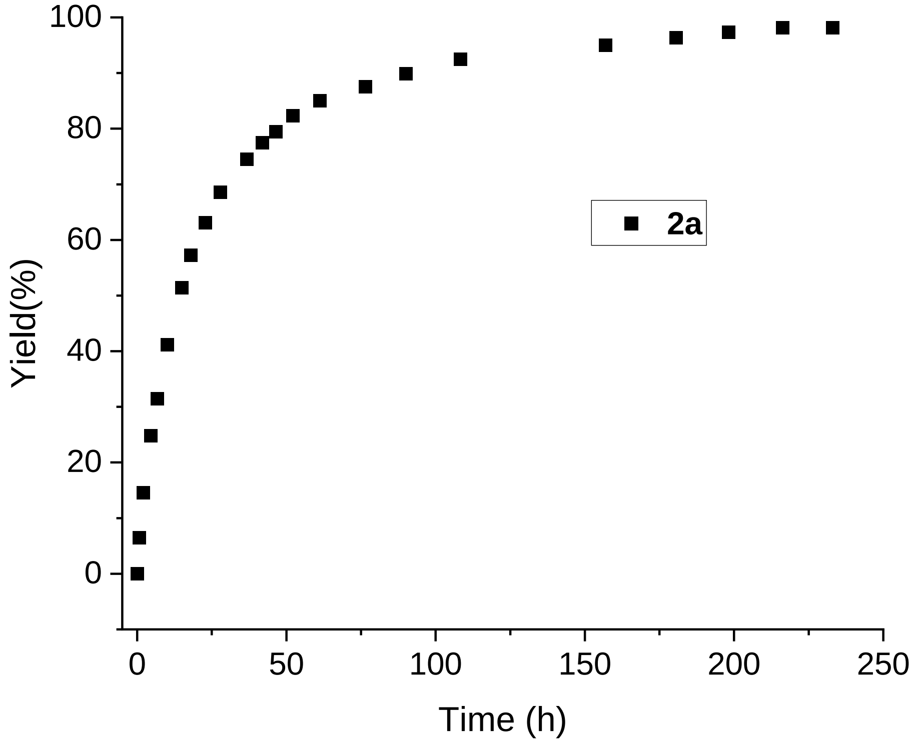

Figure S38. Kinetic profile of the reaction of **1a** and 1-butylamine in CD_3CN.

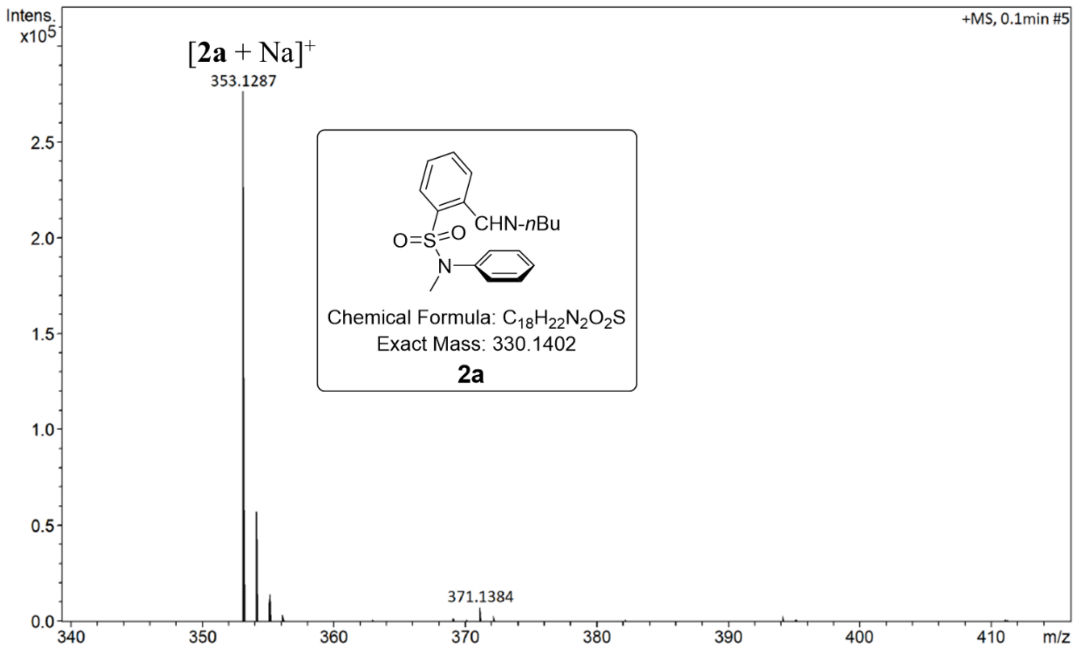

Figure S39. ESI-MS spectrum of the reaction of **1a** and 1-butylamine in CD3CN.

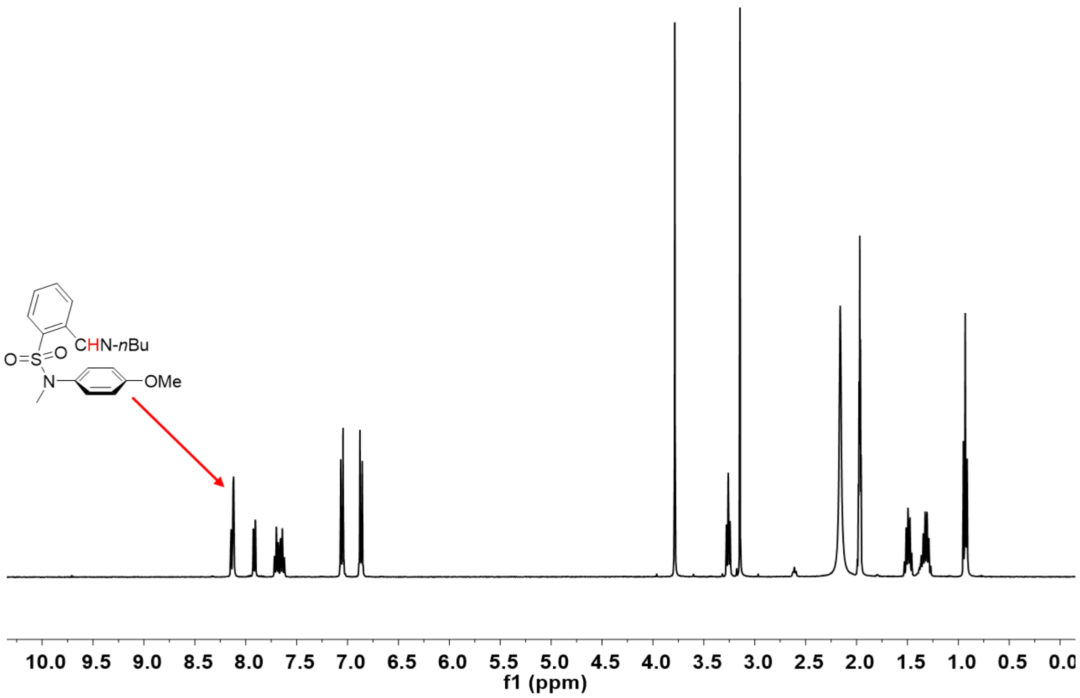

Figure S40. ^{1}H NMR spectrum of the reaction of **1b** and 1-butylamine in CD3CN.

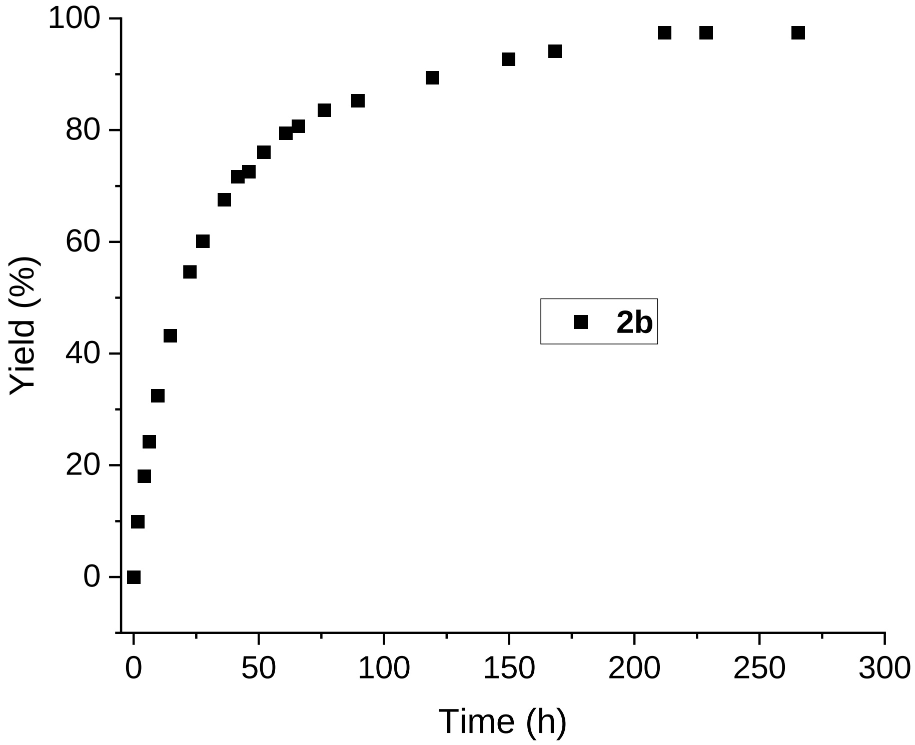

Figure S41. Kinetic profile of the reaction of **1b** and 1-butylamine in CD_3CN.

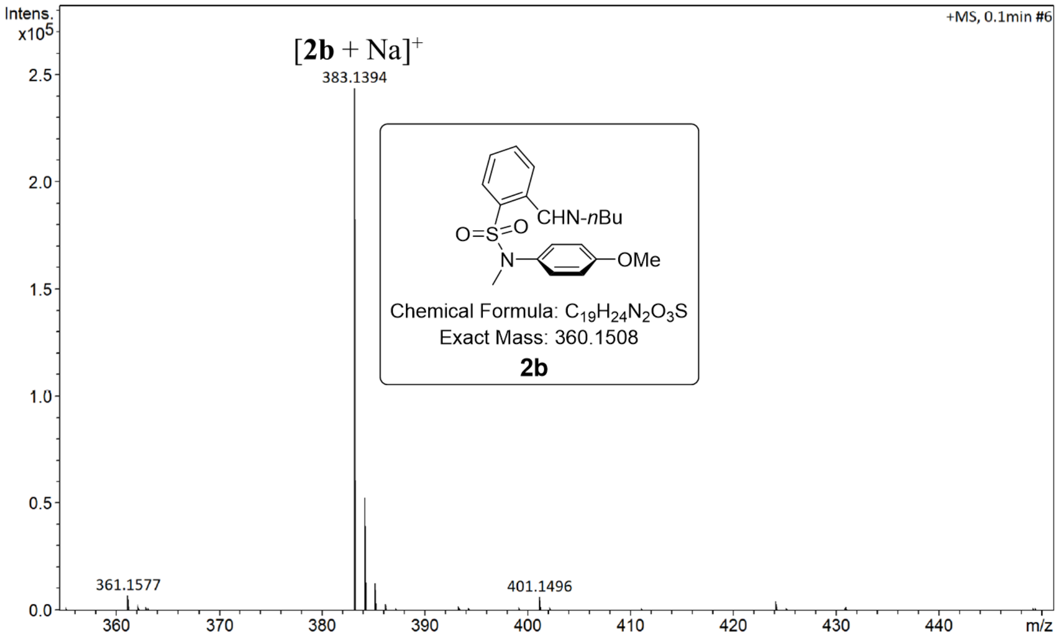

Figure S42. ESI-MS spectrum of the reaction of **1b** and 1-butylamine in CD_3CN.

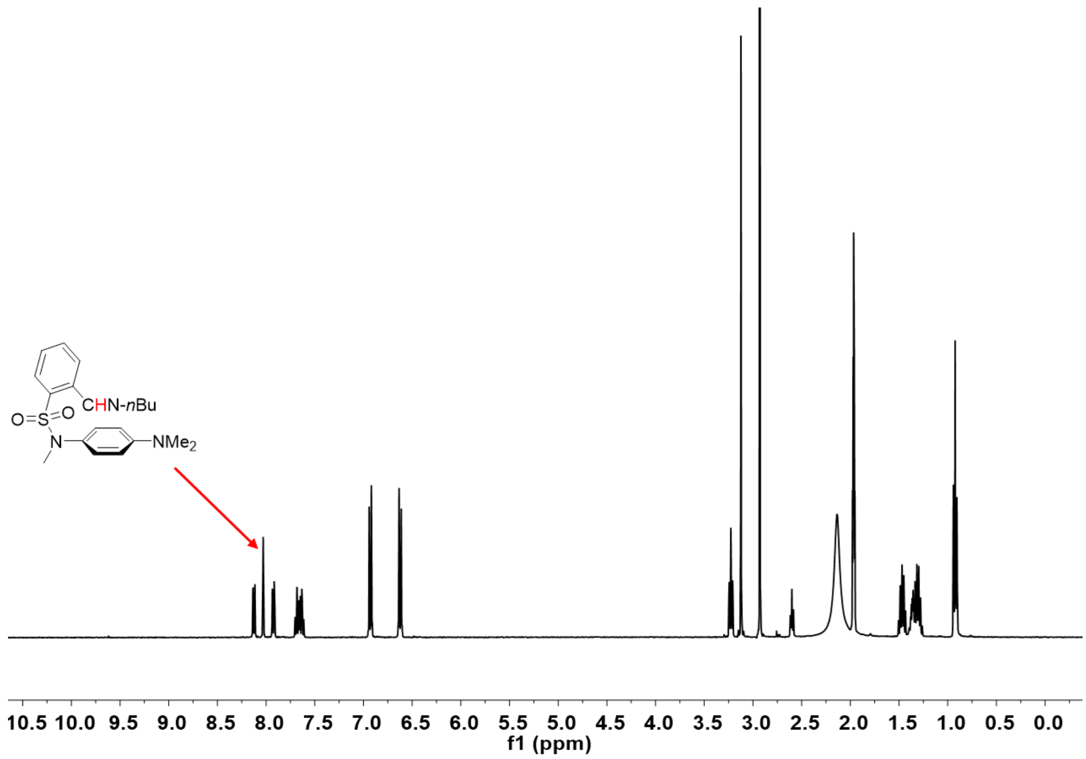

Figure S43. ^{1}H NMR spectrum of the reaction of **1c** and 1-butylamine in CD_3CN.

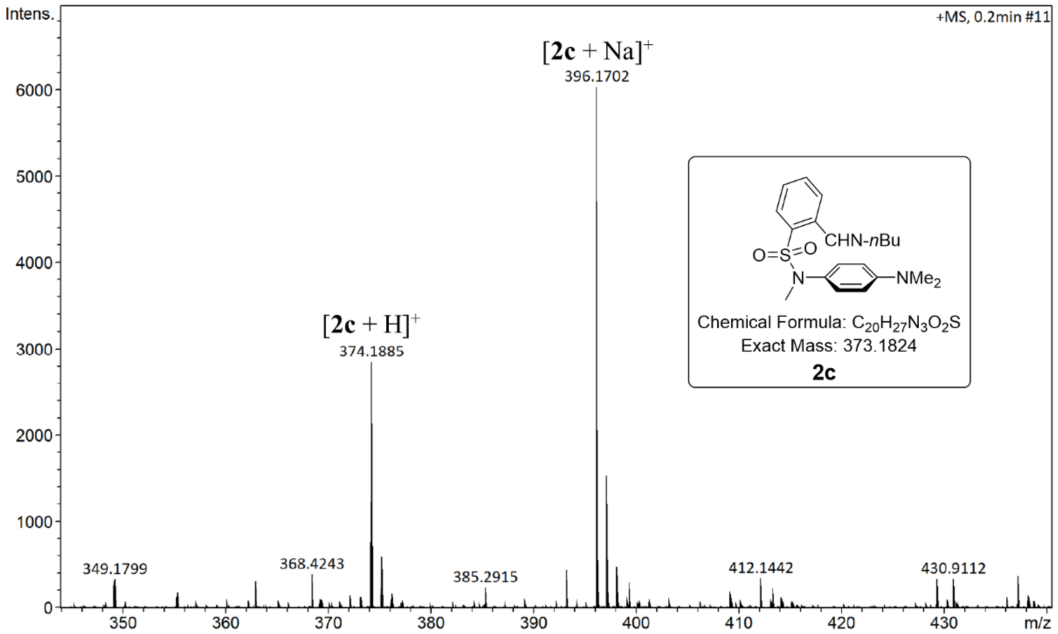

Figure S44. ESI-MS spectrum of the reaction of **1c** and 1-butylamine in CD_3CN.

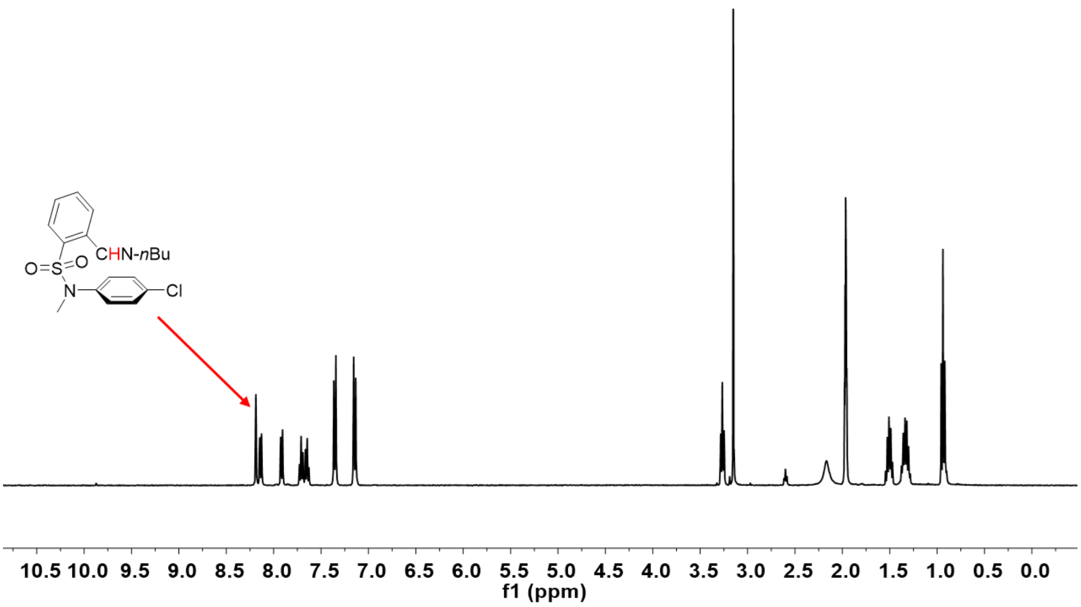

Figure S45. ^{1}H NMR spectrum of the reaction of **1d** and 1-butylamine in CD3CN.

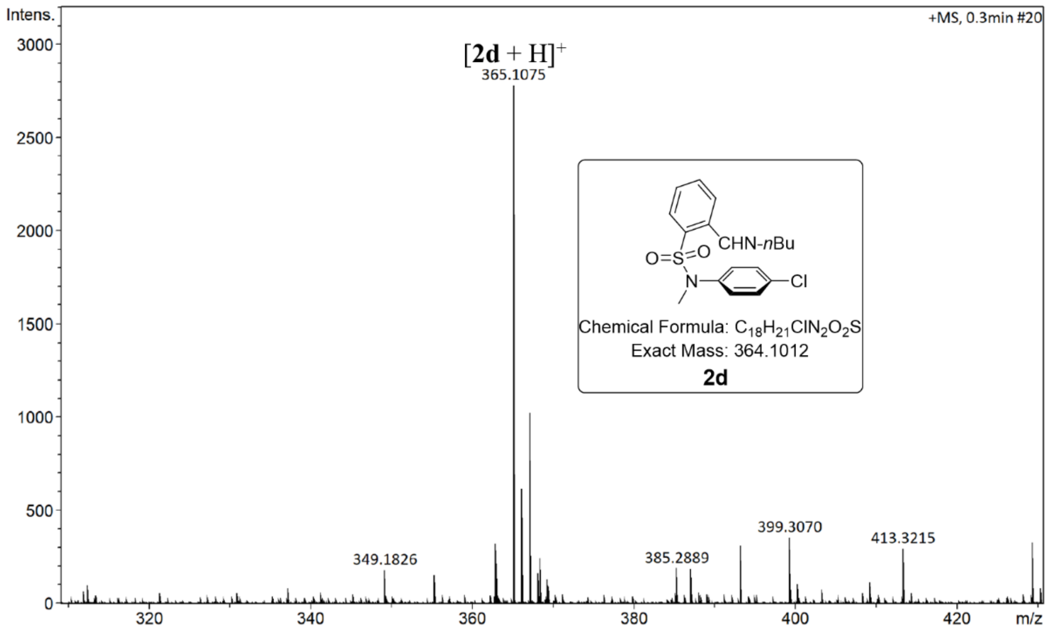

Figure S46. ESI-MS spectrum of the reaction of **1d** and 1-butylamine in CD3CN.

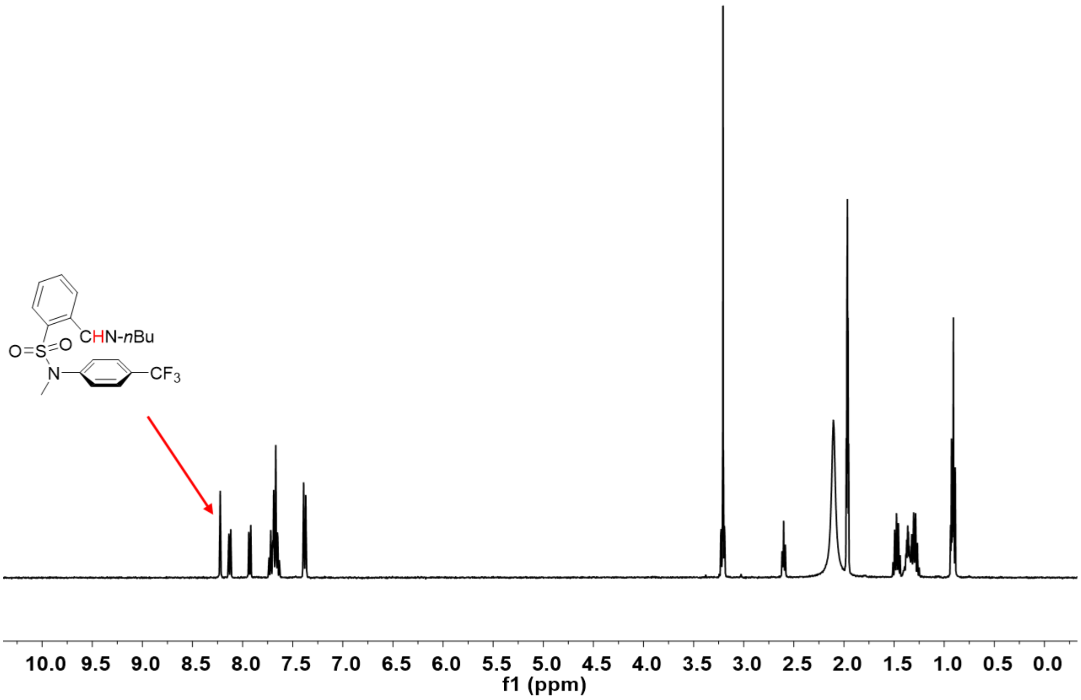

Figure S47. ^{1}H NMR spectrum of the reaction of **1e** and 1-butylamine in CD_3CN.

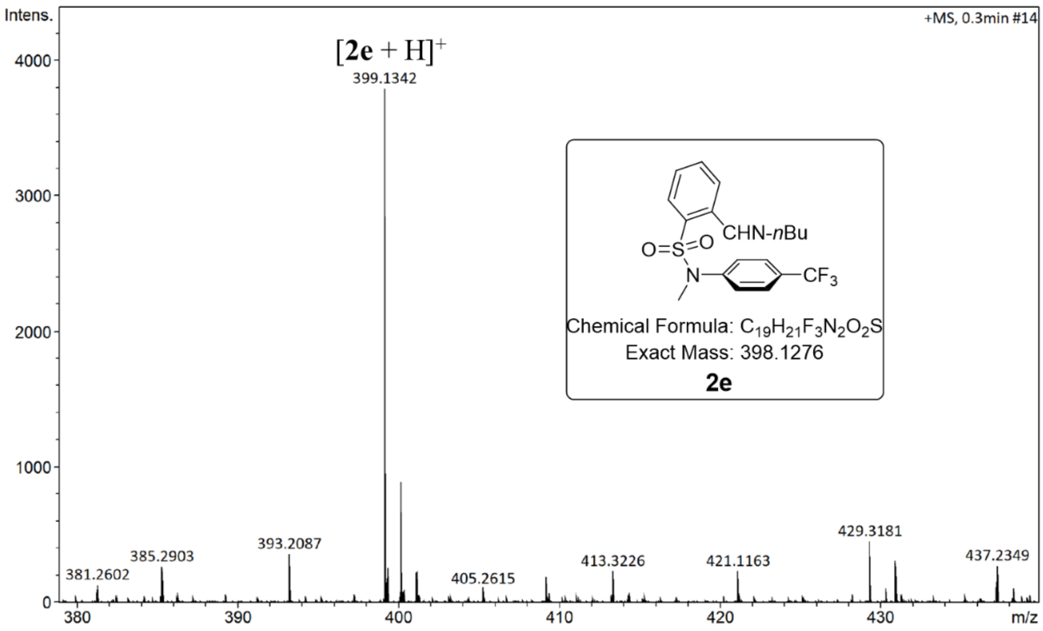

Figure S48. ESI-MS spectrum of the reaction of **1e** and 1-butylamine in CD_3CN.

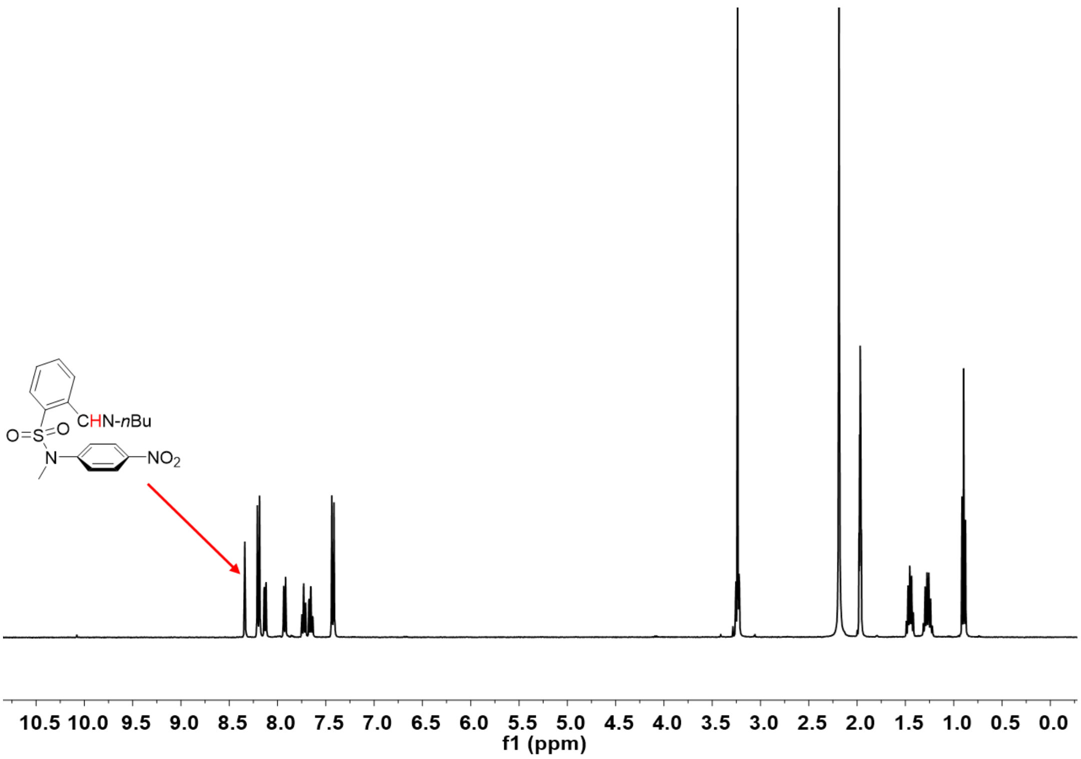

Figure S49. ^{1}H NMR spectrum of the reaction of **1f** and 1-butylamine in CD_3CN.

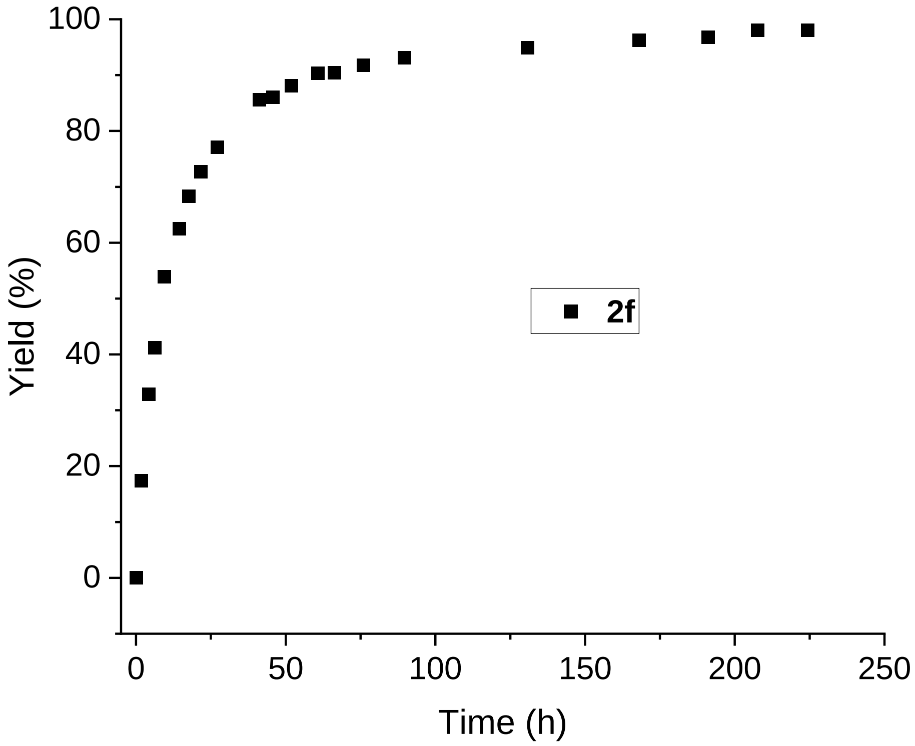

Figure S50. Kinetic profile of the reaction of **1f** and 1-butylamine in CD_3CN.

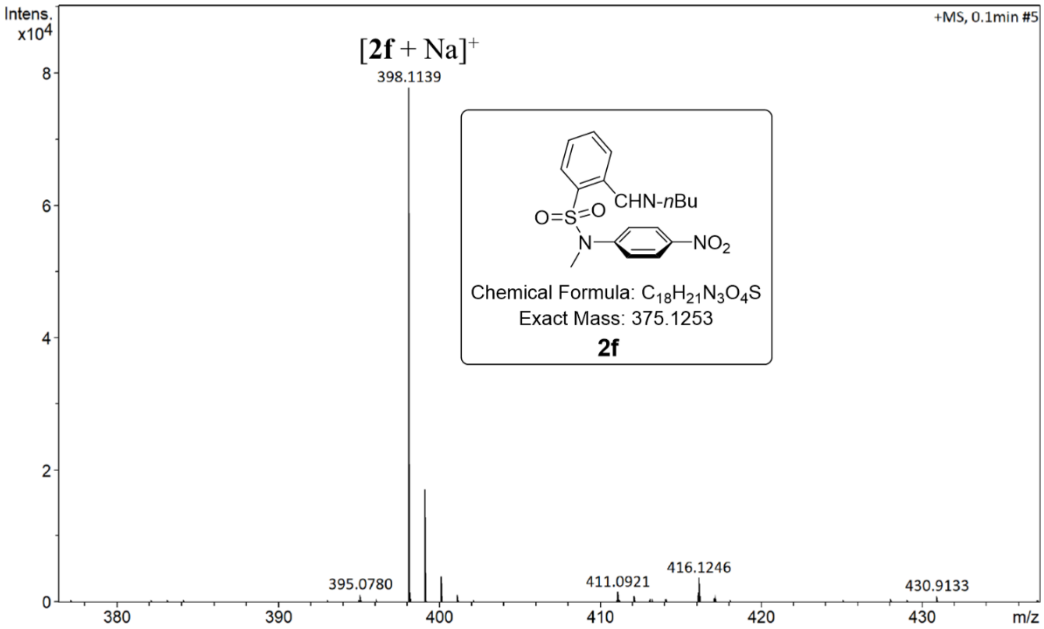

Figure S51. ESI-MS spectrum of the reaction of **1f** and 1-butylamine in CD3CN.

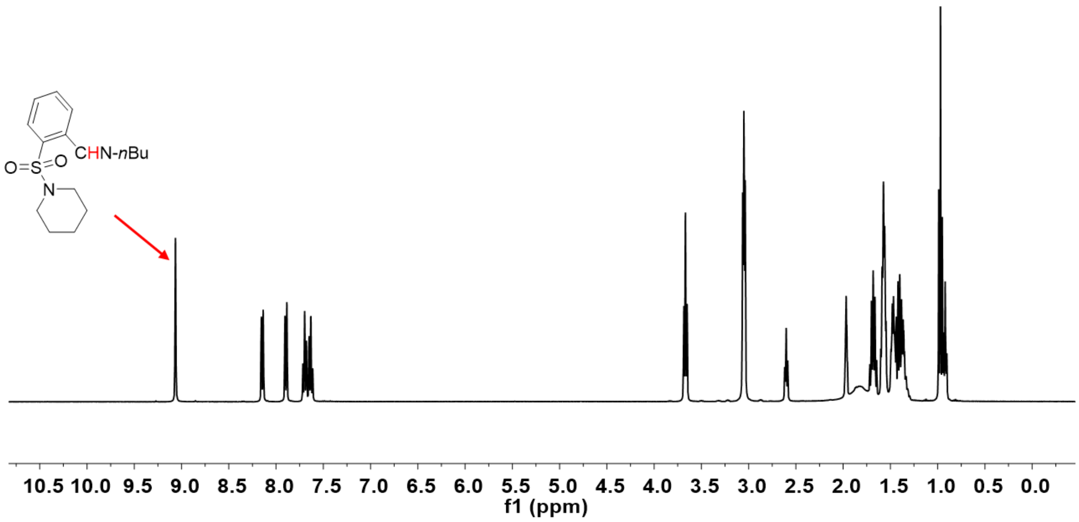

Figure S52. ^{1}H NMR spectrum of the reaction of **1g** and 1-butylamine in CD3CN.

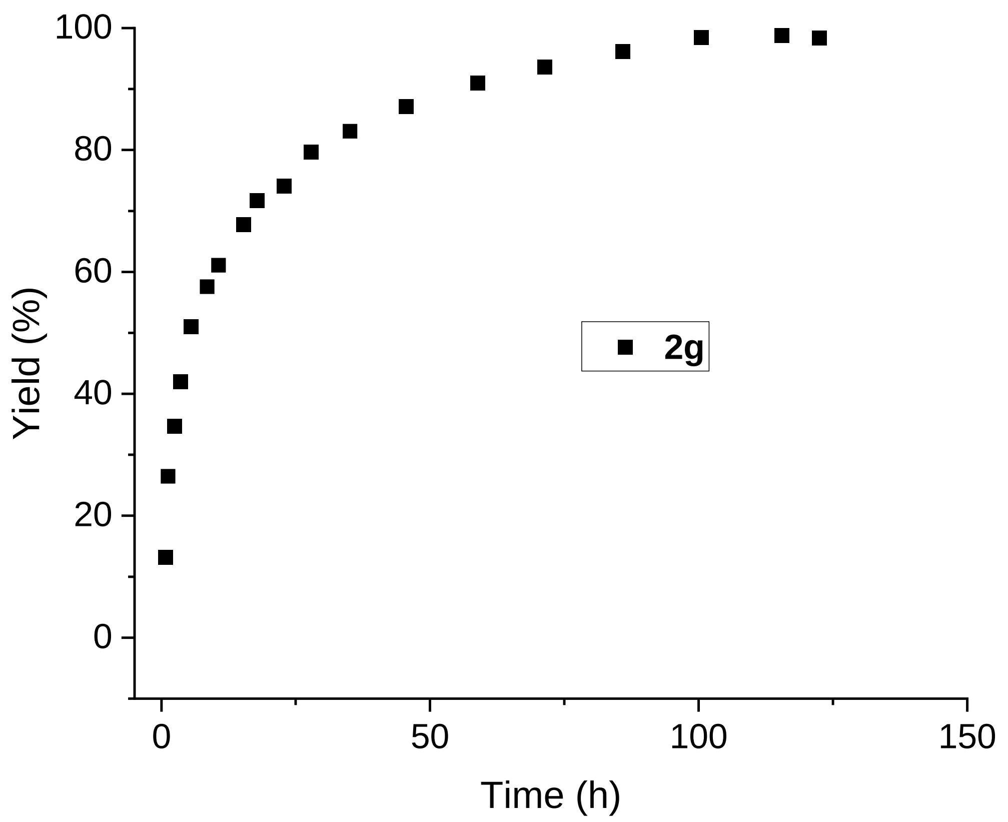

Figure S53. Kinetic profile of the reaction of **1g** and 1-butylamine in CD_3CN.

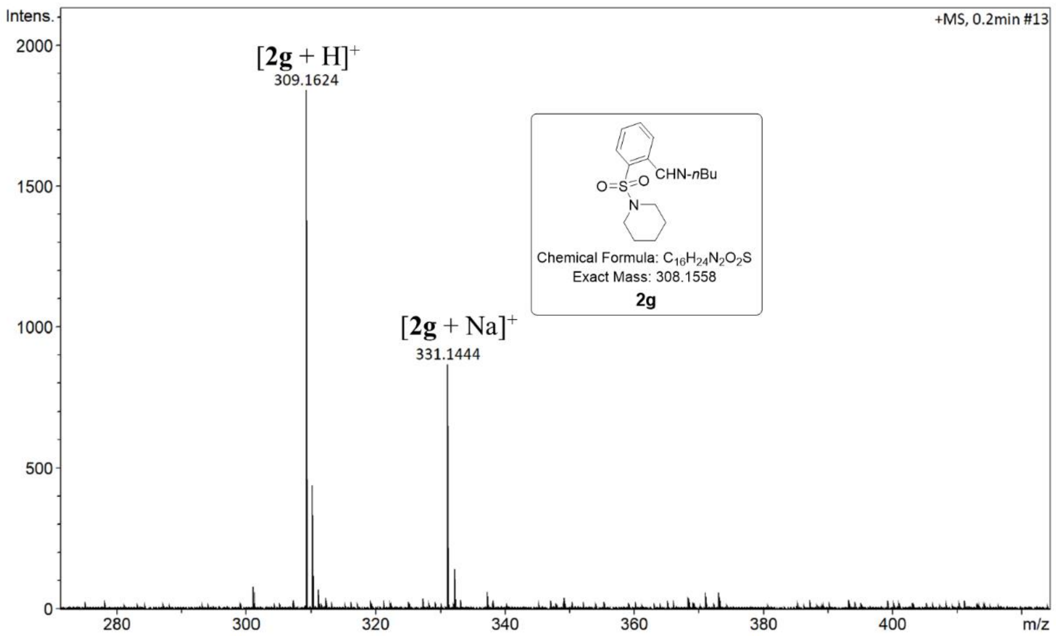

Figure S54. ESI-MS spectrum of the reaction of **1g** and 1-butylamine in CD_3CN.

(2) Imine exchange

Table S10. The equilibrium constant and equilibrating time of the different imine exchange reactions in CD_3CN.

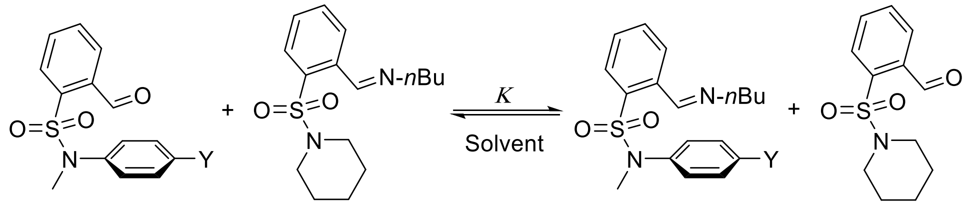

1a - 1f + 2g ⇌ 2a - 2f + 1g

entry	Y	Sequence of adding reagents	Solvent	*K*	Equilibrating time
a	H		CD_3CN	0.598	66 days
b	OMe		CD_3CN	0.634	73 days
c	NMe_2	**1a - 1f** and **1g** simultaneously reacted with 1-butylamine	CD_3CN	0.796	70 days
d	Cl		CD_3CN	1.47	37 days
e	CF_3		CD_3CN	1.66	68 days
f	NO_2		CD_3CN	1.21	49 days

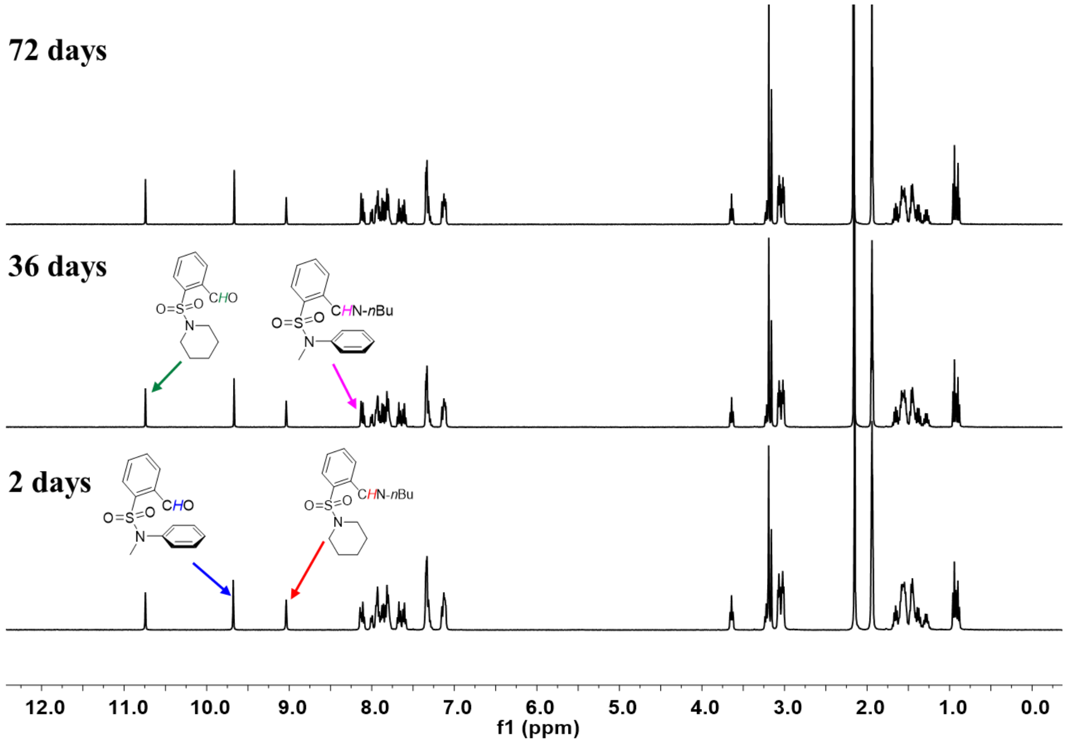

Figure S55. ^{1}H NMR spectra of the competition between **1a** and **1g** for the reaction with 1-butylamine in CD_3CN at varied time (the corresponding spectra of entry a in Table S9).

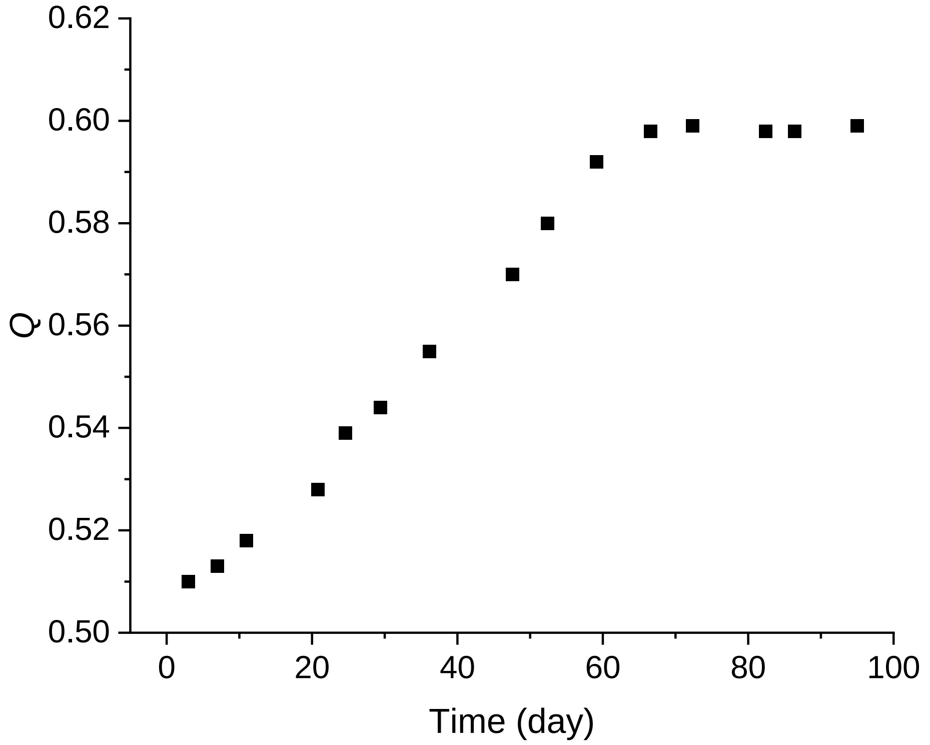

Figure S56. Kinetic profile of the competition between **1a** and **1g** for the reaction with 1-butylamine in CD_3CN.

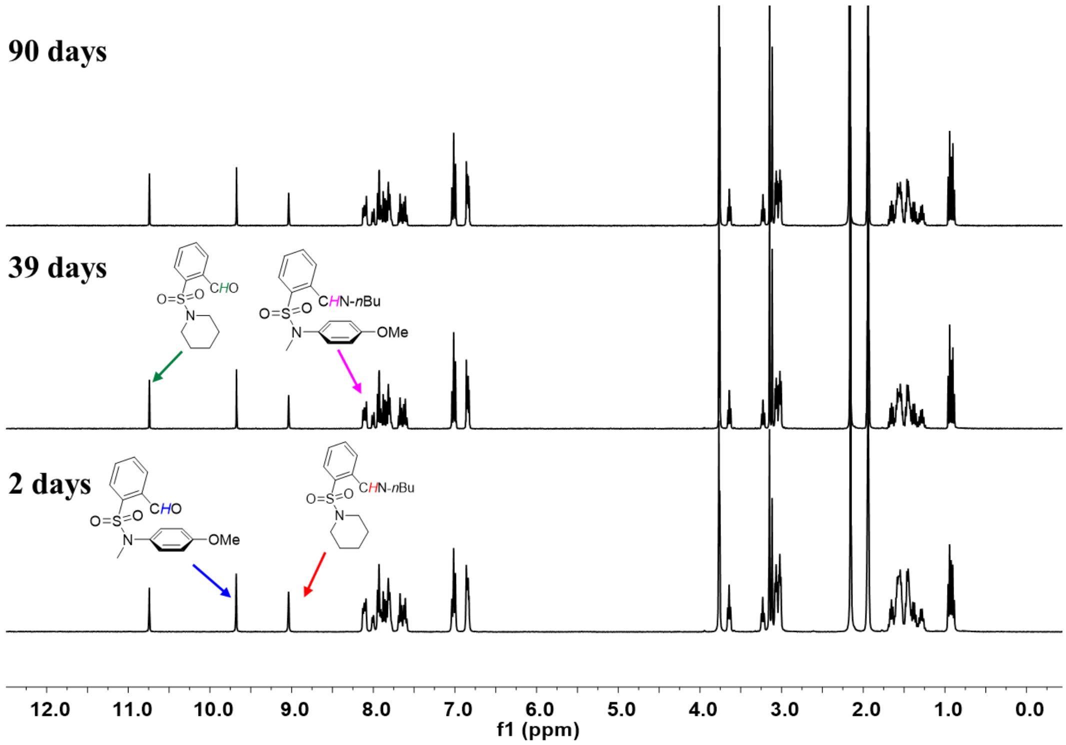

Figure S57. ^{1}H NMR spectra of the competition between **1b** and **1g** for the reaction with 1-butylamine in CD_3CN at varied time (the corresponding spectra of entry b in Table S9).

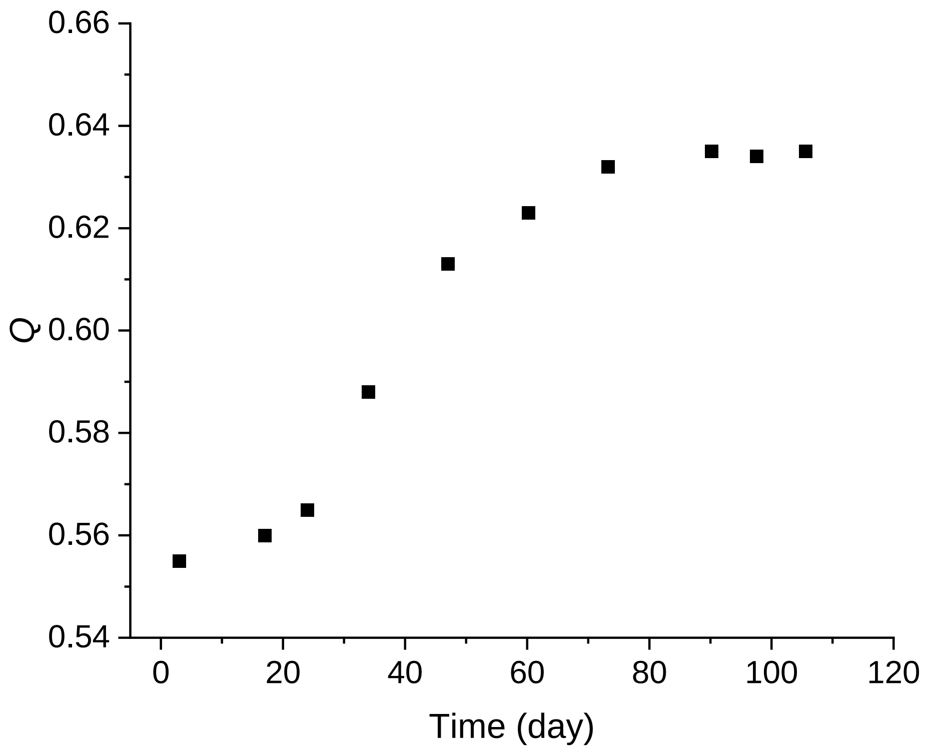

Figure S58. Kinetic profile of the competition between **1b** and **1g** for the reaction with 1-butylamine in CD_3CN.

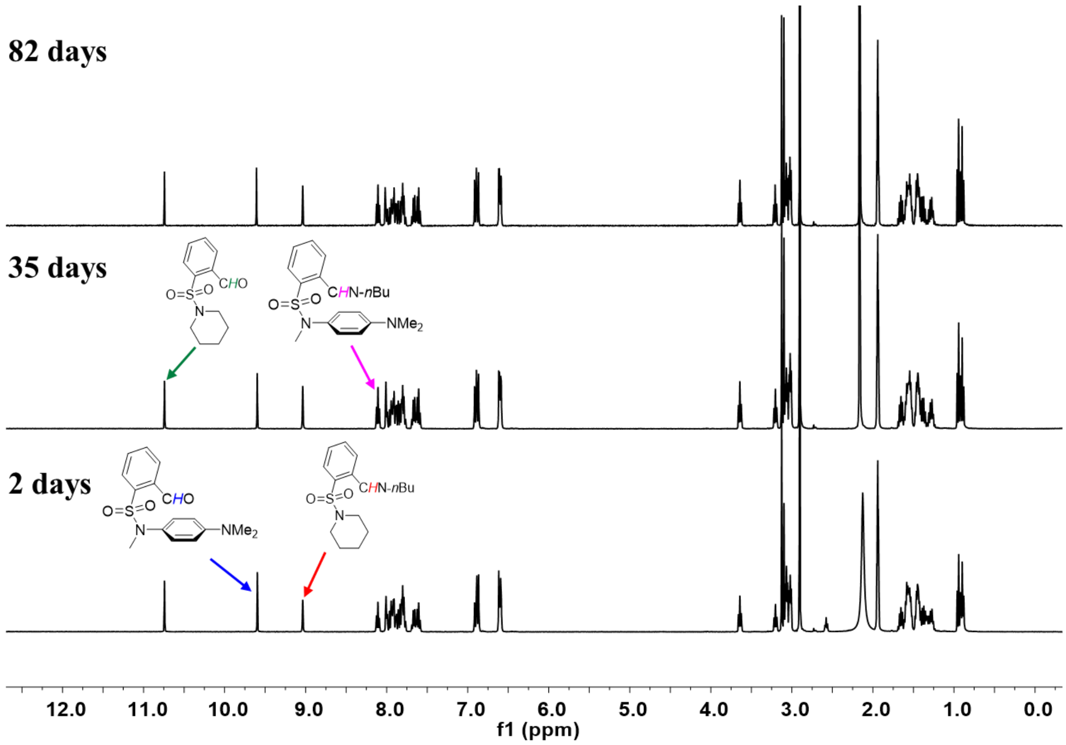

Figure S59. ^{1}H NMR spectra of the competition between **1c** and **1g** for the reaction with 1-butylamine in CD_3CN at varied time (the corresponding spectra of entry c in Table S9).

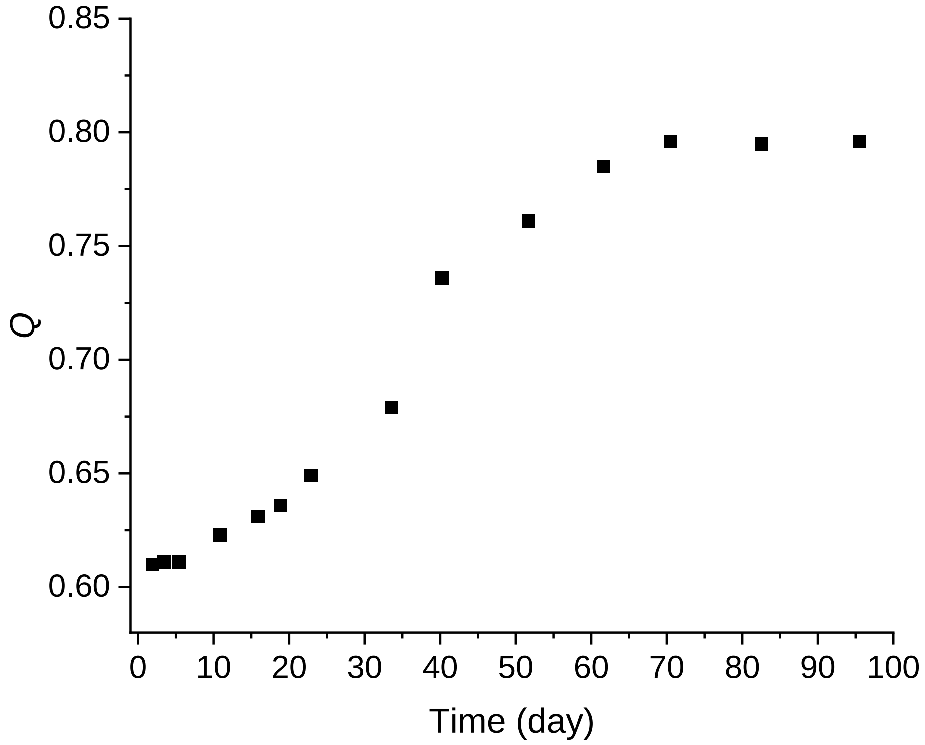

Figure S60. Kinetic profile of the competition between **1c** and **1g** for the reaction with 1-butylamine in CD_3CN.

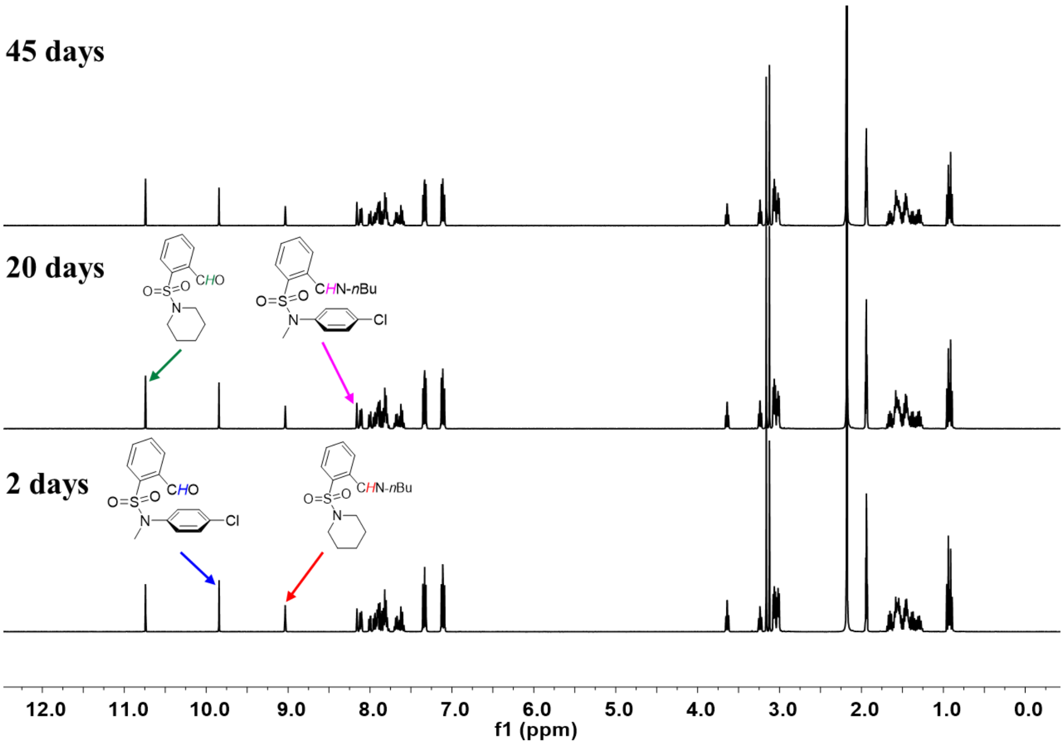

Figure S61. ^{1}H NMR spectra of the competition between **1d** and **1g** for the reaction with 1-butylamine in CD_3CN at varied time (the corresponding spectra of entry d in Table S9).

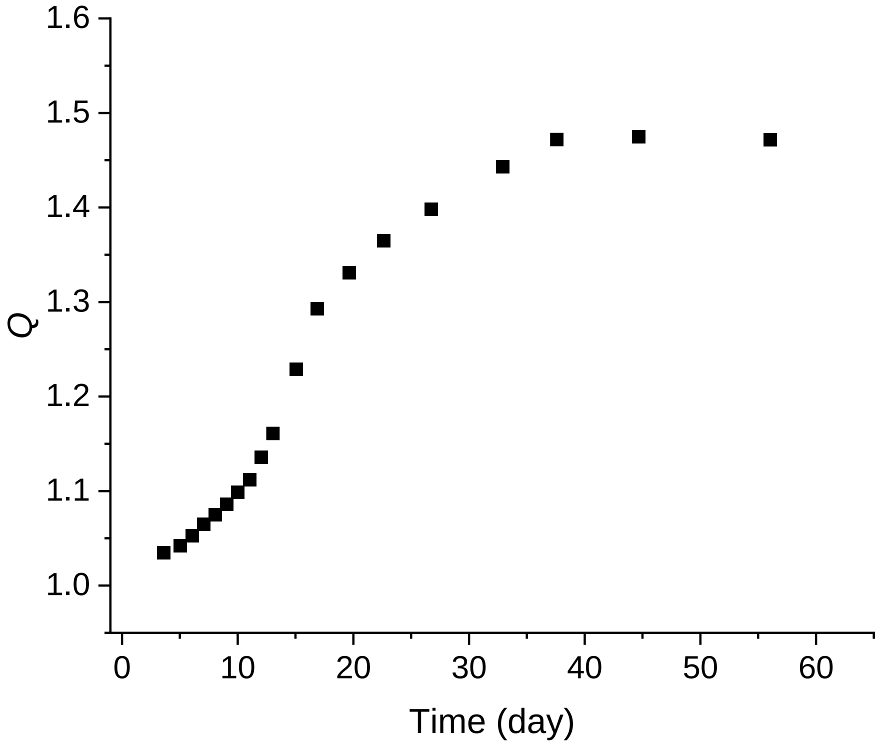

Figure S62. Kinetic profile of the competition between **1d** and **1g** for the reaction with 1-butylamine in CD_3CN.

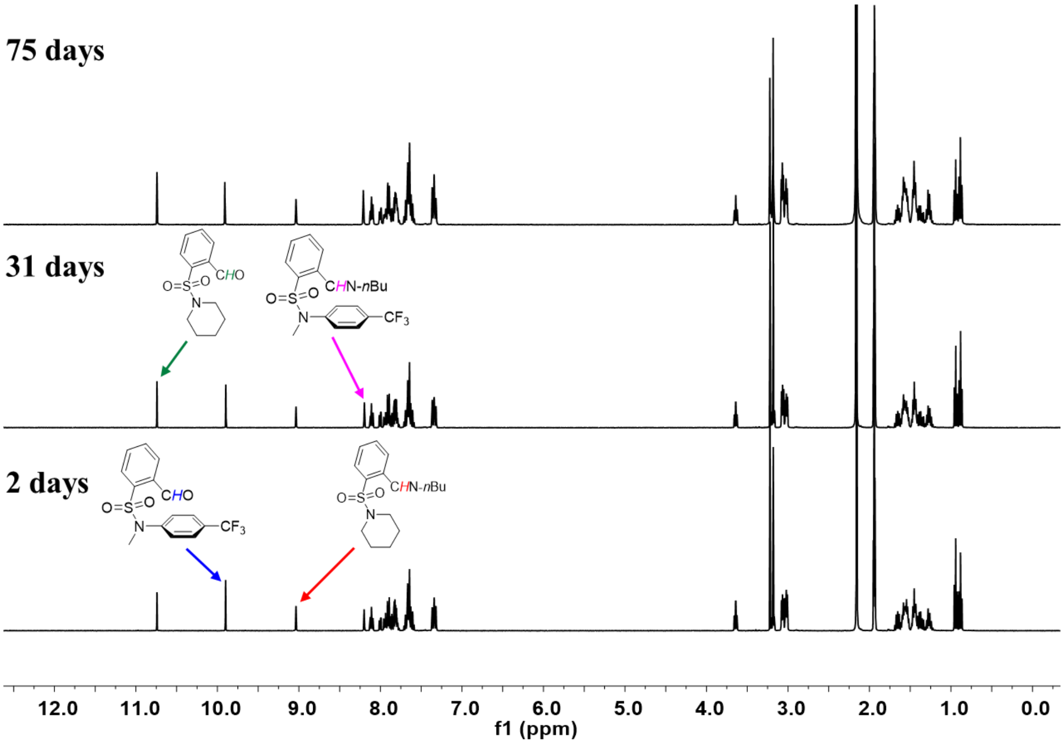

Figure S63. ^{1}H NMR spectra of the competition between **1e** and **1g** for the reaction with 1-butylamine in CD_3CN at varied time (the corresponding spectra of entry e in Table S9).

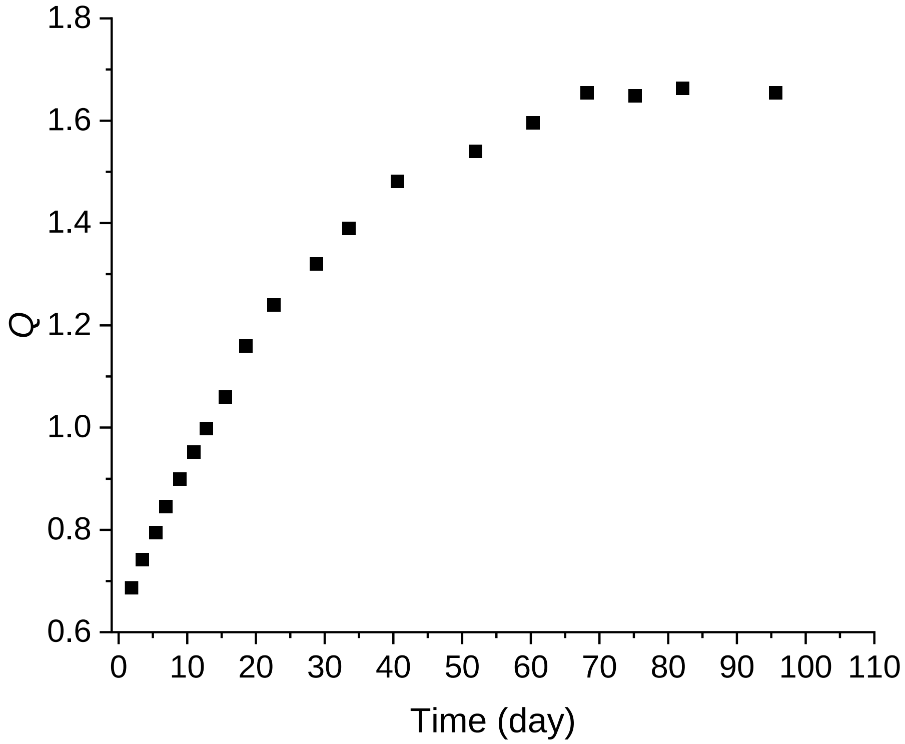

Figure S64. Kinetic profile of the competition between **1e** and **1g** for the reaction with 1-butylamine in CD_3CN.

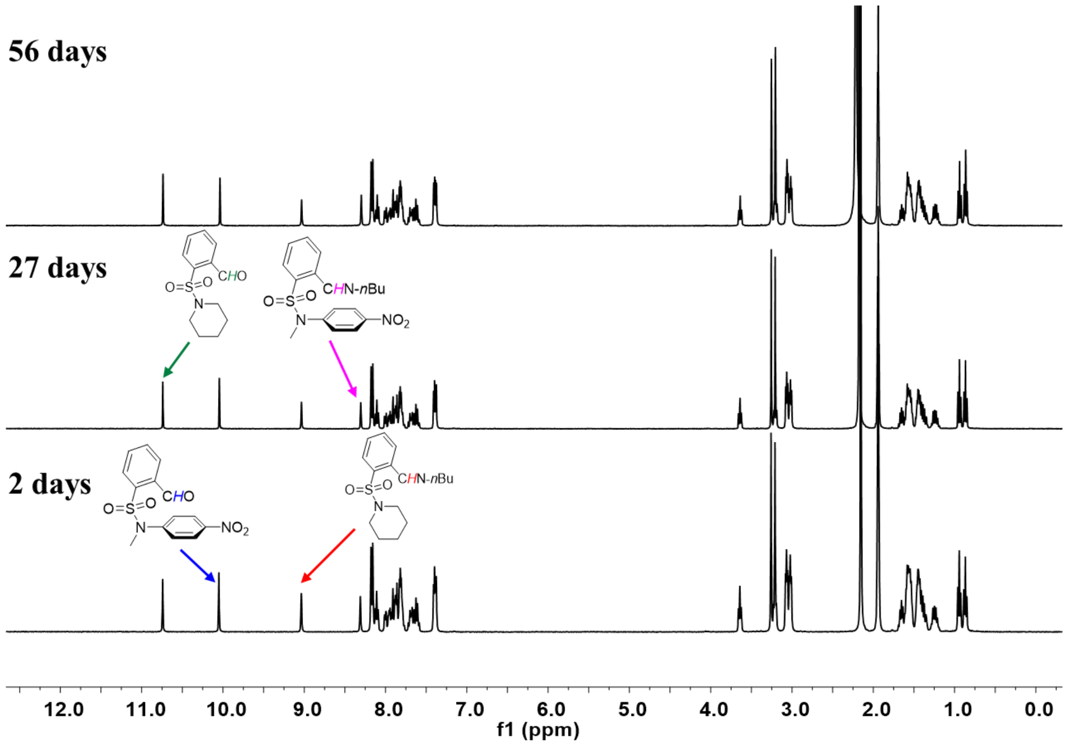

Figure S65. ^{1}H NMR spectra of the competition between **1f** and **1g** for the reaction with 1-butylamine in CD_3CN at varied time (the corresponding spectra of entry f in Table S9).

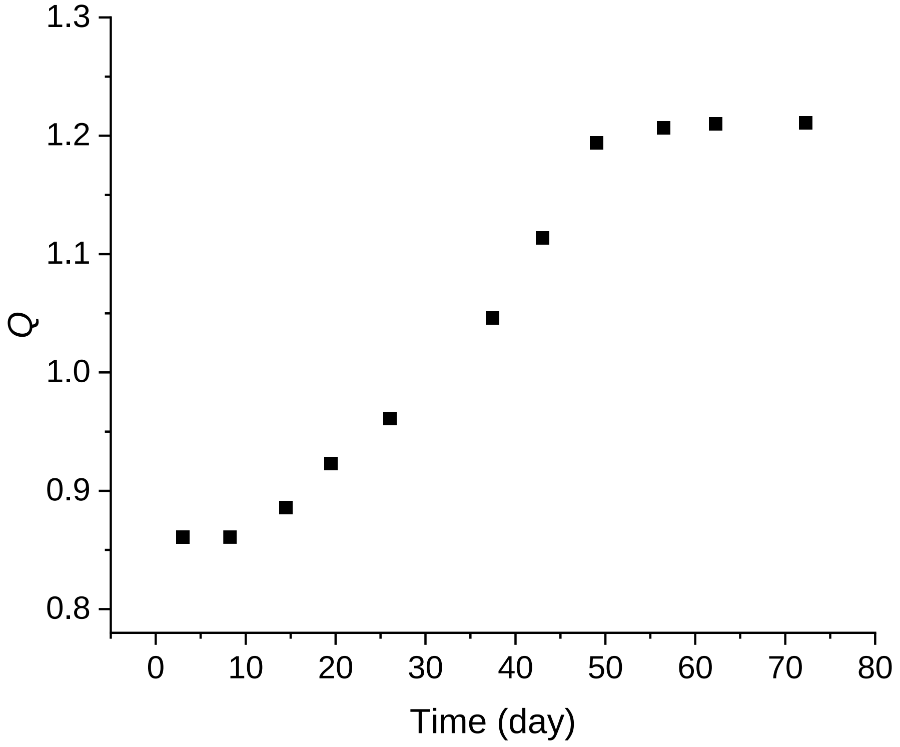

Figure S66. Kinetic profile of the competition between **1f** and **1g** for the reaction with 1-butylamine in CD_3CN.

5. Solvent Effect

Table S11. Cohesive energy density (*ced*),[S11] *K*, equilibrating time, and measured ΔG of exchange reactions.

**Solvent**	***ced*** (cal/cm^3)	*K*	Equilibrating time	-ΔG$_{exp.}$ (kcal/mol)
Toluene	79.4	1.21	9 days	0.109
Chloroform	85.4	0.995	12 days	-0.003
THF	86.9	0.961	8 days	-0.023
Dichloromethane	93.7	0.973	10 days	-0.016
Pyridine	112.4	1.13	7 days	0.071
Acetonitrile	138.9	0.598	66 days	-0.299
DMF	139.2	0.385	2 days	-0.556
DMSO	168.6	0.397	6 days	-0.538

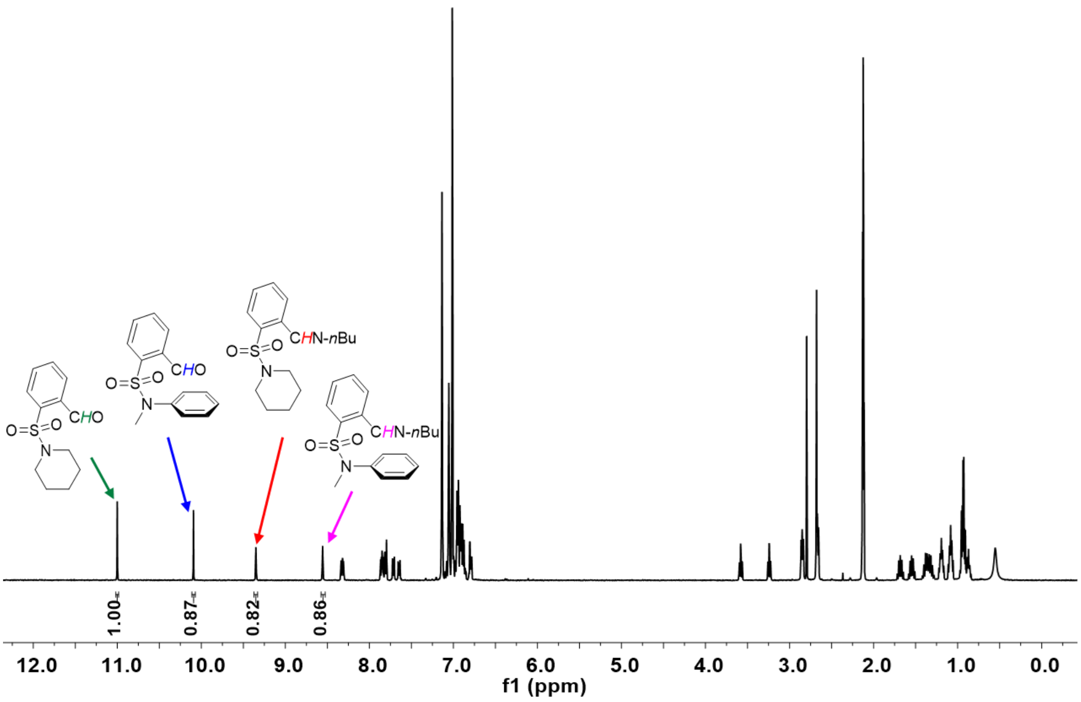

Figure S67. ^{1}H NMR spectrum of the competition between **1a** and **1g** for the reaction with 1-butylamine in toluene-d_8.

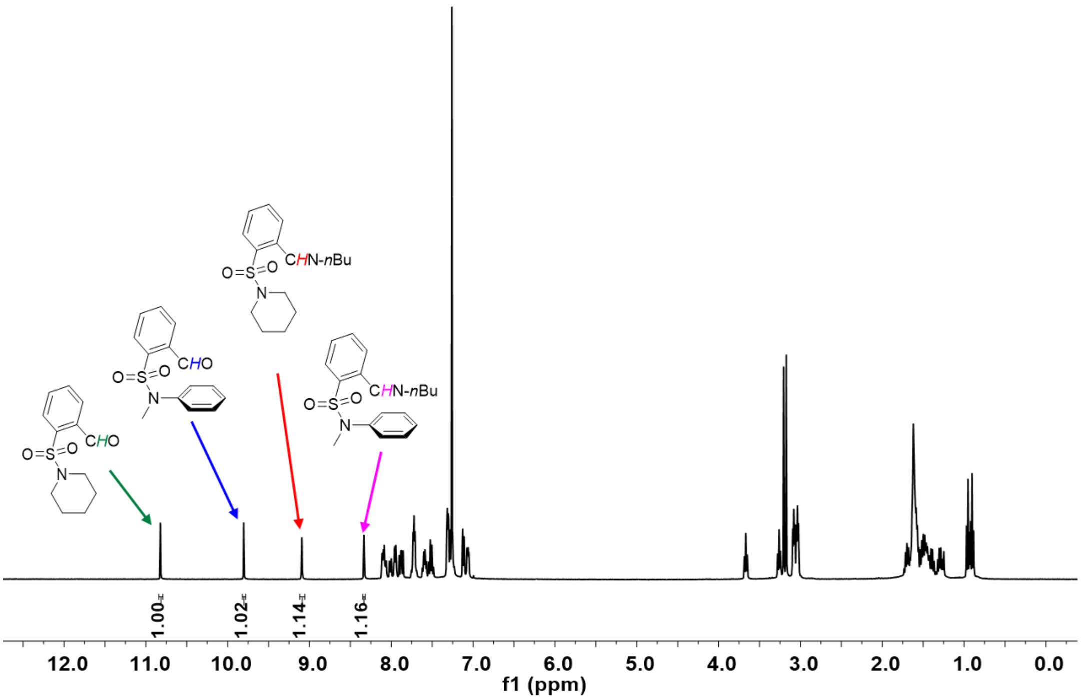

Figure S68. ^{1}H NMR spectrum of the competition between **1a** and **1g** for the reaction with 1-butylamine in $CDCl_3$.

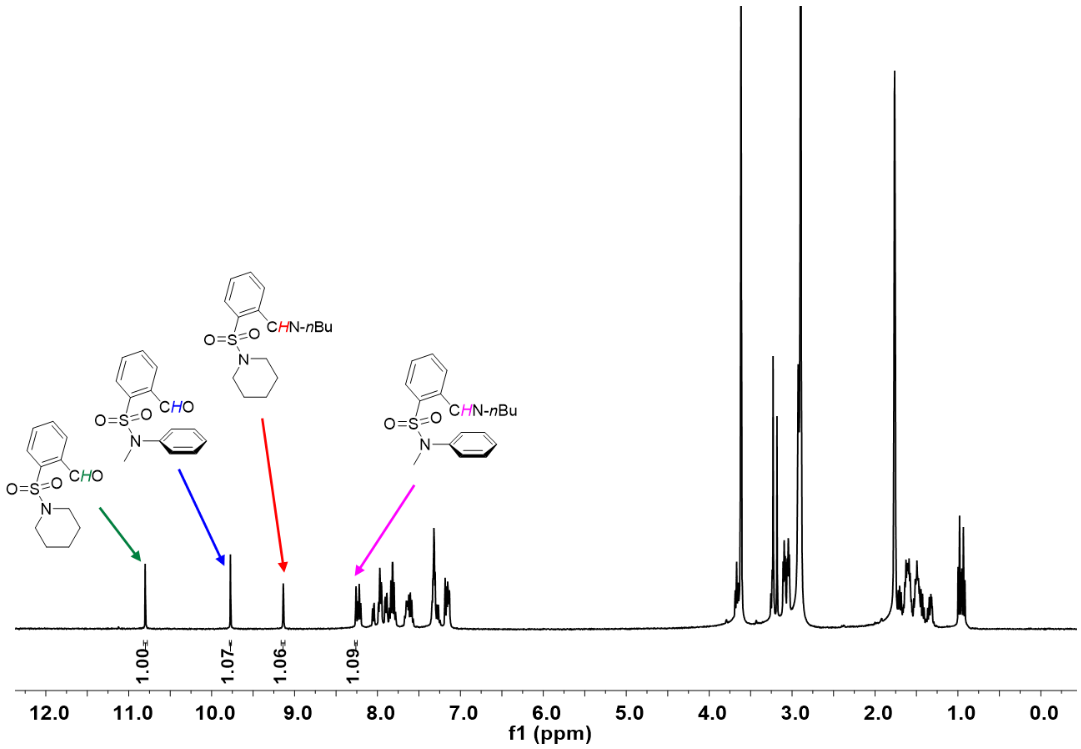

Figure S69. ^{1}H NMR spectrum of the competition between **1a** and **1g** for the reaction with 1-butylamine in THF-d_8.

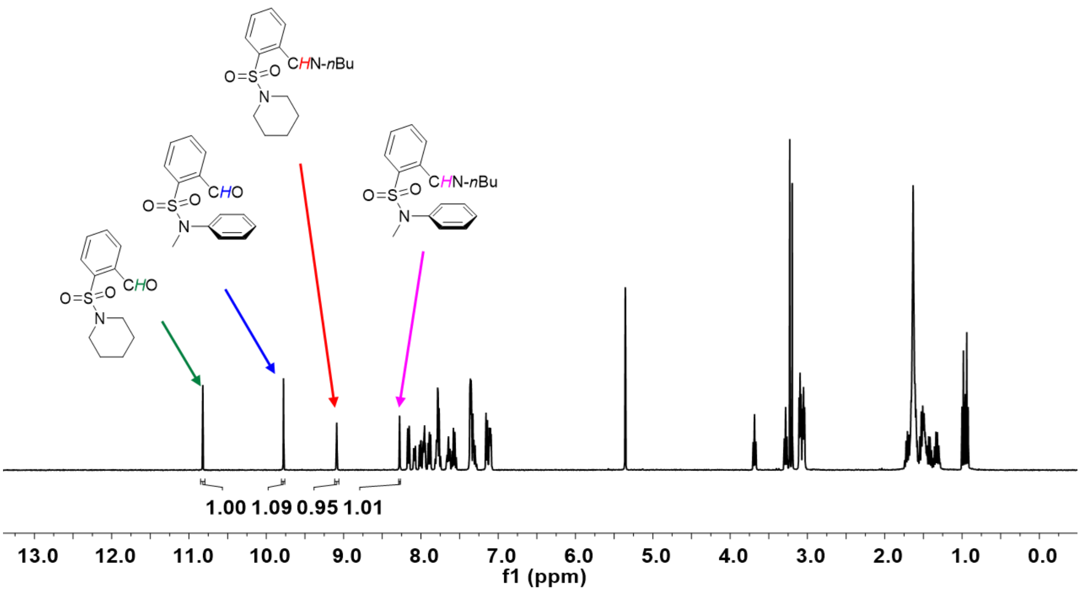

Figure S70. ^{1}H NMR spectrum of the competition between **1a** and **1g** for the reaction with 1-butylamine in CD_2Cl_2.

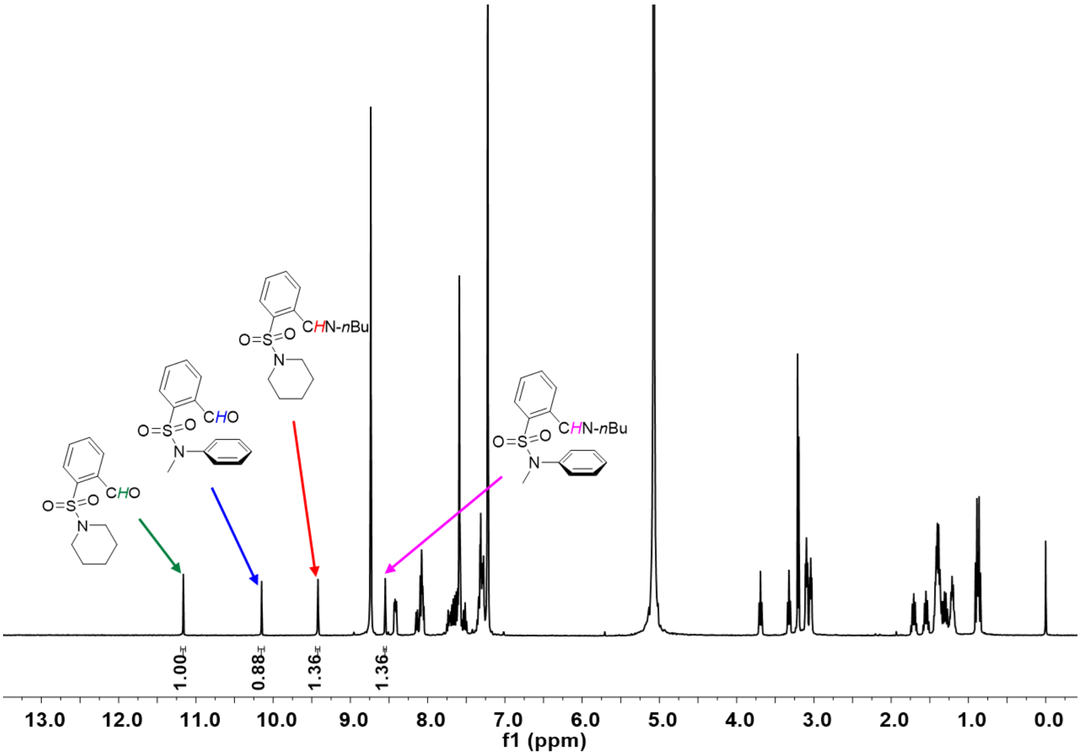

Figure S71. ^{1}H NMR spectrum of the competition between **1a** and **1g** for the reaction with 1-butylamine in pyridine-d_5.

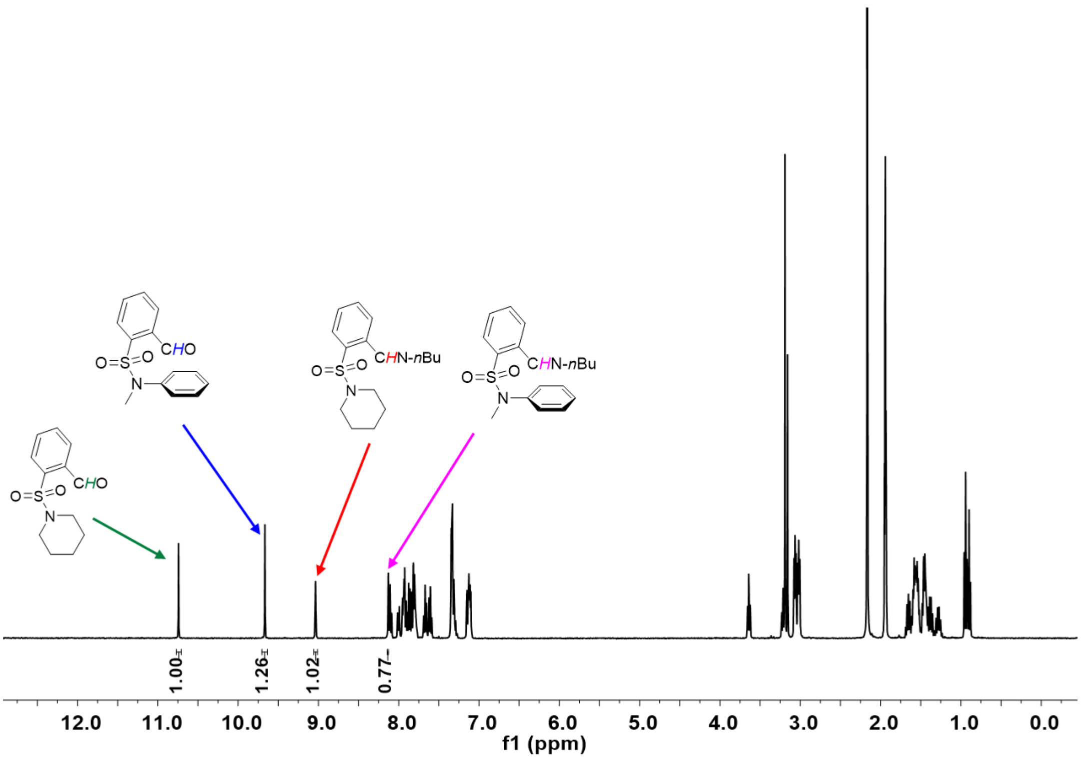

Figure S72. ^{1}H NMR spectrum of the competition between **1a** and **1g** for the reaction with 1-butylamine in CD_3CN.

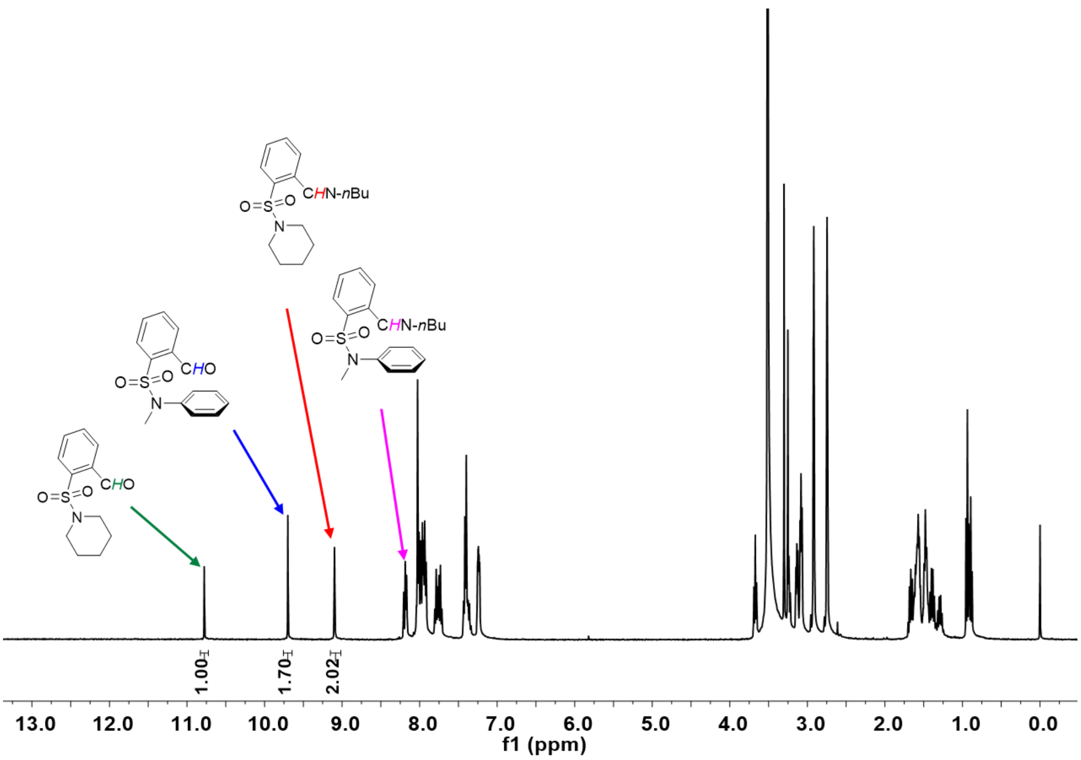

Figure S73. ^{1}H NMR spectrum of the competition between **1a** and **1g** for the reaction with 1-butylamine in DMF-d_7.

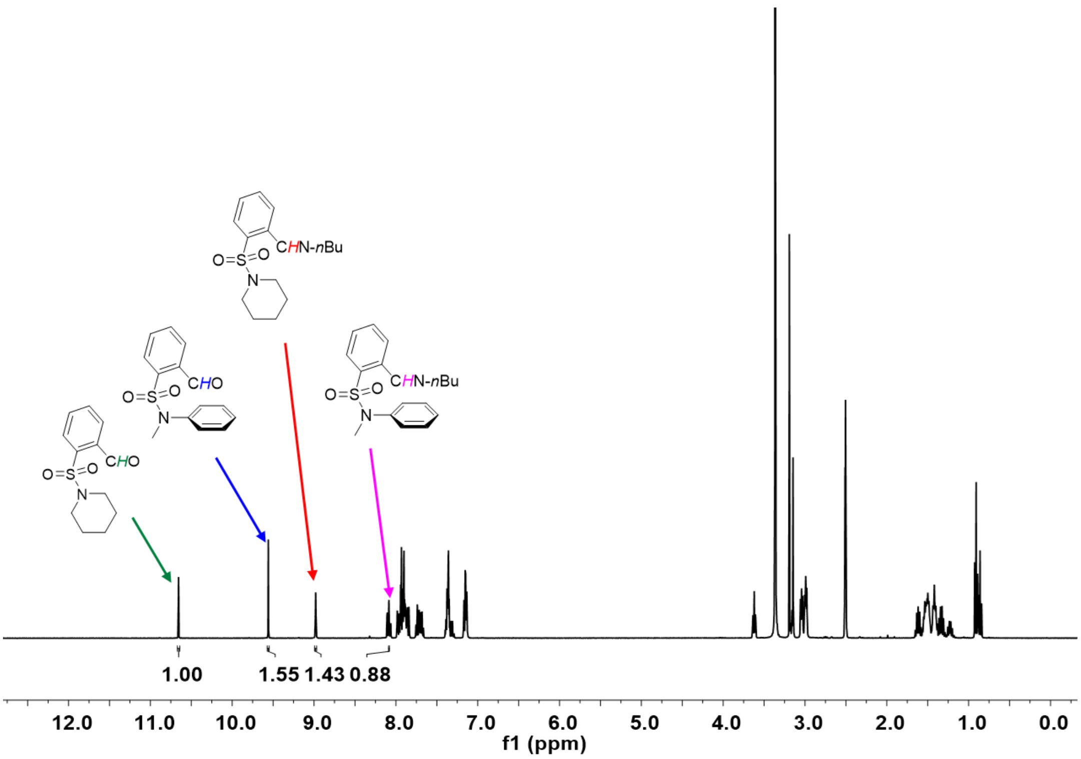

Figure S74. ^{1}H NMR spectrum of the competition between **1a** and **1g** for the reaction with 1-butylamine in DMSO-d_6.

Table S12. Calculated NPA charge (e) of the atoms located in the C=X bond (X = O/NMe) for **1** and **2**.

**1a**	C	O	**2a**	C	N
gas	3.921E-01	-5.149E-01	gas	7.680E-02	-3.842E-01
Toluene	3.992E-01	-5.326E-01	Toluene	7.896E-02	-3.915E-01
Chloroform	4.029E-01	-5.425E-01	Chloroform	8.013E-02	-3.958E-01
THF	4.045E-01	-5.469E-01	THF	8.064E-02	-3.979E-01
ACN	4.058E-01	-5.505E-01	ACN	8.107E-02	-3.997E-01
DMF	4.069E-01	-5.539E-01	DMF	8.146E-02	-4.014E-01

6. Control over Thermodynamic and Kinetic Selectivity

Table S13. The equilibrium constant and equilibrating time of the different imine exchange reactions in CD_3CN.

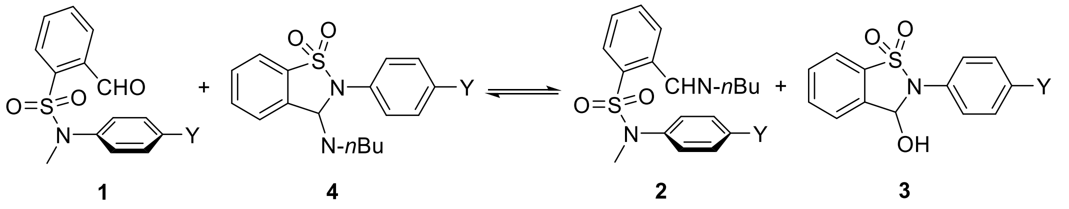

entry	Y	Sequence of adding reagents	Solvent	*K*	Equilibrating time
a	H	**1** and **3** simultaneously reacted with 1-butylamine	CD_3CN	1.73	50 days
b	OMe		CD_3CN	1.94	119 days
c	NO_2		CD_3CN	2.74	107 days

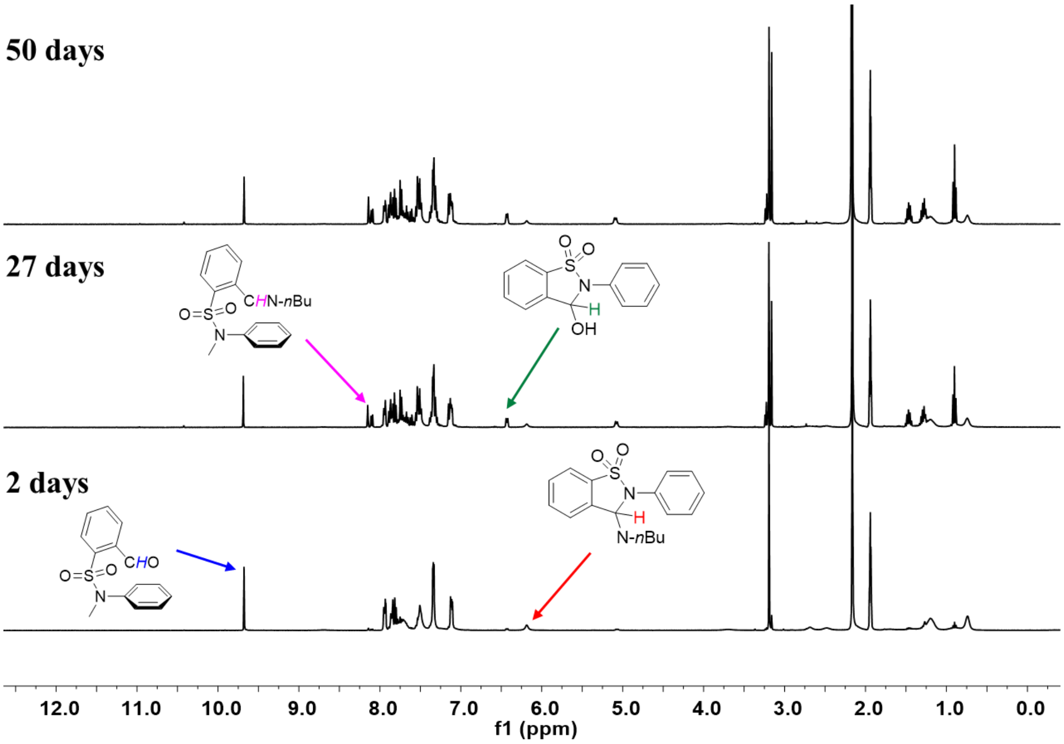

Figure S75. ^1H NMR spectra of the competition between **1a** and **3a** for the reaction with 1-butylamine in CD_3CN at varied time (the corresponding spectra of entry a in Table S12).

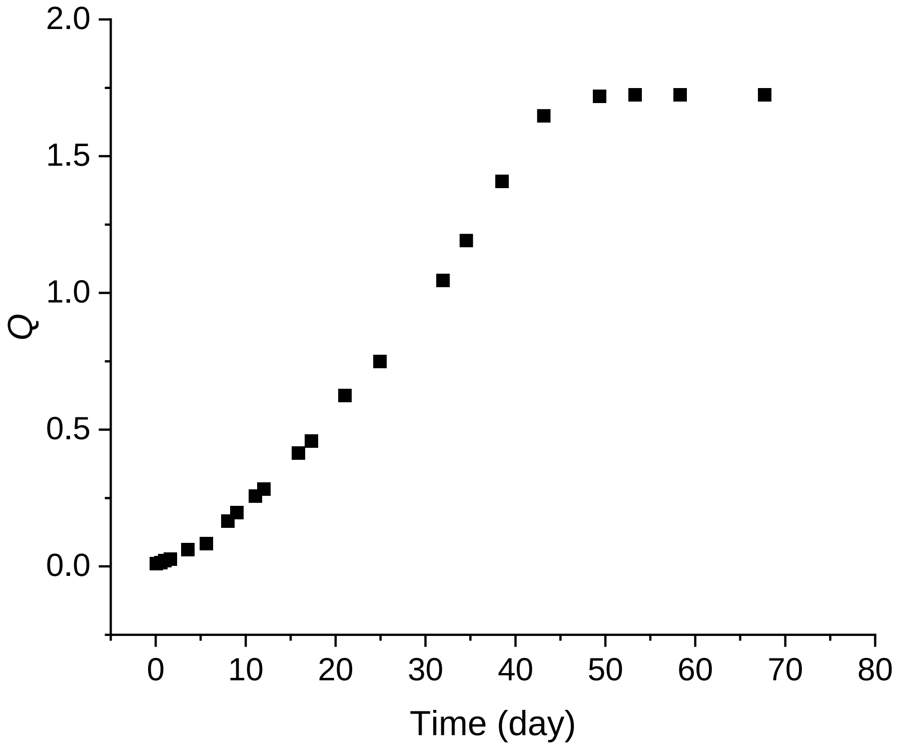

Figure S76. Kinetic profile of the competition between **1a** and **3a** for the reaction with 1-butylamine in CD_3CN.

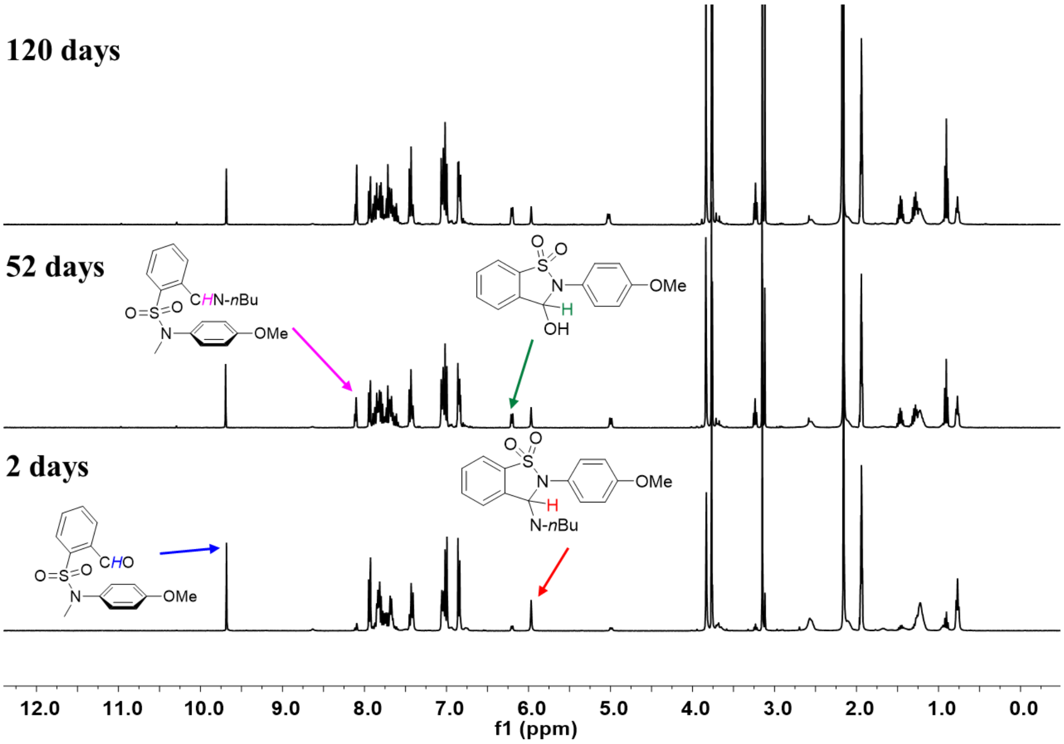

Figure S77. ^{1}H NMR spectra of the competition between **1b** and **3b** for the reaction with 1-butylamine in CD_3CN at varied time (the corresponding spectra of entry b in Table S12).

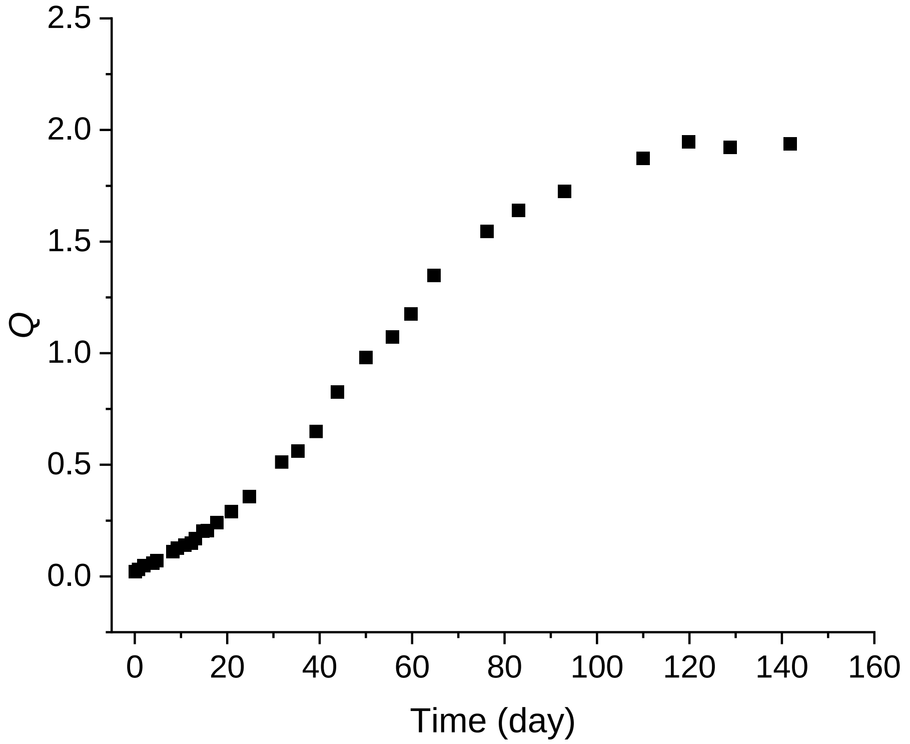

Figure S78. Kinetic profile of the competition between **1b** and **3b** for the reaction with 1-butylamine in CD_3CN.

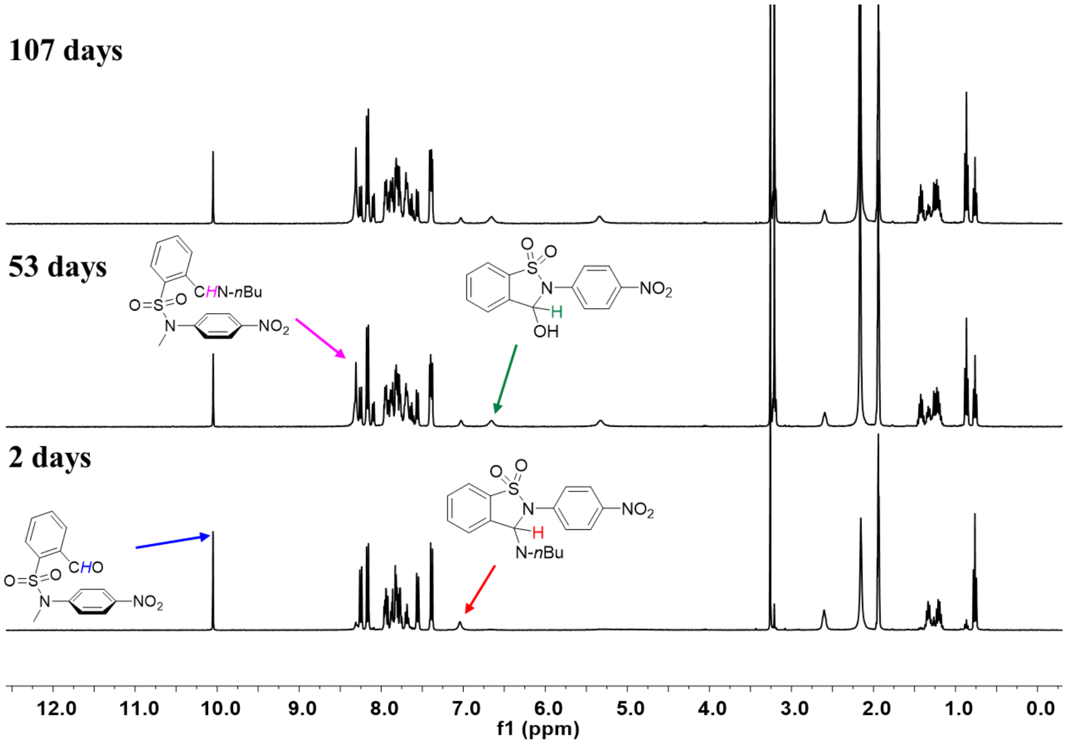

Figure S79. ^{1}H NMR spectra of the competition between **1f** and **3f** for the reaction with 1-butylamine in CD_3CN at varied time (the corresponding spectra of entry c in Table S12).

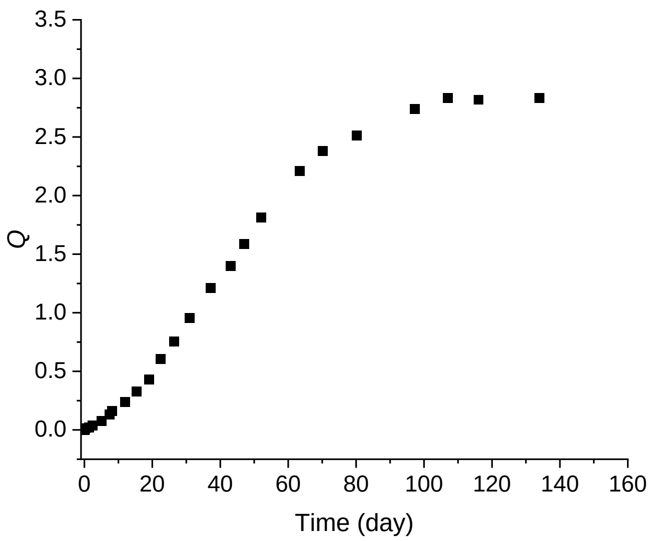

Figure S80. Kinetic profile of the competition between **1f** and **3f** for the reaction with 1-butylamine in CD_3CN.

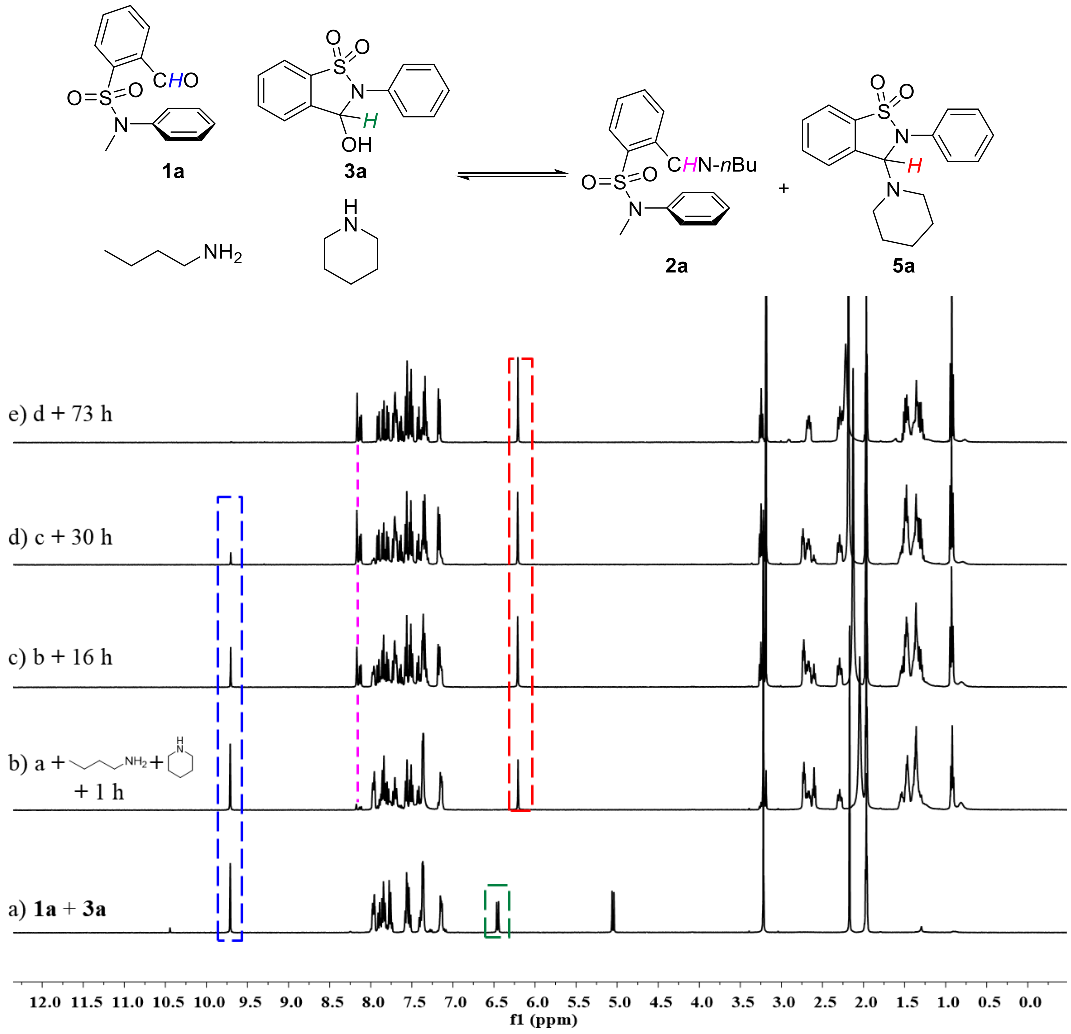

Figure S81. ^{1}H NMR spectra of the mixture generated from equal amounts of **1a** + **3a** + 1-BuNH2 + piperidine (12 mM each, CD_3CN) at varied time.

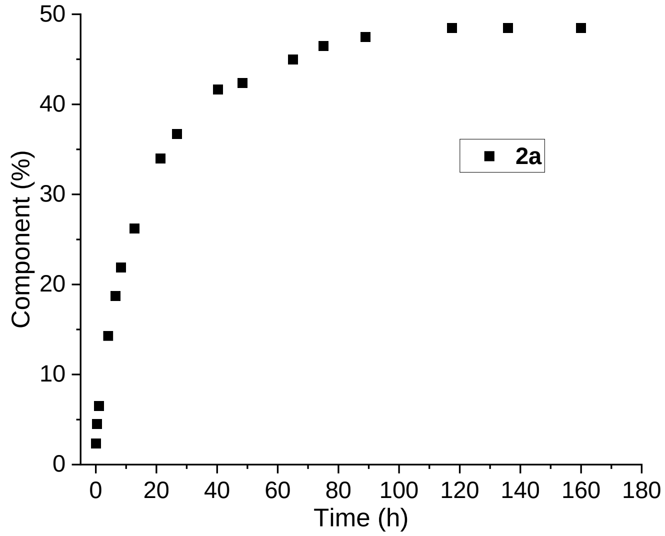

Figure S82. Kinetic profile of the mixture from equal amounts of **1a** + **3a** + 1-BuNH$_2$ + piperidine in CD_3CN.

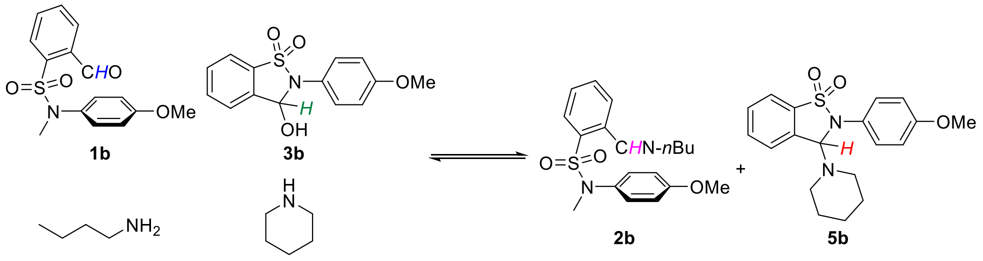

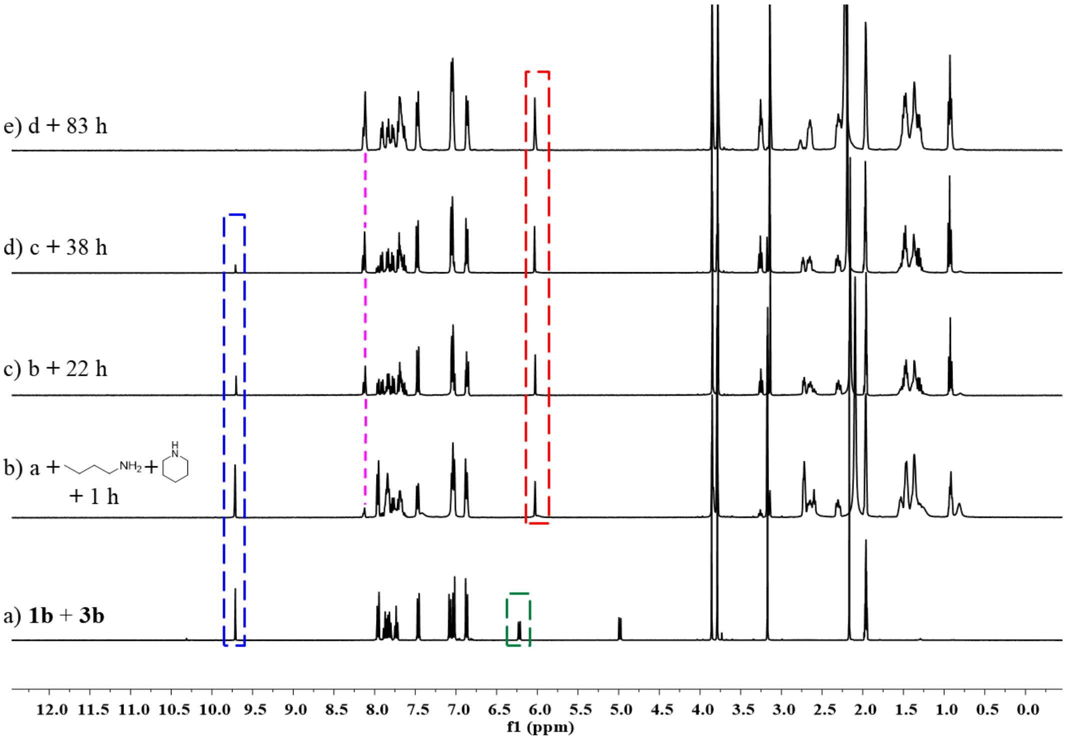

Figure S83. ^{1}H NMR spectra of the mixture generated from equal amounts of **1b** + **3b** + 1-BuNH$_2$ + piperidine (12 mM each, CD_3CN) at varied time.

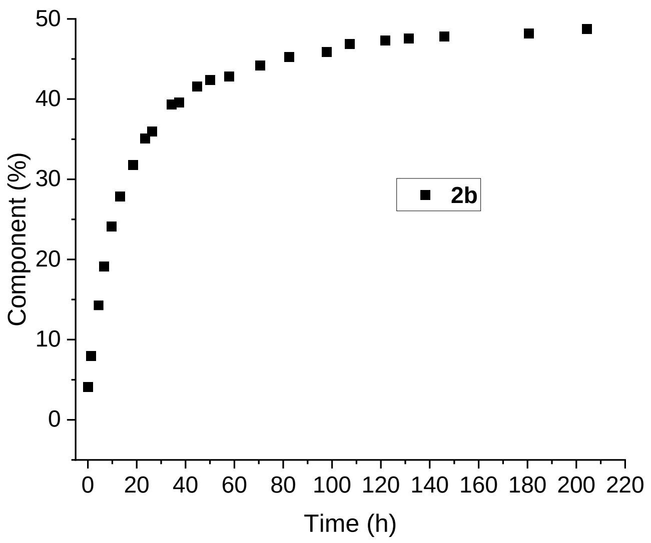

Figure S84. Kinetic profile of the mixture from equal amounts of **1b** + **3b** + 1-BuNH$_2$ + piperidine in CD$_3$CN.

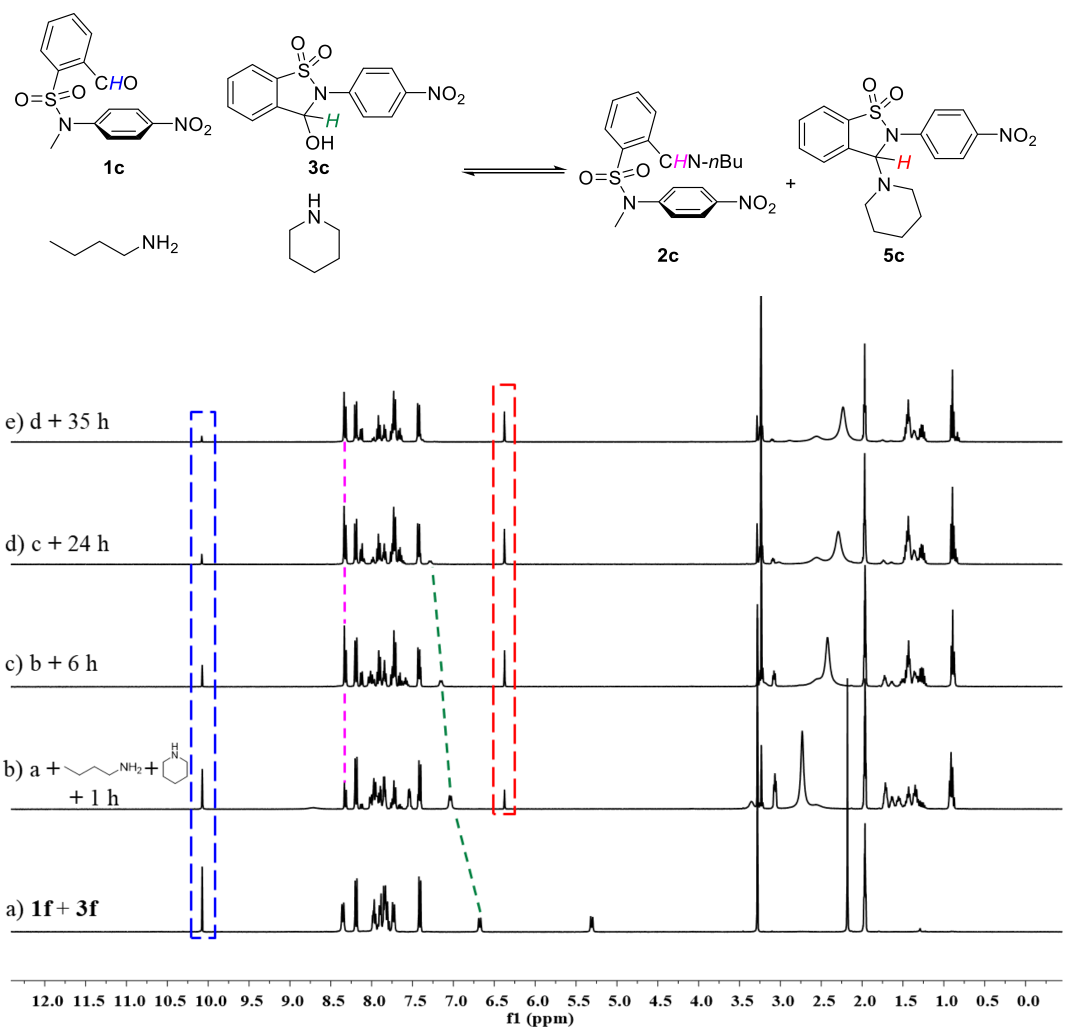

Figure S85. ¹H NMR spectra of the mixture generated from equal amounts of **1c** + **3c** + 1-BuNH$_2$ + piperidine (12 mM each, CD$_3$CN) at varied time.

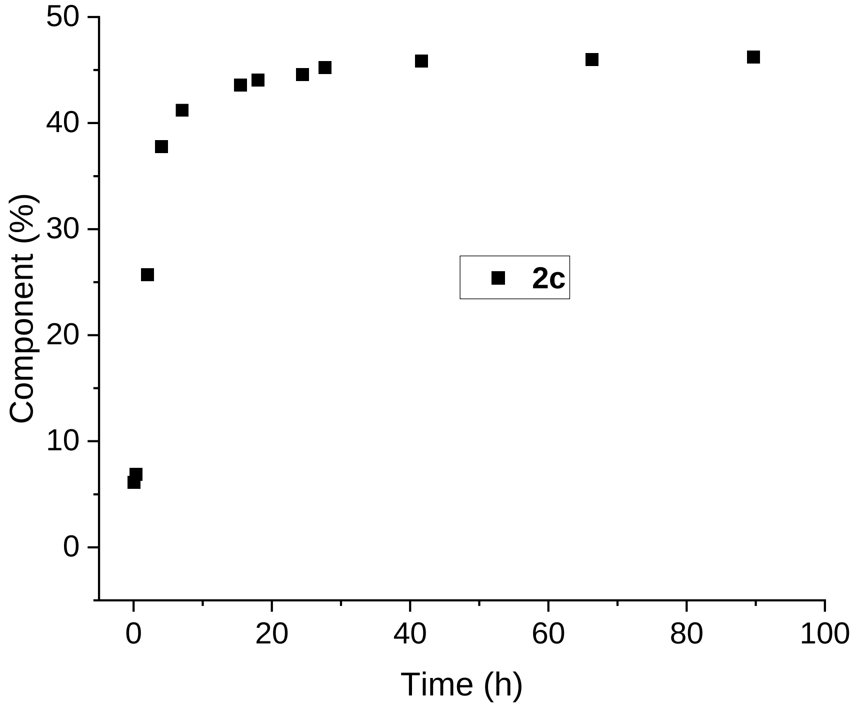

Figure S86. Kinetic profile of the mixture from equal amounts of **1c** + **3c** + 1-BuNH$_2$ + piperidine in CD_3CN.

7. Regulation of Fluorescence

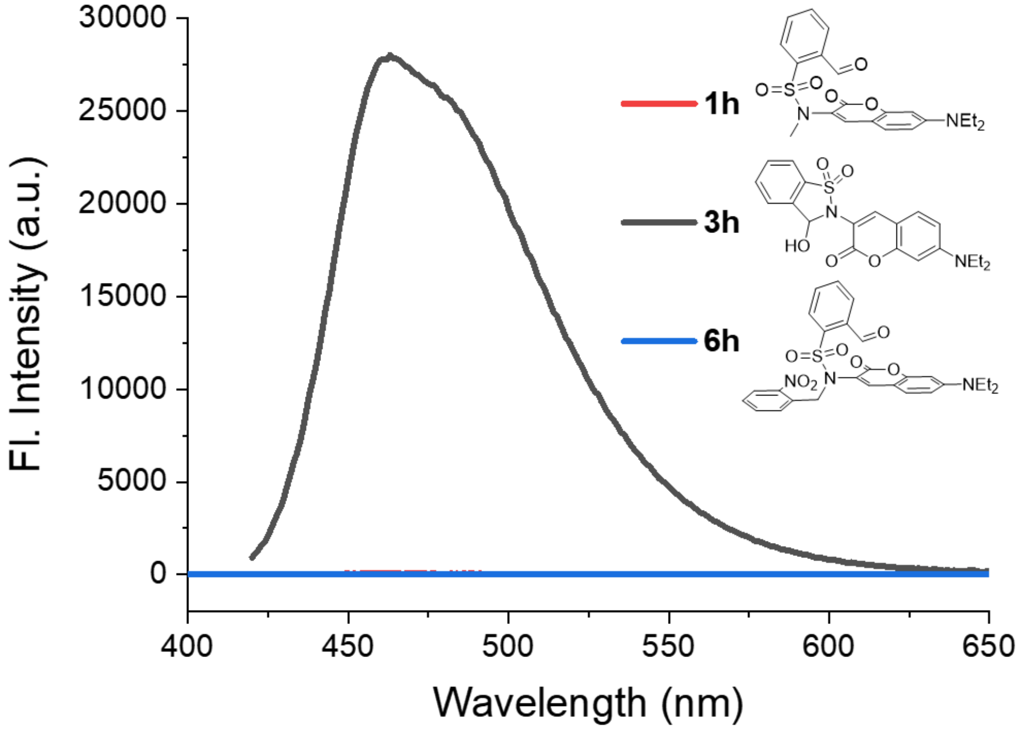

Figure S87. Fluorescence spectra of **1h** (λ_{ex} = 368 nm), **3h** (λ_{ex} = 380 nm), and **6h** (λ_{ex} = 372 nm) in CH_3CN.

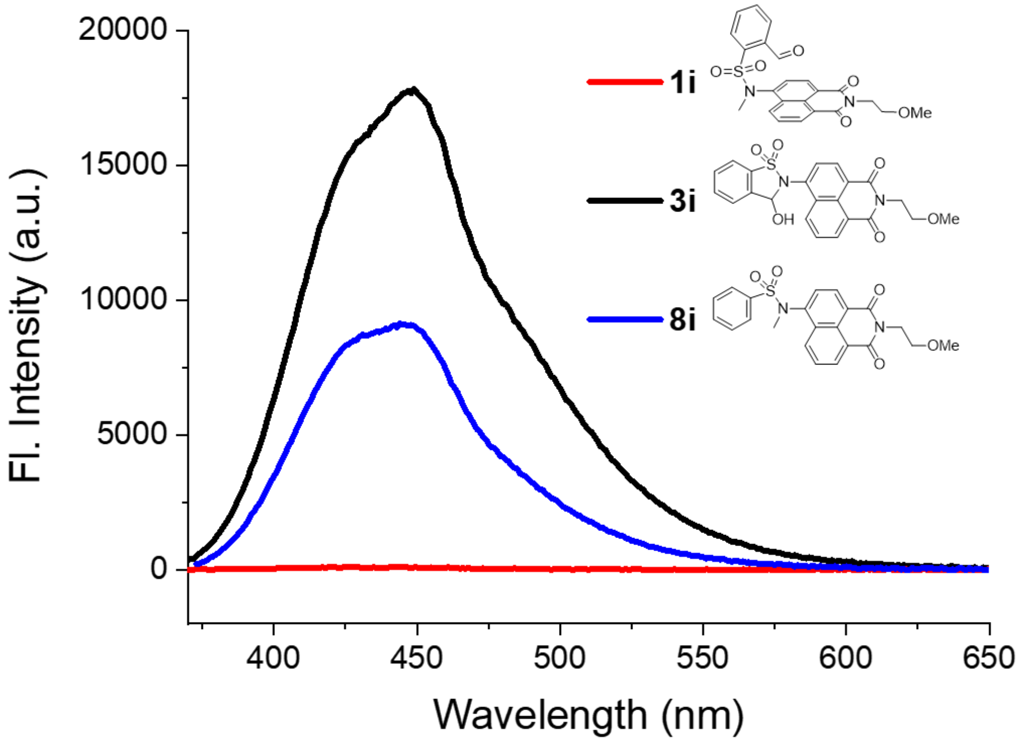

Figure S88. Fluorescence spectra of **1i** (λ_{ex} = 348 nm), **3i** (λ_{ex} = 350 nm) and **8i** (λ_{ex} = 353 nm) in CH_3CN.

Table S14. Selected parameters for the vertical excitation (UV-vis absorption) of the compounds. Electronic excitation energies (eV), oscillator strengths (f), and configurations of the low-lying excited states of **1h**, **2h**, and the control of **1h** without formyl group.

Compounds	Electronic transition[a]	Excitation energy[a]	f[b]	Composition[c]	Transition configuration
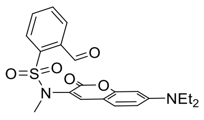<br>**1h**	$S_0 \rightarrow S_1$<br>$S_0 \rightarrow S_2$	2.64 eV<br>(470 nm)<br>3.20 eV<br>(387 nm)	0.2375<br>0.6685	H→L<br>H→L+1	99.3%<br>98.6%
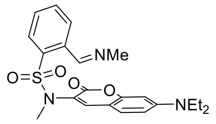<br>**2h**	$S_0 \rightarrow S_1$<br>$S_0 \rightarrow S_2$	3.05 eV<br>(406 nm)<br>3.32 eV<br>(373 nm)	0.6520<br>0.2112	H→L<br>H→L+1	97.1%<br>97.4%
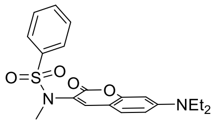<br>**1h** without CHO	$S_0 \rightarrow S_1$	3.14 eV<br>(395 nm)	0.8921	H→L	98.7%

[a] Only selected excited states were considered. The numbers in parentheses are the excitation energy in wavelength. [b] Oscillator strength. [c] H stands for HOMO and L stands for LUMO. Only the main transition configurations (%) are presented.

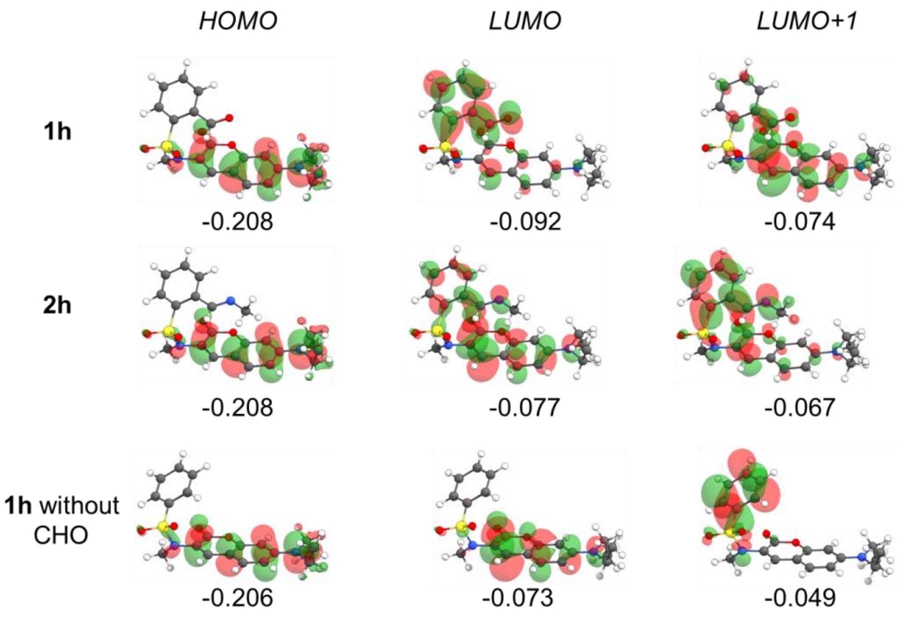

Figure S89. Selected molecular orbitals for the vertical excitation (UV-vis absorption) of **1h**, **2h**, and the control of **1h** without formyl group, with the orbital energy listed.

Table S15. Emission related energy gaps (eV), oscillator strengths (f), and configurations of the low-lying excited states of **1h**, **2h**, and the control of **1h** without formyl group.

Compounds	Electronic transition[a]	Excitation energy[a]	f[b]	Composition[c]	Transition configuration
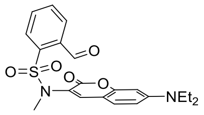 **1h**	$S_0 \rightarrow S_1$	1.82 eV (679 nm)	0.0152	H→L	99.6%
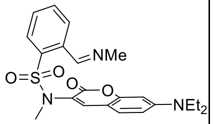 **2h**	$S_0 \rightarrow S_1$	1.99 eV (624 nm)	0.1630	H→L	99.0%
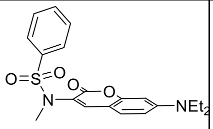 **1h** without CHO	$S_0 \rightarrow S_1$	2.85 eV (434 nm)	0.8671	H→L	98.7%

[a] Only selected excited states were considered. The numbers in parentheses are the excitation energy in wavelength. [b] Oscillator strength. [c] H stands for HOMO and L stands for LUMO. Only the main transition configurations (%) are presented. The oscillator strengths (*f*) of S_1 by TD-DFT calculation increase with the sequence of **1h**, **2h**, and the control of **1h** without formyl group, which validated the experimental data.

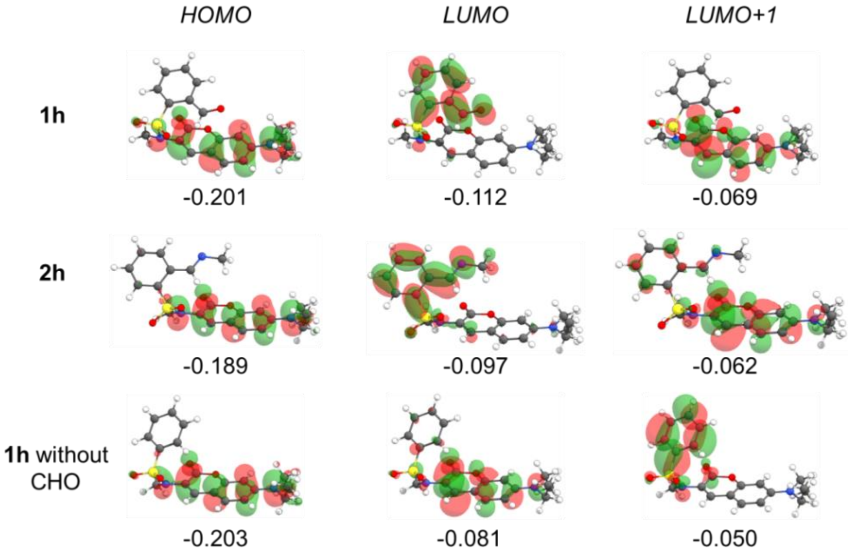

Figure S90. Selected molecular orbitals for emissions of **1h**, **2h**, and the control of **1h** without formyl group, with the orbital energy listed.

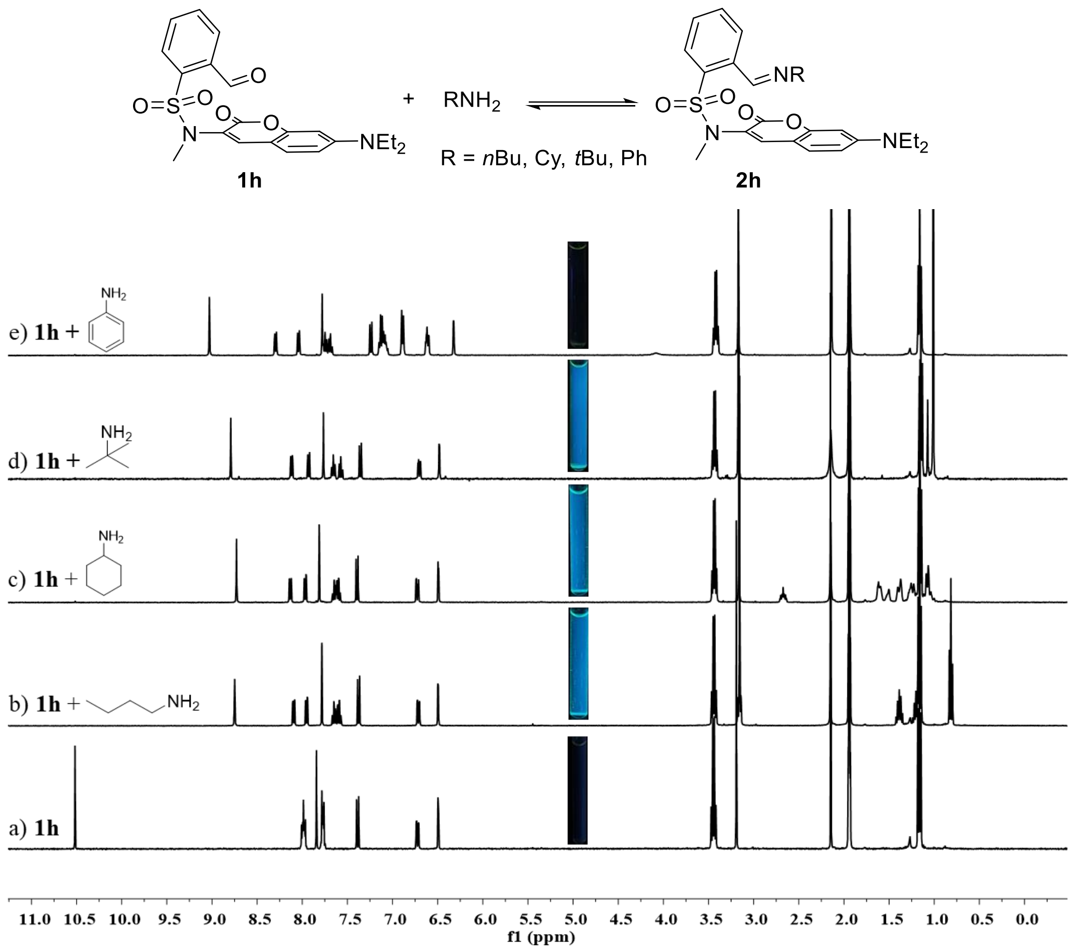

Figure S91. ^{1}H NMR spectra of **1h** (a) and its reaction with 1-butylamine (1.2 equiv, b), cyclohexylamine (1.2 equiv, c), *t*-butylamine (1.2 equiv, d), and aniline (1.2 equiv, e) in CD_3CN, with fluorescence photographs under a 365 nm UV lamp shown.

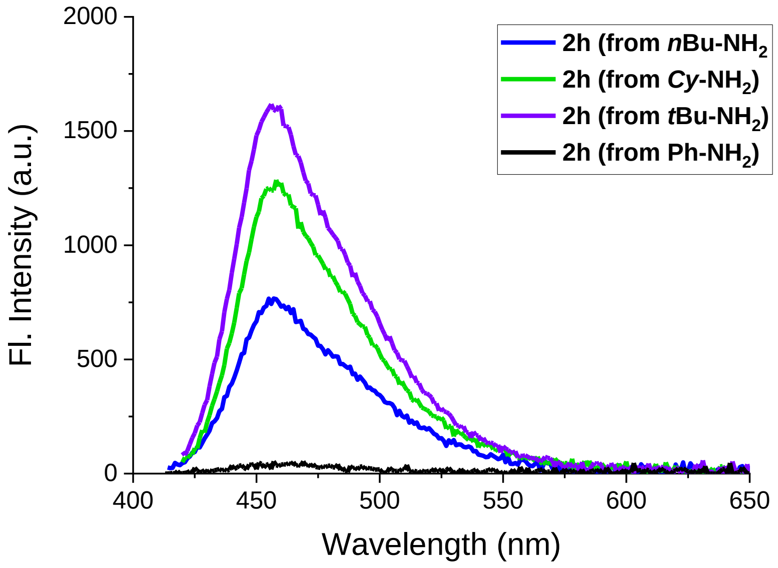

Figure S92. Fluorescence spectra of **2h** in CH_3CN (λ_{ex} = 400 nm).

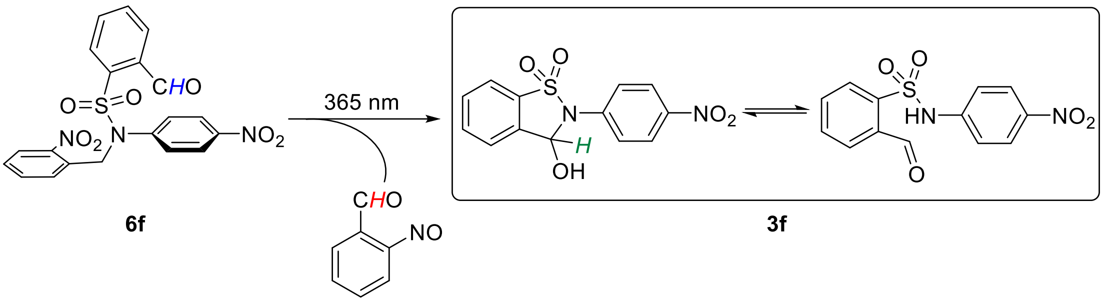

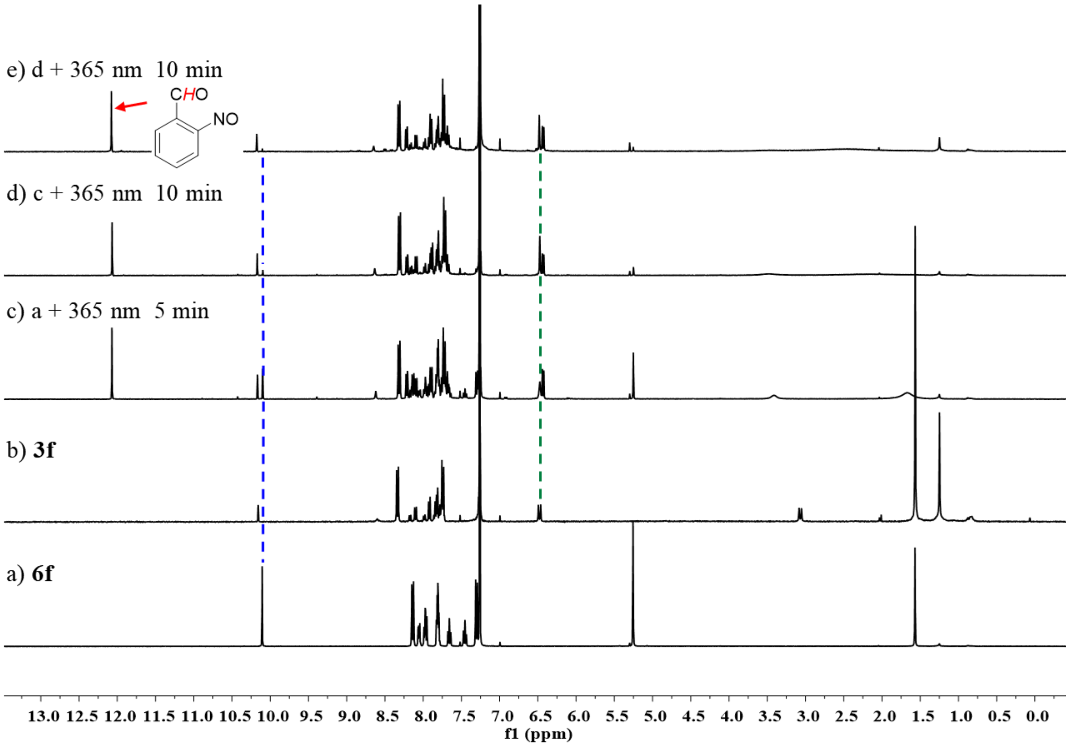

Figure S93. ^{1}H-NMR spectra of **6f** (12 mM, a), **3f** (12 mM, b), and **6f** after 5, 15, and 25 min of irradiation with UV light (365 nm) in $CDCl_3$ (c, d, e). The yield of **3f** is 91% after 25 min.

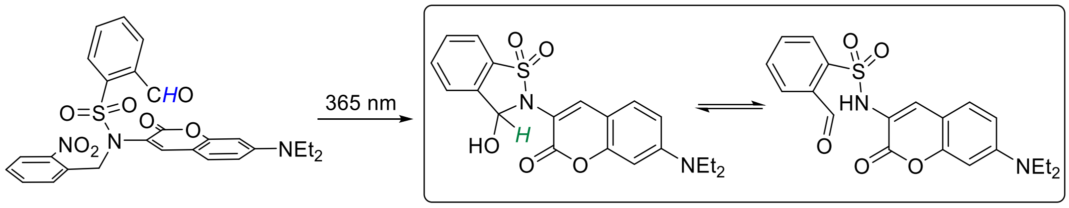

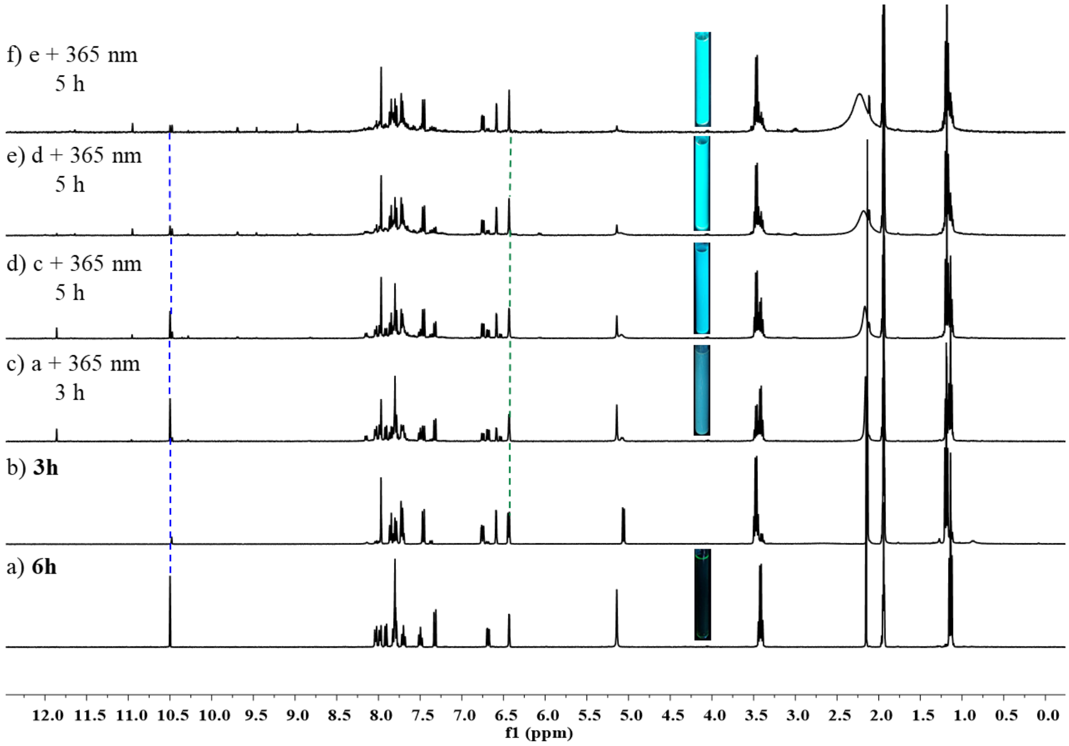

Figure S94. ^{1}H-NMR spectra of **6h** (12 mM, a), **3h** (12 mM, b), and **6h** after 3, 8, 13 and 18 h of irradiation with UV light (365 nm) in CD_3CN (c, d, e, f), with associated fluorescence photographs under a 365 nm UV lamp shown. The yield of **3h** is 87% after 18 h.

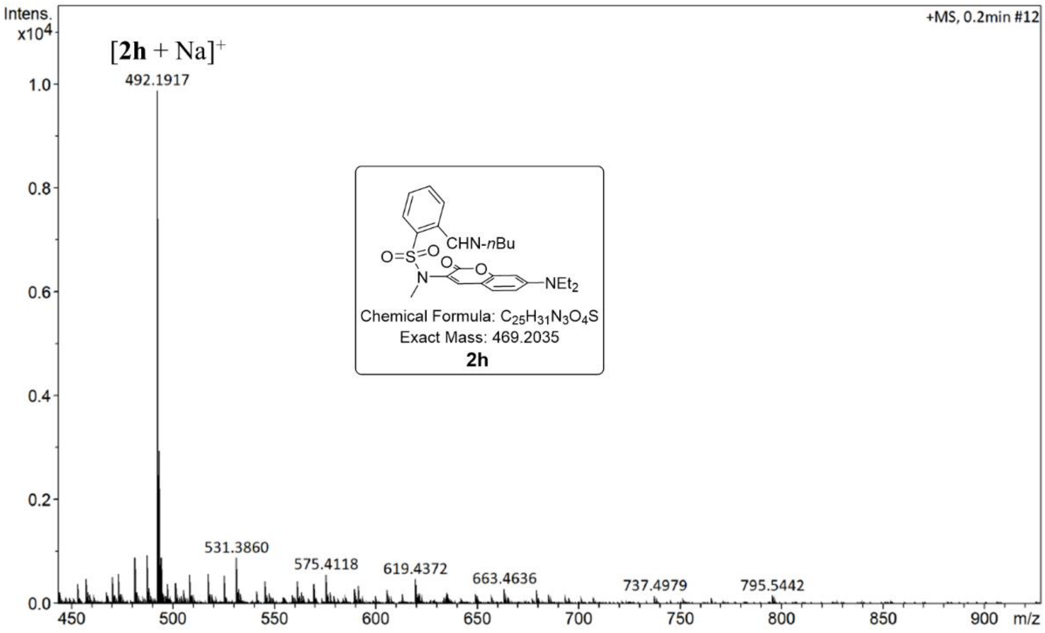

Figure S95. ESI-MS spectrum of the reaction of **1h** and 1-butylamine in CD3CN.

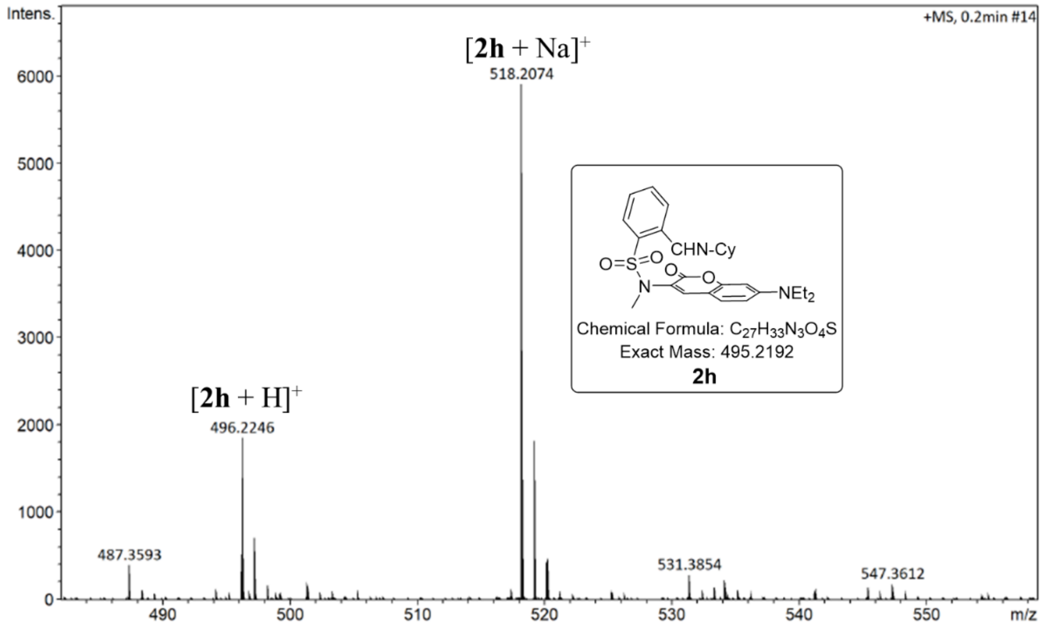

Figure S96. ESI-MS spectrum of the reaction of **1h** and cyclohexylamine in CD3CN.

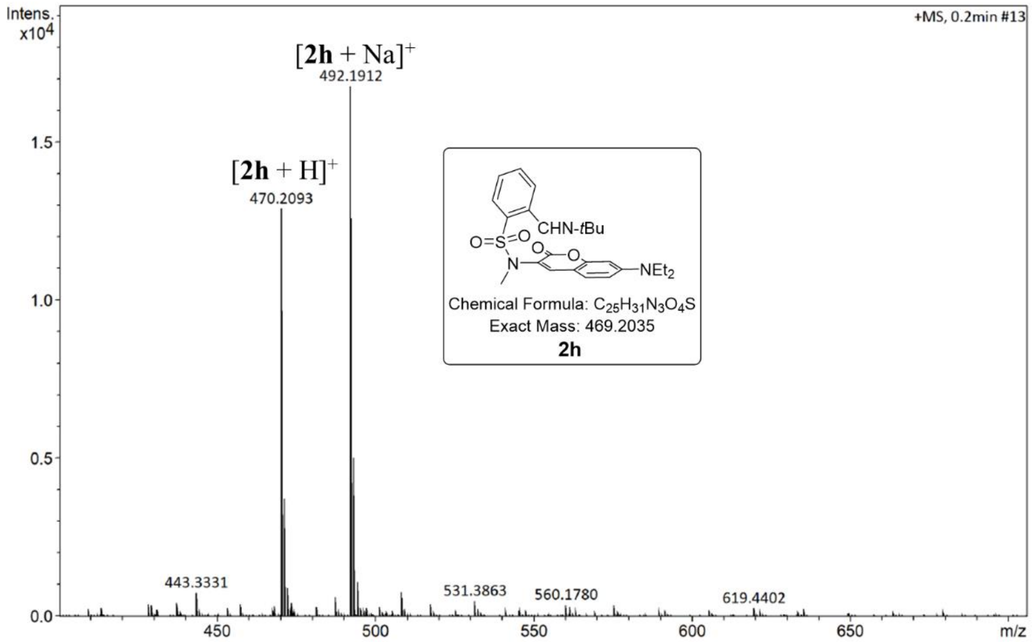

Figure S97. ESI-MS spectrum of the reaction of **1h** and *t*-butylamine in CD3CN.

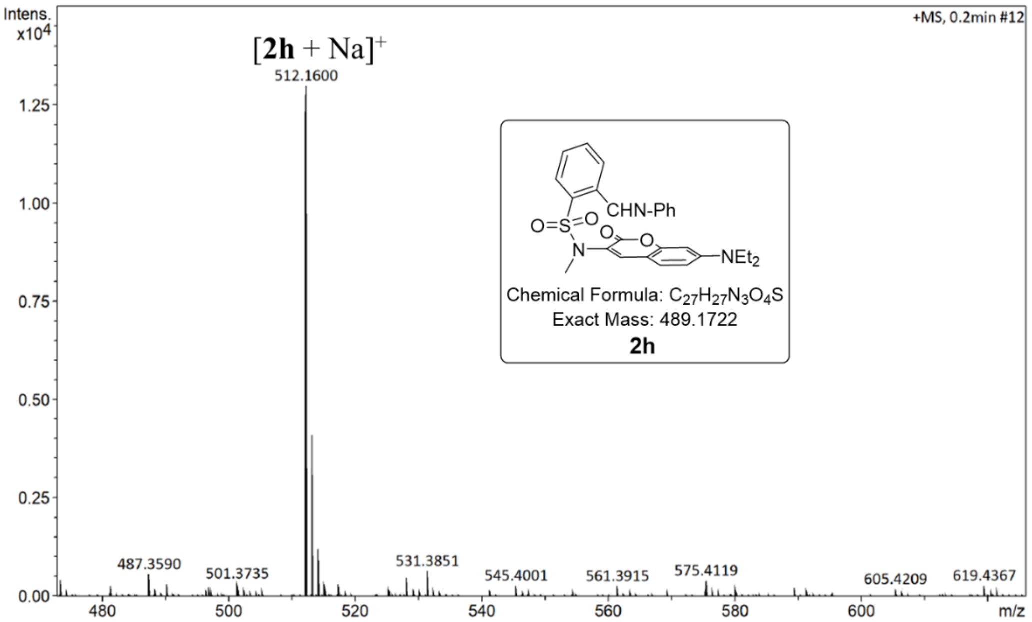

Figure S98. ESI-MS spectrum of the reaction of **1h** and aniline in CD3CN.

8. Molecular Coordinates

1a(ON) (acetonitrile)

Imaginary frequency: 0

G = -1220.131005 hartree

S	0.62216500	-1.51551500	-0.69992000
N	-0.51849000	-1.64757000	0.50213900
C	-1.57743000	-0.67899200	0.50589800
C	1.56482800	-0.06045400	-0.27125400
C	-2.60847900	-0.79364700	-0.42392100
H	-2.59633900	-1.61297300	1.12718000
C	-1.58332700	0.36683400	1.42779000
H	-0.78395600	0.46272300	2.14844400
C	-2.61615000	1.29598000	1.41290100
H	-2.61413500	2.10695600	2.12885200
C	-3.63578700	1.19378800	0.47430900
C	-3.62885300	0.14715800	-0.44275000
H	-4.42448800	0.05691700	-1.17025000
C	1.11679300	1.25752700	-0.49144300
C	2.75545200	-0.29473700	0.41006800
H	3.08285500	-1.31056800	0.57017600
O	-0.06395800	-1.29326400	-1.94475300
O	1.48862900	-2.65711700	-0.57022700
C	1.90347300	2.30716900	-0.01250300
H	1.55681500	3.31586500	-0.18769700
C	-0.13138600	1.62587500	-1.21708700
H	-0.68655500	0.81298900	-1.69260200
O	-0.52069000	2.77272600	-1.30188200
C	3.52006400	0.76944100	0.86959600
H	4.44828500	0.57406500	1.38899600
C	3.09126500	2.07406500	0.66044500
H	3.68259000	2.90598000	1.01800700
C	-0.01589600	-2.10171500	1.80982300
H	-0.88061300	-2.30964500	2.43479000
H	0.61268000	-1.35780700	2.30508400
H	0.55311700	-3.01483700	1.67138900
H	-4.43470600	1.92304900	0.45939700

1a(OFF-1) (acetonitrile)

Imaginary frequency: 0

G = -1220.129714 hartree

S	0.24543800	-1.31134800	-0.88298600
N	-0.75516000	-1.39438900	0.43579800
C	-1.83405600	-0.44286600	0.48370200
C	1.12122200	0.23708800	-0.69644200
C	-2.99205700	-0.69616200	-0.24414200
H	-3.05823400	-1.59973600	-0.83240700
C	-1.72805400	0.71405100	1.25167600
H	-0.82599000	0.91583100	1.81218000
C	-2.78491500	1.61522600	1.29089700
H	-2.69989700	2.51290300	1.88883100
C	-3.94025100	1.36980500	0.55724800
C	-4.04073000	0.21381200	-0.21055200
H	-4.93967000	0.01521900	-0.77899600
C	2.26201800	0.41784400	0.11095400
C	0.52484900	1.32866600	-1.32220000
H	-0.34967700	1.17457300	-1.93494200
O	-0.58802200	-1.18775100	-2.04779800
O	1.16250100	-2.41795400	-0.78928900
C	2.76796000	1.71271800	0.25606400
H	3.64731800	1.84299700	0.87084500
C	2.99232400	-0.67191500	0.81417900
H	2.65370300	-1.69545700	0.62739800
O	3.93081300	-0.45737900	1.55426300
C	1.05126800	2.60264700	-1.16229000
H	0.58003400	3.43991600	-1.65874900
C	2.17470000	2.79640700	-0.36799800
H	2.58770700	3.78762500	-0.23970300
C	-0.15202700	-1.78565900	1.71700200
H	-0.96374600	-2.04561500	2.39226600
H	0.43867700	-0.98687000	2.17278100
H	0.47367500	-2.65908300	1.56557800
H	-4.76061900	2.07468600	0.58612000

1a(OFF-2) (acetonitrile)

Imaginary frequency: 0

G = -1220.129765 hartree

C	2.12736900	0.54139200	1.58178600
C	1.97796400	-0.33226600	0.38774600
H	1.67005600	1.53548300	1.52755400
O	2.72901800	0.19352400	2.57659400
C	1.16836400	-0.01301200	-0.71627900
C	2.66812900	-1.54298700	0.37058400
C	1.05809200	-0.88055300	-1.79205800
H	3.28786100	-1.78793200	1.22172500
C	2.56538900	-2.41185200	-0.70607400

H	0.41818200	-0.61899100	-2.62085200
C	1.76076800	-2.08080200	-1.78804600
H	3.11061100	-3.34577800	-0.69947300
H	1.67113500	-2.75430400	-2.62931900
C	-1.64811300	0.11617200	0.44224100
C	-1.38742800	-0.91480700	1.33793200
C	-2.70996900	0.01953200	-0.45236800
H	-0.56105400	-0.82154100	2.02862100
C	-2.18713000	-2.05101900	1.33585100
H	-2.89977400	0.82389700	-1.14876400
C	-3.50623700	-1.11917600	-0.45241600
C	-3.24519900	-2.15489800	0.43912000
H	-1.98177900	-2.85359500	2.03161500
H	-4.32928200	-1.19779200	-1.15029100
H	-3.86587000	-3.04121300	0.43503100
S	0.22276400	1.49596600	-0.79716900
N	-0.80454400	1.28613800	0.48223300
O	1.05869200	2.63068700	-0.47841400
O	-0.45947000	1.51505000	-2.07053300
C	-1.38997200	2.49702100	1.08196200
H	-1.74052300	2.22888400	2.07630200
H	-2.22719100	2.88304100	0.49673200
H	-0.62287700	3.26086700	1.16913600

1b(ON) (acetonitrile)

Imaginary frequency: 0

G = -1334.681545 hartree

S	1.72950800	-1.33474900	-0.55530500
N	0.61000800	-1.79168400	0.58429400
C	-0.71438600	-1.25750900	0.44021700
O	-4.55043900	0.20994000	-0.11918000
C	2.04135700	0.39147400	-0.22218800
C	-1.57369100	-1.79747400	-0.51837600
H	-1.23057400	-2.61483500	-1.13532800
C	-1.16045400	-0.21181500	1.24084500
H	-0.50828200	0.22003900	1.98665300
C	-2.44371300	0.30134800	1.09246100
H	-2.76125200	1.11387000	1.72732100
C	-3.28936800	-0.22630200	0.11760100
C	-2.84388100	-1.28232800	-0.68503400
H	-3.51324800	-1.69085400	-1.42988000
C	1.16197000	1.43077200	-0.58679800
C	3.17197900	0.66222500	0.54303700
H	3.83248400	-0.14656100	0.81593600
O	1.12229600	-1.46935200	-1.85323100
O	2.93553200	-2.06650700	-0.26793300
C	1.46459700	2.72666000	-0.16329400
H	0.78811800	3.52007600	-0.44833100
C	-0.06745100	1.25830400	-1.41030700
H	-0.22646700	0.27559600	-1.86186800
O	-0.85825300	2.16076400	-1.59707800
C	3.45184300	1.96212600	0.94290800
H	4.33949900	2.16095900	1.52784200
C	2.59377600	2.99681300	0.59160000
H	2.80651500	4.01051100	0.90279300
C	-5.04882700	1.29520900	0.65953500
H	-6.05719000	1.48075400	0.30051200
H	-4.43958000	2.19067100	0.51916400
H	-5.08018600	1.03606000	1.72012800
C	1.12196800	-1.96203300	1.95391600
H	0.33890800	-2.44410600	2.53383700
H	1.38558100	-1.01404200	2.42943800
H	1.99609500	-2.60394000	1.92924800

1b(OFF-1) (acetonitrile)

Imaginary frequency: 0

G = -1334.679940 hartree

S	1.27147600	-1.39906300	-0.75440400
N	0.24212400	-1.66292500	0.51525800
C	-1.06697500	-1.07504200	0.40575300
O	-4.88028800	0.49331500	0.00227800
C	1.61862400	0.35575700	-0.71242000
C	-2.06475200	-1.75268700	-0.29223200
H	-1.83881200	-2.70893500	-0.74175900
C	-1.35204000	0.15495500	0.98401200
H	-0.58463600	0.69527600	1.52099300
C	-2.62119100	0.71285500	0.87804500
H	-2.81659700	1.66859000	1.33916500
C	-3.61464300	0.03547100	0.17086200
C	-3.32622000	-1.20186700	-0.41451200
H	-4.10713800	-1.71886200	-0.95563100
C	2.56423600	0.96058000	0.13957400
C	0.78686200	1.14134000	-1.50558000
H	0.06454300	0.66357400	-2.14892200
O	0.53429500	-1.65337900	-1.96277800
O	2.47310400	-2.15077800	-0.49468900
C	2.63825800	2.35623100	0.15773600
H	3.37068200	2.81284600	0.80837000

C	3.51544600	0.22469200	1.01603900
H	3.52798300	-0.86583400	0.92591900
O	4.26120500	0.78893500	1.79074800
C	0.88513800	2.52517600	-1.46971700
H	0.23508300	3.12169200	-2.09516500
C	1.81115300	3.13542300	-0.63271500
H	1.89000500	4.21350700	-0.60021600
C	-5.22760500	1.75073200	0.57678600
H	-6.27015700	1.91739000	0.32074100
H	-4.61615900	2.55505300	0.16175200
H	-5.11675500	1.73054200	1.66323800
C	0.82631000	-1.74605200	1.86005800
H	0.08027700	-2.19670800	2.51046000
H	1.09779900	-0.76809300	2.26660300
H	1.70325600	-2.38468400	1.83350400

1b(OFF-2) (acetonitrile)

Imaginary frequency: 0

G = -1334.680009 hartree

C	-2.73004800	0.41795200	1.54591700
C	-2.16740500	1.11301500	0.35802400
H	-2.76711300	-0.67628500	1.50702200
O	-3.13940900	1.01371900	2.52084900
C	-1.57739700	0.44558200	-0.72936900
C	-2.21289400	2.50594100	0.33058400
C	-1.05302700	1.15463200	-1.79912700
H	-2.66439100	3.01718200	1.16920100
C	-1.69377200	3.21946400	-0.73974500
H	-0.59261300	0.61893000	-2.61510900
C	-1.11379100	2.54399500	-1.80531500
H	-1.73975600	4.29994300	-0.74116500
H	-0.70211800	3.09236700	-2.64153600
C	0.82815100	-0.96138200	0.46546700
C	1.06514200	0.07628600	1.36511500
C	1.82838500	-1.34246800	-0.41889000
H	0.28701600	0.36927400	2.05614000
C	2.28332200	0.72870700	1.37446800
H	1.64648500	-2.14247800	-1.12260300
C	3.05721200	-0.69227400	-0.42092600
C	3.28788400	0.35089200	0.47753400
H	2.47597100	1.53699900	2.06676800
H	3.81717800	-1.00259800	-1.12134700
S	-1.44725700	-1.33161700	-0.79850300
N	-0.45400000	-1.61877500	0.49105200
O	-2.72179700	-1.94108700	-0.49263500
O	-0.84150700	-1.67427500	-2.06516700
C	-0.50272500	-2.96112400	1.09319200
H	-0.08540200	-2.88207800	2.09478200
H	0.07376300	-3.69020800	0.51971500
H	-1.53642500	-3.28758400	1.16312500
O	4.44628400	1.05078300	0.55379300
C	5.50446000	0.71210700	-0.33977700
H	5.19949100	0.84872100	-1.37957900
H	6.31910600	1.39213500	-0.10723900
H	5.83335300	-0.31808600	-0.18712100

1c(ON) (acetonitrile)

Imaginary frequency: 0

G = -1354.100762 hartree

S	1.99727100	-1.32760000	-0.57199700
N	0.94014500	-1.79107500	0.62154800
C	-0.39425900	-1.26609600	0.54341800
C	2.27631200	0.41213500	-0.28616500
C	-1.30738400	-1.81555600	-0.35303400
H	-0.99825500	-2.63766800	-0.98262600
C	-0.80651800	-0.20585600	1.34939900
H	-0.11610000	0.24581300	2.04829800
C	-2.09190800	0.29681600	1.26375500
H	-2.36374000	1.12331300	1.90098200
C	-3.02457100	-0.23890700	0.34988900
C	-2.58928500	-1.31007900	-0.46216100
H	-3.25817300	-1.75590400	-1.18129200
C	1.35215400	1.41896800	-0.62985100
C	3.42893600	0.72862600	0.42726000
H	4.12344300	-0.05625800	0.68529300
O	1.34125600	-1.50259400	-1.84168900
O	3.23519100	-2.02260300	-0.32711000
C	1.63285600	2.73016500	-0.23911200
H	0.92112900	3.49829500	-0.50715200
C	0.09567400	1.19531900	-1.39750100
H	-0.05385000	0.19939800	-1.82138800
O	-0.72765400	2.07199800	-1.56889600
C	3.68621900	2.04250900	0.79478700
H	4.59089800	2.27717400	1.33900000
C	2.78345900	3.04576200	0.46376600
H	2.97833600	4.07024400	0.75054800
C	1.52012000	-1.95294900	1.96455100
H	0.77186100	-2.44498300	2.58109200

H	1.79150200	-1.00059700	2.42708700
H	2.40172100	-2.58180200	1.90022400
N	-4.30102900	0.25413900	0.25909900
C	-4.64948600	1.47502300	0.96237000
C	-5.16695000	-0.18771000	-0.81887200
H	-4.05905400	2.33410600	0.62161700
H	-5.70041400	1.69199200	0.79382800
H	-4.50002900	1.36532100	2.03816300
H	-6.13665000	0.28985300	-0.71086300
H	-4.76392900	0.06615800	-1.80668600
H	-5.32140600	-1.26772400	-0.78213200

1c(OFF-1) (acetonitrile)

Imaginary frequency: 0

G = -1354.098840 hartree

S	1.55272100	-1.41423500	-0.74355400
N	0.54178200	-1.65920800	0.54114900
C	-0.76457100	-1.06127400	0.44835800
C	1.90949900	0.34012300	-0.73442500
C	-1.79699800	-1.75127800	-0.17603300
H	-1.60253100	-2.73307000	-0.58528500
C	-1.02458700	0.19892900	0.97952700
H	-0.23709000	0.75418800	1.47148300
C	-2.28530700	0.76218400	0.89010100
H	-2.44668800	1.73648400	1.32384500
C	-3.33990400	0.08552400	0.23873200
C	-3.06030600	-1.19734600	-0.28282800
H	-3.83454200	-1.76846200	-0.77071000
C	2.85669900	0.95403300	0.10912100
C	1.08410200	1.11723000	-1.54199600
H	0.35957300	0.63245800	-2.17750000
O	0.80002900	-1.68131500	-1.94017100
O	2.75824900	-2.16541100	-0.49480300
C	2.93814500	2.34943700	0.10561700
H	3.67164200	2.81262100	0.75041100
C	3.80186900	0.22639400	0.99807100
H	3.81284300	-0.86507000	0.91877900
O	4.54533500	0.79693900	1.77064100
C	1.18965700	2.50096000	-1.52838400
H	0.54387500	3.09081500	-2.16455300
C	2.11669200	3.12009600	-0.69896900
H	2.20070300	4.19818800	-0.68340700
C	1.14065600	-1.74822300	1.87856900
H	0.40662200	-2.21496600	2.53158800

H	1.40071700	-0.76973500	2.29170200
H	2.02657600	-2.37392200	1.83978200
N	-4.58321400	0.65153400	0.11554200
C	-4.88160700	1.89264700	0.80516200
C	-5.68692300	-0.13709300	-0.39936500
H	-4.20866700	2.69181700	0.48724100
H	-5.89597700	2.19647700	0.56290600
H	-4.80155400	1.79377500	1.89443000
H	-6.57393500	0.48785800	-0.45062300
H	-5.47555500	-0.50022900	-1.40732700
H	-5.91111200	-1.00351100	0.23438200

1c(OFF-2) (acetonitrile)

Imaginary frequency: 0

G = -1354.099806 hartree

C	-2.83308600	0.81798100	1.54990500
C	-2.23273600	1.32485700	0.28797300
H	-3.00224700	-0.26225800	1.61747500
O	-3.13324400	1.54728400	2.47271200
C	-1.77574900	0.49115600	-0.74723500
C	-2.09829800	2.70376400	0.13276500
C	-1.20534400	1.02717700	-1.89150900
H	-2.44752200	3.34249900	0.93188600
C	-1.53129600	3.24454700	-1.01171400
H	-0.84999300	0.36480100	-2.66598800
C	-1.08465400	2.40611600	-2.02466900
H	-1.43626400	4.31715900	-1.11203300
H	-0.63666600	2.81882000	-2.91827900
C	0.46791300	-1.11180500	0.49409100
C	0.87487900	-0.02601300	1.26136900
C	1.38271100	-1.70022000	-0.37459100
H	0.17029400	0.44031100	1.93672300
C	2.16209300	0.47186100	1.16748000
H	1.07699700	-2.53654500	-0.98818300
C	2.67344300	-1.21393900	-0.48153200
C	3.10518800	-0.11452600	0.29433500
H	2.43289400	1.32196400	1.77361100
H	3.34859400	-1.68989400	-1.17524300
S	-1.88463300	-1.28740200	-0.65628200
N	-0.88428700	-1.59352800	0.62162200
O	-3.21711000	-1.68768000	-0.26095200
O	-1.38685100	-1.81647200	-1.90647800
C	-1.07967000	-2.86570900	1.33473100
H	-0.61528100	-2.76237800	2.31314100

H	-0.62371100	-3.70706100	0.80813200
H	-2.14275700	-3.05000500	1.46279800
N	4.38785000	0.36098900	0.20623400
C	5.27832000	-0.15005400	-0.81938700
H	5.41697500	-1.22853000	-0.71940200
H	4.90763900	0.05326300	-1.83113700
H	6.25075700	0.32146500	-0.70990500
C	4.74827300	1.59225200	0.88451300
H	4.17669200	2.45257100	0.51634900
H	4.58416400	1.51179300	1.96104700
H	5.80433500	1.78849800	0.72312400

1d(ON) (acetonitrile)

Imaginary frequency: 0

G = -1679.771910 hartree

S	1.64548600	-1.35810100	-0.64014600
N	0.55087200	-1.79664000	0.53718900
C	-0.74626100	-1.19347300	0.47077300
C	2.05100900	0.33724000	-0.25718400
C	-1.64788700	-1.61121700	-0.50621600
H	-1.36135500	-2.39153300	-1.19471000
C	-1.11757800	-0.19628300	1.37145200
H	-0.42815300	0.13896200	2.13224500
C	-2.37396800	0.38696400	1.29314500
H	-2.66039000	1.16313900	1.98770800
C	-3.25039900	-0.02480600	0.30003400
C	-2.89908400	-1.02171600	-0.60090800
H	-3.59714100	-1.33997200	-1.36153000
C	1.20553200	1.42766100	-0.54502100
C	3.21952200	0.52549000	0.47475500
H	3.85206400	-0.32204300	0.69019900
O	0.97760700	-1.41984100	-1.91264100
O	2.82101100	-2.15817700	-0.42358600
C	1.58002700	2.68962600	-0.07921900
H	0.93038400	3.52321800	-0.30589100
C	-0.05538700	1.34697200	-1.33442100
H	-0.24347900	0.41279400	-1.87084200
O	-0.83869300	2.27137200	-1.40696000
C	3.57146100	1.79424100	0.91608900
H	4.48812800	1.92964200	1.47364300
C	2.74758000	2.87842900	0.64162300
H	3.01658700	3.86796500	0.98525000
C	1.11883600	-2.02863200	1.87633100
H	0.33546800	-2.46644400	2.48968700
H	1.47582300	-1.11148100	2.35121800
H	1.94135000	-2.73057200	1.79254300
Cl	-4.82546800	0.71897800	0.18415300

1d(OFF-1) (acetonitrile)

Imaginary frequency: 0

G = -1679.770075 hartree

S	1.19425800	-1.37776800	-0.81288900
N	0.18221000	-1.64631300	0.47699600
C	-1.09526200	-0.99239200	0.43012200
C	1.61701500	0.35588300	-0.69987100
C	-2.10463700	-1.52631700	-0.36478300
H	-1.91420100	-2.42488500	-0.93223900
C	-1.33272900	0.16352600	1.16965500
H	-0.55390000	0.59058900	1.78494000
C	-2.57288800	0.78435400	1.11855300
H	-2.76063800	1.68095600	1.69129400
C	-3.56512500	0.24594300	0.31175600
C	-3.34290300	-0.90485800	-0.43294500
H	-4.12882700	-1.31544100	-1.05021900
C	2.63020600	0.87953500	0.12787200
C	0.78043500	1.21143100	-1.41209700
H	0.00739800	0.79558700	-2.03939000
O	0.41717800	-1.54725100	-2.01017900
O	2.36130900	-2.19577800	-0.60934000
C	2.76711000	2.26845000	0.20236800
H	3.55112300	2.66315100	0.83300000
C	3.58896000	0.06562100	0.92442200
H	3.53750400	-1.02049000	0.80218800
O	4.40556500	0.56483900	1.67151100
C	0.94121900	2.58668800	-1.31950600
H	0.28699100	3.23849700	-1.88217400
C	1.93608900	3.11751700	-0.50784600
H	2.06467400	4.18861200	-0.43243000
C	0.80760000	-1.80481000	1.79738200
H	0.05092500	-2.20143200	2.46989600
H	1.18329500	-0.86456200	2.20918400
H	1.62186100	-2.51776500	1.72213200
Cl	-5.12545600	1.02810200	0.23556800

1d(OFF-2) (acetonitrile)

Imaginary frequency: 0

G = -1679.770419 hartree

C	-2.62721200	0.52737300	1.62201500

C	-2.09405700	1.17244800	0.39257600
H	-2.70055600	-0.56574600	1.61535300
O	-2.97493500	1.16192400	2.59610100
C	-1.57756500	0.45872200	-0.70309100
C	-2.09135500	2.56488700	0.33146400
C	-1.07703900	1.12227200	-1.81282600
H	-2.48630500	3.11203700	1.17592600
C	-1.59606700	3.23334000	-0.77873600
H	-0.67329100	0.55105000	-2.63467700
C	-1.08915800	2.51240400	-1.85156200
H	-1.60396800	4.31441800	-0.80543100
H	-0.69681700	3.02529300	-2.71894900
C	0.84408800	-1.01177100	0.40404700
C	1.16363200	0.04307000	1.25106300
C	1.77871100	-1.47413000	-0.51771900
H	0.43384100	0.39277000	1.96723300
C	2.41103100	0.64698800	1.17784500
H	1.52596100	-2.28995600	-1.17930700
C	3.02854600	-0.87669100	-0.60088100
C	3.33003700	0.18055600	0.24777500
H	2.66367500	1.46926800	1.83130500
H	3.75809500	-1.22575500	-1.31722900
S	-1.50862000	-1.32186700	-0.73286600
N	-0.46086000	-1.61083900	0.51778900
O	-2.78172000	-1.88350400	-0.34583100
O	-0.97144900	-1.71686400	-2.01431300
C	-0.52182100	-2.93958500	1.15137200
H	-0.03720100	-2.85787600	2.12182300
H	-0.01503400	-3.70326700	0.55778400
H	-1.56134000	-3.21932200	1.29498400
Cl	4.89947500	0.93947600	0.14263500

1e(ON) (acetonitrile)

Imaginary frequency: 0

G = -1557.333542 hartree

S	2.20233300	-1.31930200	-0.61932900
N	1.11707500	-1.79839500	0.55500500
C	-0.20912100	-1.27267000	0.46520300
C	2.51121300	0.39889400	-0.24986000
C	-1.06003300	-1.72641800	-0.54232400
H	-0.71124300	-2.47789600	-1.23332200
C	-0.65817800	-0.31433000	1.37283500
H	-0.00664600	0.04700000	2.15426300
C	-1.94226600	0.19734200	1.26514000
H	-2.28000200	0.94802600	1.96470200
C	-2.77725700	-0.23783000	0.24335900
C	-2.33694100	-1.20407900	-0.65720600
H	-2.98881600	-1.55186900	-1.44590200
C	1.60815200	1.43750600	-0.55396400
C	3.66079900	0.65790700	0.49005600
H	4.33769600	-0.15107500	0.71843500
O	1.55369900	-1.43200700	-1.89772800
O	3.41379200	-2.05435200	-0.37648300
C	1.90619000	2.72175400	-0.09424400
H	1.21290300	3.51582800	-0.33321700
C	0.36381600	1.28194300	-1.35847700
H	0.24665500	0.34521700	-1.91076300
O	-0.47978100	2.15162700	-1.42766500
C	3.93691000	1.94799400	0.92372300
H	4.83978500	2.13921500	1.48735200
C	3.05518100	2.98148500	0.63458200
H	3.26476700	3.98714100	0.97261500
C	1.68478000	-1.97472200	1.90324200
H	0.91932100	-2.43766900	2.52055400
H	1.99419400	-1.03216900	2.36073400
H	2.54013100	-2.63818400	1.83803800
C	-4.17609400	0.29294200	0.13408700
F	-4.31044500	1.51448200	0.68858400
F	-4.58646100	0.39304500	-1.14809900
F	-5.07311300	-0.51246300	0.75827500

1e(OFF-1) (acetonitrile)

Imaginary frequency: 0

G = -1557.332385 hartree

S	1.81112000	-1.38116600	-0.77709100
N	0.79351500	-1.68315400	0.50589400
C	-0.52357400	-1.12395600	0.42060600
C	2.10352800	0.38034800	-0.69940200
C	-1.45918000	-1.70942200	-0.42804000
H	-1.18137800	-2.57687500	-1.00654000
C	-0.87252000	-0.00854300	1.17887800
H	-0.15055500	0.45662500	1.83371600
C	-2.15092000	0.51996300	1.08741900
H	-2.41554000	1.38837000	1.67351500
C	-3.07848300	-0.05739500	0.22728700
C	-2.73284700	-1.17356500	-0.52910300
H	-3.45530900	-1.62818500	-1.19174600
C	3.06634300	0.99650600	0.12470700

C	1.20990600	1.15431200	-1.43565200
H	0.47675900	0.66830300	-2.06049200
O	1.07308700	-1.63794400	-1.98289800
O	3.02614500	-2.10978800	-0.52569200
C	3.09528800	2.39306100	0.17115500
H	3.84135500	2.85965000	0.79872300
C	4.08021800	0.27551600	0.94282900
H	4.11992100	-0.81266900	0.83632100
C	1.26389900	2.53939400	-1.37036700
H	0.56686500	3.12735700	-1.95161400
C	2.20781900	3.16120200	-0.56249400
H	2.25329700	4.24029600	-0.50839300
C	1.41150000	-1.74980200	1.83841000
H	0.67080900	-2.15919000	2.52084900
H	1.73142900	-0.77407500	2.21260000
H	2.26393000	-2.41948400	1.79933200
C	-4.47293300	0.49077800	0.15616100
F	-4.51791500	1.81726200	0.40033900
F	-5.04346900	0.29183600	-1.05025000
F	-5.29344300	-0.09242100	1.06687400
O	4.84465100	0.85150200	1.68970100

1e(OFF-2) (acetonitrile)

Imaginary frequency: 0

G = -1557.332590 hartree

C	-3.05551400	0.67354000	1.63815000
C	-2.49179500	1.27176500	0.39852400
H	-3.21859200	-0.40985600	1.63503500
O	-3.33446800	1.33562400	2.61587100
C	-2.04114400	0.51668300	-0.69860000
C	-2.38981200	2.65989700	0.32829500
C	-1.50814000	1.13604700	-1.81877300
H	-2.73386500	3.23889900	1.17377600
C	-1.86172300	3.28465600	-0.79230800
H	-1.15600700	0.53296700	-2.64158600
C	-1.42149700	2.52329500	-1.86652100
H	-1.79231700	4.36332600	-0.82593600
H	-1.00446500	3.00185400	-2.74194700
C	0.28870000	-1.12066900	0.39429200
C	0.69145500	-0.06822300	1.20794600
C	1.17570900	-1.68236300	-0.51905100
H	-0.00622900	0.35661900	1.91484700
C	1.98028100	0.43324900	1.10540900
H	0.85295200	-2.49411000	-1.15424100
C	2.46499100	-1.18260700	-0.62404000
C	2.86293300	-0.12247300	0.18495400
H	2.29071100	1.25612300	1.73308000
H	3.15284300	-1.61140700	-1.33859200
S	-2.09447400	-1.26408500	-0.71482400
N	-1.05722000	-1.61450100	0.53172300
O	-3.39778500	-1.73607100	-0.31104900
O	-1.59366500	-1.70517600	-1.99546500
C	-1.20635800	-2.93013300	1.17936300
H	-0.70478100	-2.87527800	2.14302600
H	-0.76527400	-3.73442600	0.58724100
H	-2.26177000	-3.13007800	1.33824200
C	4.27088300	0.39266400	0.09995100
F	4.80101700	0.24144800	-1.13100900
F	5.09752200	-0.25909300	0.95578000
F	4.35839900	1.70267100	0.40987100

1f(ON) (acetonitrile)

Imaginary frequency: 0

G = -1424.731154 hartree

S	1.89075800	-1.35045500	-0.58732400
O	1.22471600	-1.50526500	-1.85147700
O	3.08565400	-2.10204000	-0.32052400
N	0.80474300	-1.75034300	0.62390500
C	-0.50977500	-1.21374800	0.52377900
C	-3.04766300	-0.16225700	0.29102000
C	-0.95668100	-0.25411300	1.43607500
H	-0.30746800	0.09727800	2.22328600
C	3.39765600	0.64200000	0.42537500
H	4.06773900	-0.16741300	0.67169300
C	1.33721700	1.41151900	-0.61217400
C	2.23455000	0.37367200	-0.28909000
C	-1.36017000	-1.65773500	-0.49272800
H	-1.01744600	-2.41508300	-1.17931300
C	-2.23028000	0.27395600	1.32342400
H	-2.58110000	1.02601900	2.01225600
C	1.65380000	2.70557000	-0.19489400
H	0.96557900	3.49975300	-0.44767600
O	-0.77873000	2.09905700	-1.43784100
N	-4.38969300	0.40620700	0.15861500
C	3.69379800	1.94229000	0.81357300
H	4.60731900	2.14140900	1.35684900
C	0.08422500	1.24698400	-1.40133900
H	-0.01896000	0.32112800	-1.97524100

C	2.81729300	2.97507300	0.50757700
H	3.04188300	3.98828000	0.81162500
C	-2.62994200	-1.12604000	-0.61746000
H	-3.29502100	-1.45792100	-1.39902000
O	-4.75144500	1.23118400	0.98765600
C	1.38960200	-1.87181000	1.97130200
H	2.23037000	-2.55491600	1.92389100
H	1.72544200	-0.91450300	2.37599900
H	0.62640300	-2.28967600	2.62247000
O	-5.08569300	0.03077800	-0.77580500

1f(OFF-1) (acetonitrile)

Imaginary frequency: 0

G = -1424.729874 hartree

S	1.46388800	-1.41390200	-0.73259200
O	0.71471800	-1.69730600	-1.92457100
O	2.65436900	-2.16901500	-0.45031000
N	0.44010800	-1.63045600	0.57143300
C	-0.85649400	-1.04273000	0.47793500
C	-3.36858200	0.07466500	0.28764600
C	-1.20351900	0.03901200	1.28969400
H	-0.48960500	0.45385900	1.98486800
C	0.91886100	1.10973300	-1.47096400
H	0.16433600	0.62191400	-2.06840900
C	2.79574500	0.95854500	0.06677700
C	1.80339900	0.33974000	-0.71939600
C	-1.78624700	-1.56652200	-0.42243500
H	-1.51650800	-2.41321800	-1.03296300
C	-2.46525400	0.60014300	1.20031700
H	-2.74311700	1.44073000	1.81641100
C	2.86264700	2.35446200	0.06005800
H	3.63103800	2.82324900	0.65832700
O	4.59733200	0.82579500	1.60549400
N	-4.70128600	0.66951300	0.18389500
C	1.01076600	2.49427400	-1.45763100
H	0.32081500	3.07925500	-2.05015200
C	3.80367700	0.24179900	0.89667400
H	3.81063700	-0.85051700	0.83552100
C	1.98413400	3.11903300	-0.68795500
H	2.05946300	4.19770400	-0.67478300
C	-3.04459400	-1.00346500	-0.52641600
H	-3.77386400	-1.39992300	-1.21520500
O	-4.97989900	1.59736800	0.93284800
C	1.07507700	-1.66571200	1.89780300
H	1.89762400	-2.37204700	1.87339700
H	1.44230300	-0.68949700	2.22337100
H	0.33016700	-2.01495200	2.60825000
O	-5.47463800	0.21248500	-0.64809800

1f(OFF-2) (acetonitrile)

Imaginary frequency: 0

G = -1424.729432 hartree

C	-2.83038600	0.64881000	1.57864600
C	-2.26150900	1.23139400	0.33361400
H	-2.94668600	-0.44022500	1.60787200
O	-3.16205900	1.32908800	2.52674600
C	-1.75710300	0.46294600	-0.73057200
C	-2.21182100	2.61967700	0.22328000
C	-1.22370600	1.06945300	-1.85769400
H	-2.59712800	3.20935000	1.04318900
C	-1.68329100	3.23151900	-0.90430900
H	-0.83016300	0.45688400	-2.65432100
C	-1.19001300	2.45706600	-1.94549300
H	-1.65508000	4.31062200	-0.96895000
H	-0.77254700	2.92573900	-2.82600400
C	0.61800300	-1.05168800	0.44985900
C	0.96930600	0.04376100	1.23282500
C	1.53825100	-1.61783000	-0.43005300
H	0.24578600	0.46638800	1.91410800
C	2.23916800	0.58828800	1.13528100
H	1.25282800	-2.46341700	-1.03784600
C	2.81081500	-1.08205200	-0.53656300
C	3.13879300	0.01555700	0.24762700
H	2.52975200	1.43951900	1.73035500
H	3.53769900	-1.49983600	-1.21510800
S	-1.73810500	-1.31753700	-0.69333200
N	-0.71042000	-1.58605500	0.58482500
O	-3.02649500	-1.83231200	-0.29673100
O	-1.19032200	-1.77731400	-1.94734000
C	-0.82123800	-2.88464100	1.27497300
H	-0.33925500	-2.77873000	2.24430200
H	-0.34121400	-3.69117200	0.71748500
H	-1.87147200	-3.11699400	1.42306700
N	4.48494300	0.59139900	0.13280100
O	4.75553200	1.56381800	0.82356500
O	5.26883000	0.07065500	-0.64833300

2a(ON) (acetonitrile)

Imaginary frequency: 0

G = -1239.537330 hartree

S	1.11908000	-1.26645300	-0.93725400
N	0.18835000	-1.94882700	0.26388600
C	-1.07662900	-1.32504600	0.53475300
C	1.62002500	0.31628300	-0.28317300
C	-2.13407500	-1.51428400	-0.35195600
H	-1.98554500	-2.12965600	-1.22665800
C	-1.25436500	-0.54019700	1.67202700
H	-0.43567800	-0.38316500	2.35940400
C	-2.48481700	0.05651100	1.91552100
H	-2.61525100	0.66891000	2.79781300
C	-3.53663500	-0.11919400	1.02467400
C	-3.35725100	-0.90489200	-0.10872000
H	-4.17294500	-1.04917100	-0.80466900
C	0.79396500	1.45843700	-0.26641400
C	2.87019900	0.32944800	0.33633000
H	3.48182800	-0.55921100	0.30765600
O	0.28358600	-1.07946000	-2.09424100
O	2.29340100	-2.09210500	-1.05681700
C	1.28438500	2.59492300	0.38551500
H	0.65959300	3.47613900	0.39071800
C	-0.53556900	1.54019100	-0.91091200
H	-0.78703000	0.76381300	-1.62956400
C	3.32606500	1.47272100	0.97299800
H	4.30065100	1.47422200	1.44163300
C	2.52489500	2.60840300	0.99934200
H	2.87133400	3.50758300	1.49124700
C	0.93295200	-2.47651000	1.41898000
H	0.22699200	-3.02861400	2.03442600
H	1.39508600	-1.69189600	2.02320000
H	1.70308400	-3.15261000	1.06390100
N	-1.33906800	2.48262100	-0.64220500
C	-2.60354100	2.50890100	-1.34657200
H	-3.41854300	2.50838400	-0.62050900
H	-2.67702600	3.44236400	-1.91019500
H	-2.73885600	1.66413000	-2.03179600
H	-4.49146000	0.35382100	1.21180100

2a(OFF-1) (acetonitrile)

Imaginary frequency: 0

G = -1239.536263 hartree

S	0.01560200	-0.97637100	-1.29536800
N	-0.74910100	-1.47727400	0.08798400
C	-1.89054800	-0.70194100	0.49800100
C	0.71304100	0.62094900	-0.90217500
C	-3.13187400	-0.97510000	-0.06632400
H	-3.21394000	-1.76090400	-0.80329100
C	-1.76016400	0.30695500	1.44912500
H	-0.79318000	0.52696800	1.87959900
C	-2.87589000	1.03848700	1.83686300
H	-2.77164200	1.82198700	2.57573000
C	-4.11741500	0.77195400	1.26985300
C	-4.24272400	-0.23435800	0.31726600
H	-5.20723800	-0.44774800	-0.12423500
C	1.89219700	0.79862600	-0.15249200
C	-0.06693900	1.71588300	-1.27277000
H	-0.97171400	1.55060600	-1.83671600
O	-1.00424800	-0.76055800	-2.28764400
O	1.06050100	-1.93141200	-1.56709400
C	2.24750000	2.10696300	0.19232600
H	3.15822200	2.24797500	0.75626100
C	2.78102400	-0.30450000	0.27188000
H	2.70686100	-1.23987700	-0.28063800
C	0.31292900	3.00018200	-0.91731800
H	-0.29655700	3.84198100	-1.21632700
C	1.47400500	3.19384000	-0.17818900
H	1.78075100	4.19265900	0.10242900
C	0.07081200	-2.06496700	1.15672800
H	-0.59832200	-2.61946700	1.81120200
H	0.60215000	-1.31611200	1.74730800
H	0.78677000	-2.75284200	0.71905600
N	3.59731400	-0.16031000	1.23072900
C	4.44661700	-1.28414900	1.56532000
H	5.49247600	-0.97811000	1.48686100
H	4.27593400	-1.56264400	2.60791000
H	4.28151700	-2.16151200	0.92851000
H	-4.98466400	1.34489100	1.57057700

2a(OFF-2) (acetonitrile)

Imaginary frequency: 0

G = -1239.536662 hartree

C	-2.16570900	-0.77771300	0.55283300
C	-1.78110900	0.59057000	0.14952300
H	-2.08850400	-1.55915700	-0.20450900
C	-0.84061600	0.85117900	-0.86103900
C	-2.35551200	1.68417600	0.79751100
C	-0.48725400	2.15098700	-1.20076100

H	-3.08142000	1.49241300	1.57470300
C	-2.01247600	2.98481000	0.45680700
H	0.25668000	2.31334700	-1.96583200
C	-1.07895100	3.22193000	-0.54456900
H	-2.47636200	3.81443500	0.97352000
H	-0.80567000	4.23339400	-0.81231200
C	1.62749100	-0.54285900	0.34630800
C	1.25674000	-0.20420300	1.64349900
C	2.88610100	-0.19976600	-0.13860100
H	0.27504000	-0.47897800	2.00327600
C	2.14709900	0.48521700	2.45755100
H	3.15950300	-0.46406700	-1.15024600
C	3.77215500	0.49224900	0.67830300
C	3.40379700	0.83590700	1.97517400
H	1.85753600	0.75081000	3.46567000
H	4.74942700	0.76348600	0.30126100
H	4.09529800	1.37597300	2.60851700
S	-0.01291600	-0.46748600	-1.72009500
N	0.68915800	-1.27438900	-0.46518100
O	-0.97506300	-1.37367900	-2.30694000
O	0.95435700	0.13232000	-2.61123900
C	0.89682900	-2.72398900	-0.58436200
H	0.96809500	-3.12825300	0.42353700
H	1.80985800	-2.96378600	-1.13375600
H	0.04306600	-3.16934200	-1.08606300
N	-2.57242000	-1.02492500	1.72658800
C	-2.94508900	-2.38936500	2.03839900
H	-3.98323600	-2.40768600	2.37781800
H	-2.83202700	-3.07672200	1.19155100
H	-2.33311300	-2.74479600	2.87049800

H	2.52946500	0.46675700	-2.18012100
C	3.05466800	-0.61803100	-0.38835000
C	2.58747400	-1.48764400	0.60099400
H	3.26758400	-1.81024200	1.37755000
C	-1.04654300	1.50230900	0.38204700
C	-3.15414900	0.83442100	-0.64777300
H	-3.90955900	0.07934000	-0.80267100
O	-1.31039900	-1.20686400	2.00236100
O	-3.26279600	-1.74487700	0.57460600
C	-1.20449000	2.75120400	-0.22850500
H	-0.44057900	3.49430900	-0.05141900
C	0.14388900	1.28562400	1.23361900
H	0.11274900	0.44680400	1.92501000
C	-3.28095500	2.08262200	-1.23602900
H	-4.14342200	2.30195900	-1.85047800
C	-2.29712000	3.04230200	-1.02684300
H	-2.38674700	4.02094200	-1.47938100
C	4.86742100	0.68438200	-1.24028600
H	5.89876400	0.86896100	-0.95311300
H	4.31082800	1.62404000	-1.22969400
H	4.83870900	0.25384300	-2.24365500
C	-1.59043000	-2.23005400	-1.68177900
H	-0.89275500	-2.86113200	-2.22692200
H	-1.80903000	-1.34184400	-2.28024000
H	-2.51051000	-2.77811000	-1.50978100
N	1.15015100	2.05310900	1.16278700
C	2.25787400	1.79549300	2.05882600
H	3.16721300	1.64348100	1.47434500
H	2.42290100	2.67763800	2.68242800
H	2.10272300	0.92349600	2.70464400

2b(ON) (acetonitrile)

Imaginary frequency: 0

G = -1354.087306 hartree

S	-1.95797700	-1.15003800	0.71758800
N	-0.96944600	-1.91067800	-0.38641400
C	0.40140700	-1.48202600	-0.40969500
O	4.34997300	-0.22782700	-0.27499600
C	-2.05360100	0.54127900	0.15844900
C	1.27588000	-1.91959600	0.58586300
H	0.91191600	-2.58695000	1.35299800
C	0.87268400	-0.63136700	-1.40202500
H	0.20888600	-0.27871700	-2.17838300
C	2.19421100	-0.20165900	-1.40232900

2b(OFF-1) (acetonitrile)

Imaginary frequency: 0

G = -1354.086360 hartree

S	0.98702700	-1.37724000	-1.05752800
N	0.11646700	-1.68765600	0.31611700
C	-1.19767100	-1.10447100	0.37529200
O	-5.03501700	0.45640800	0.46150000
C	1.27275400	0.38739700	-1.06219300
C	-2.27665400	-1.78865100	-0.18056800
H	-2.10833100	-2.74746300	-0.64985200
C	-1.40774300	0.13002200	0.97587500
H	-0.57696100	0.67529000	1.40241900
C	-2.68077800	0.68615200	1.03149600

H	-2.81640900	1.64626700	1.50487100
C	-3.75696900	0.00091500	0.46699400
C	-3.54523600	-1.24116000	-0.14029300
H	-4.38903800	-1.76334800	-0.57078400
C	2.22516400	1.02526900	-0.24425500
C	0.38804100	1.13375600	-1.83898500
H	-0.34094800	0.61968100	-2.44576600
O	0.12766800	-1.66585300	-2.17615400
O	2.23577200	-2.08915800	-0.93918600
C	2.25158200	2.42370400	-0.25144600
H	2.98963800	2.91558500	0.36557700
C	3.20251500	0.30901700	0.60300900
H	3.42258600	-0.72434600	0.33842700
C	0.43982200	2.51862500	-1.82629600
H	-0.24764000	3.08686000	-2.43789700
C	1.37421800	3.16444200	-1.02529400
H	1.42412300	4.24504500	-1.00972400
C	-5.30516600	1.72224600	1.05845100
H	-6.37227100	1.88675900	0.93793600
H	-4.75324000	2.51961400	0.55581100
H	-5.05390800	1.71775400	2.12144300
C	0.83656600	-1.83125600	1.58827000
H	0.19027200	-2.38531000	2.26603900
H	1.08543400	-0.87100400	2.04432300
H	1.74636200	-2.39941100	1.42426900
N	3.76333200	0.87861600	1.58664900
C	4.72472300	0.11065800	2.34967800
H	5.68621500	0.62933300	2.33931600
H	4.40006800	0.06701200	3.39198900
H	4.86461900	-0.91037600	1.97498500

2b(OFF-2) (acetonitrile)

Imaginary frequency: 0

G = -1354.086520 hartree

C	-2.57359400	0.27284200	1.13106800
C	-2.02782100	1.08220700	0.02211700
H	-2.90196600	-0.73977500	0.89234200
C	-1.39642700	0.51381500	-1.09667100
C	-2.12158000	2.47294400	0.07661300
C	-0.87672600	1.30647600	-2.11215500
H	-2.60706400	2.91773900	0.93348300
C	-1.61112300	3.26934100	-0.93850600
H	-0.37717200	0.83667900	-2.94575600
C	-0.98914400	2.68828900	-2.03672100

H	-1.70066900	4.34546800	-0.87223800
H	-0.58694100	3.30414400	-2.82943700
C	0.93338200	-0.93924400	0.29261700
C	1.05819700	0.07813100	1.23764500
C	2.04014300	-1.32059300	-0.45369000
H	0.19548800	0.37291000	1.81862200
C	2.27384600	0.70795800	1.42770600
H	1.94335100	-2.10541700	-1.19066900
C	3.26759800	-0.69287500	-0.27283200
C	3.38730100	0.32820700	0.67122600
H	2.38147700	1.50011300	2.15631400
H	4.11251200	-1.00374400	-0.86775400
S	-1.18286200	-1.24576300	-1.24573800
N	-0.34238500	-1.58399100	0.13105300
O	-2.45710400	-1.92126200	-1.13299200
O	-0.41385200	-1.49108300	-2.44537200
C	-0.52148000	-2.89775500	0.76294900
H	-0.24605000	-2.79651900	1.81095000
H	0.10375100	-3.66428900	0.29956200
H	-1.56479900	-3.19161700	0.69666800
O	4.53576700	1.00445500	0.92301300
C	5.70308200	0.65883200	0.18134400
H	5.55121100	0.81895400	-0.88833900
H	6.48919400	1.31756500	0.53963900
H	5.98816200	-0.38054600	0.35862200
N	-2.66014600	0.74200300	2.30443300
C	-3.22170600	-0.11062500	3.33186100
H	-4.08870300	0.38556200	3.77392100
H	-3.52298100	-1.09807400	2.96223400
H	-2.48745900	-0.23761400	4.13061300

2c(ON) (acetonitrile)

Imaginary frequency: 0

G = -1373.506733 hartree

S	2.14536700	-1.22087600	-0.71359200
N	1.16151900	-1.91814600	0.43320100
C	-0.19347400	-1.44211700	0.47961200
C	2.32642400	0.47919100	-0.20319000
C	-1.11663400	-1.88668600	-0.46255800
H	-0.80506200	-2.60580800	-1.20660100
C	-0.61443200	-0.53585400	1.44962200
H	0.07780400	-0.18208200	2.20099300
C	-1.91267500	-0.05912800	1.46265400
H	-2.19492000	0.64401300	2.23017700

C	-2.84213900	-0.45350700	0.47807800
C	-2.41331700	-1.40787100	-0.46899100
H	-3.09310200	-1.77209100	-1.22304600
C	1.35050800	1.47423200	-0.41633200
C	3.46881700	0.74995300	0.55137300
H	4.19850500	-0.03125900	0.69981200
O	1.46077500	-1.27980500	-1.97952800
O	3.43151200	-1.86264600	-0.59671800
C	1.58353100	2.73286500	0.14942000
H	0.84238300	3.50068700	-0.01890600
C	0.11535700	1.28029600	-1.20749900
H	0.06535500	0.39917400	-1.84243200
C	3.66966300	2.00801000	1.09592400
H	4.56387300	2.20879300	1.67011300
C	2.71802300	3.00169800	0.89490500
H	2.86496000	3.98849300	1.31361900
C	1.80499100	-2.22648900	1.71972100
H	1.10449900	-2.82811700	2.29398400
H	2.05810900	-1.33149300	2.29431400
H	2.70785800	-2.80048000	1.54025300
N	-0.83958400	2.11184000	-1.14585600
C	-2.00525100	1.86540000	-1.96790500
H	-2.88413500	1.77916900	-1.32532800
H	-2.16818100	2.72514400	-2.62251400
H	-1.92456100	0.95841300	-2.57811800
N	-4.10424000	0.08363600	0.42861300
C	-5.08841300	-0.47114200	-0.48146100
C	-4.56155500	0.94895100	1.49911500
H	-5.31876700	-1.51984800	-0.25852200
H	-6.00604000	0.10498100	-0.40350000
H	-4.74379200	-0.41202400	-1.51588700
H	-5.55575700	1.31435100	1.25799400
H	-4.60838600	0.43099600	2.46471400
H	-3.90522500	1.81446800	1.60969500

2c(OFF-1) (acetonitrile)

Imaginary frequency: 0

G = -1373.505424 hartree

S	1.30179700	-1.42065100	-1.02366700
N	0.41540600	-1.69088400	0.34408400
C	-0.89190000	-1.09010100	0.38029300
C	1.57575900	0.34582800	-1.09193800
C	-1.98600100	-1.77833100	-0.13027300
H	-1.83848200	-2.76332300	-0.55154500
C	-1.09070800	0.17543200	0.92539600
H	-0.25297400	0.73008800	1.32704000
C	-2.35118700	0.74496400	0.96150900
H	-2.46266900	1.72413600	1.40009900
C	-3.47037900	0.06863400	0.42797100
C	-3.25170700	-1.21890400	-0.11026300
H	-4.07448600	-1.78918500	-0.51208400
C	2.49041900	1.02194000	-0.26225800
C	0.72228800	1.05476900	-1.93484700
H	0.02035900	0.51187500	-2.54835100
O	0.46431300	-1.75396800	-2.14720700
O	2.55695800	-2.11616700	-0.86765600
C	2.51311300	2.41885300	-0.32546600
H	3.22275300	2.93960900	0.30114600
C	3.42992100	0.34331800	0.65503700
H	3.66330700	-0.69882900	0.44033900
C	0.76858600	2.43946400	-1.97705500
H	0.10480100	2.97863700	-2.63911000
C	1.66622500	3.12270100	-1.16517000
H	1.71127400	4.20331300	-1.19182500
C	1.11113300	-1.84736800	1.62750700
H	0.45761600	-2.41985800	2.28314400
H	1.33615600	-0.89071200	2.10315000
H	2.03206500	-2.40097700	1.47596900
N	3.94583600	0.95157500	1.64019800
C	4.87297300	0.21530000	2.47369300
H	5.83369100	0.73536600	2.48643200
H	4.50240000	0.21005500	3.50150300
H	5.03009200	-0.81882700	2.14436000
N	-4.71758200	0.64070100	0.42943700
C	-4.93967600	1.88161400	1.14771800
C	-5.87119400	-0.14499900	0.03352900
H	-4.30254100	2.68021500	0.76189100
H	-5.97337300	2.18752500	1.01384900
H	-4.74578800	1.78124800	2.22247700
H	-6.75559100	0.48502300	0.06489300
H	-5.76271600	-0.51690300	-0.98739500
H	-6.03642400	-1.00540600	0.69326900

2c(OFF-2) (acetonitrile)

Imaginary frequency: 0

G = -1373.505217 hartree

C	-2.69865000	0.85334900	1.00612500
C	-2.12086800	1.18391100	-0.31267600

H	-3.13098700	-0.14109800	1.12343300
C	-1.61774600	0.20867700	-1.18978200
C	-2.04912400	2.51757000	-0.71613700
C	-1.06157400	0.55791400	-2.41382900
H	-2.43526100	3.27341400	-0.04729600
C	-1.50114200	2.87134700	-1.94058700
H	-0.66400500	-0.21592700	-3.05276500
C	-1.00728900	1.89214000	-2.79382900
H	-1.46120900	3.91321500	-2.22944300
H	-0.57630900	2.16196200	-3.74828200
C	0.60132900	-1.00922500	0.54370300
C	0.90188400	0.23969900	1.07665300
C	1.61896900	-1.74680700	-0.05420600
H	0.11587200	0.82236400	1.53740400
C	2.18672200	0.74967000	1.01563000
H	1.39557100	-2.71330500	-0.48516600
C	2.90882500	-1.25058700	-0.12396600
C	3.23353700	0.01303300	0.41846200
H	2.37500500	1.72680000	1.43196400
H	3.66672500	-1.84797600	-0.60605800
S	-1.62357800	-1.51884700	-0.76187100
N	-0.74588600	-1.50684900	0.63265300
O	-2.96185900	-1.94759500	-0.41468600
O	-0.96722200	-2.23445300	-1.83423400
C	-1.02261800	-2.54074900	1.63847200
H	-0.67709000	-2.16183300	2.59838800
H	-0.50717500	-3.47813100	1.41668800
H	-2.09274400	-2.71947900	1.69091600
N	-2.69479600	1.69350200	1.95427400
C	-3.29507200	1.29457200	3.21055200
H	-4.09419200	1.99505800	3.46378600
H	-3.70198300	0.27637300	3.19538300
H	-2.54660100	1.36756900	4.00288100
N	4.51428000	0.50264000	0.37102000
C	5.52323200	-0.19290700	-0.40620000
H	5.65825900	-1.21627400	-0.05049500
H	5.27289300	-0.23358600	-1.47317700
H	6.47206000	0.32455400	-0.29649600
C	4.77581700	1.87325400	0.76936100
H	4.24681100	2.59845400	0.13927000
H	4.47960200	2.04550700	1.80605800
H	5.84207700	2.06604400	0.69218600

2d(ON) (acetonitrile)

Imaginary frequency: 0

G = -1699.178374 hartree

S	-1.84783800	-1.18963600	0.82572600
N	-0.85753100	-1.93265800	-0.29421100
C	0.48048400	-1.43094000	-0.39683800
C	-2.09598800	0.45454700	0.18272400
C	1.40279900	-1.73194800	0.60282500
H	1.10024000	-2.34685900	1.43669500
C	0.86896800	-0.64684000	-1.48102300
H	0.16265000	-0.39992100	-2.25996000
C	2.16384500	-0.15610400	-1.56256700
H	2.46394700	0.45989300	-2.39771200
C	3.06327100	-0.44983500	-0.54866700
C	2.69556200	-1.23596200	0.53440300
H	3.41015500	-1.46109700	1.31263300
C	-1.15218800	1.49493100	0.30254100
C	-3.25274700	0.62222200	-0.57927200
H	-3.95761400	-0.19162000	-0.65385900
O	-1.13445900	-1.13112500	2.07410600
O	-3.10600900	-1.88792900	0.77597800
C	-1.42970600	2.69197900	-0.36604500
H	-0.71537000	3.49641300	-0.26772700
C	0.08660900	1.41637900	1.10833600
H	0.13816600	0.63534600	1.86372600
C	-3.49804800	1.82264500	-1.22661500
H	-4.40281000	1.94529400	-1.80607100
C	-2.57755700	2.85892500	-1.12156100
H	-2.75963400	3.80116900	-1.62108100
C	-1.51545500	-2.34278300	-1.54581900
H	-0.80251400	-2.94319200	-2.10543500
H	-1.82901500	-1.49497300	-2.15997900
H	-2.38236300	-2.94856500	-1.30576700
Cl	4.68715500	0.18848600	-0.63128100
N	1.03648600	2.23652300	0.93165800
C	2.19326800	2.12449700	1.79471500
H	3.09070200	2.01677200	1.18292100
H	2.30413900	3.05290100	2.36066400
H	2.13545300	1.28345000	2.49549300

2d(OFF-1) (acetonitrile)

Imaginary frequency: 0

G = -1699.177551 hartree

S	-0.90781000	-1.28017400	1.14969100
N	-0.05515700	-1.65966200	-0.22525300

C	1.22938000	-1.03134100	-0.35947100
C	-1.26875800	0.46324800	1.00975300
C	2.32767700	-1.58442200	0.29001600
H	2.19790500	-2.47748500	0.88329800
C	1.38369200	0.12088600	-1.12616400
H	0.53299200	0.56495400	-1.62342000
C	2.63048600	0.71758300	-1.24917200
H	2.75454500	1.61196100	-1.84236400
C	3.71486900	0.15727300	-0.58851000
C	3.57634100	-0.98894200	0.18292000
H	4.43229100	-1.41415200	0.68672500
C	-2.28920100	0.98896100	0.19367100
C	-0.37083700	1.30809500	1.66156800
H	0.40901300	0.87829600	2.27070600
O	-0.00723200	-1.43981700	2.26062100
O	-2.12072400	-2.05720500	1.11734200
C	-2.36796100	2.38047200	0.07456300
H	-3.15775200	2.78792500	-0.53982700
C	-3.28704700	0.16604400	-0.52347800
H	-3.45078800	-0.84573700	-0.15567100
C	-0.47605500	2.68290300	1.52434300
H	0.22195100	3.32842400	2.03961400
C	-1.47777700	3.21900900	0.72372500
H	-1.56997900	4.29107700	0.61175300
C	-0.81214000	-1.89454000	-1.46324000
H	-0.15636700	-2.42734300	-2.14841100
H	-1.14771600	-0.97182700	-1.94033100
H	-1.67192200	-2.51761500	-1.24054200
Cl	5.28617800	0.90801400	-0.73272700
N	-3.92669400	0.62558400	-1.51644700
C	-4.90223000	-0.23898900	-2.14657300
H	-5.88065400	0.24634600	-2.11942700
H	-4.64048700	-0.36431500	-3.19986800
H	-4.97912300	-1.22668900	-1.67643600

2d(OFF-2) (acetonitrile)

Imaginary frequency: 0

G = -1699.177116 hartree

C	-2.43908300	0.55710000	1.17285100
C	-1.92441300	1.15063800	-0.07852000
H	-2.85659300	-0.44905000	1.11066500
C	-1.40634400	0.37806800	-1.13131000
C	-1.93193900	2.53661900	-0.23513900
C	-0.91220500	0.96992700	-2.28692000
H	-2.32986500	3.13877600	0.56912100
C	-1.44741600	3.13310300	-1.39061700
H	-0.49920700	0.34649400	-3.06531200
C	-0.93854700	2.35162700	-2.42049300
H	-1.46886300	4.21042900	-1.48710300
H	-0.55715000	2.81149200	-3.32182000
C	0.91944400	-0.97625200	0.34700700
C	1.15251300	0.13046600	1.15652700
C	1.94691300	-1.50155900	-0.43071000
H	0.34750600	0.53159400	1.75591200
C	2.40831400	0.72034900	1.18900900
H	1.75923400	-2.35802600	-1.06193200
C	3.20568800	-0.91677600	-0.40843800
C	3.42161400	0.19001100	0.40181200
H	2.59558800	1.58227000	1.81291500
H	4.00790400	-1.31490900	-1.01280300
S	-1.30065400	-1.39252500	-1.01484300
N	-0.39172500	-1.56507100	0.35258100
O	-2.59591500	-1.96360000	-0.72202000
O	-0.62134900	-1.86898300	-2.19823900
C	-0.58768400	-2.75845400	1.18762500
H	-0.24345200	-2.51633300	2.19118700
H	-0.02790000	-3.61768400	0.81192400
H	-1.64554900	-3.00040100	1.22377100
N	-2.39917100	1.19652800	2.26536100
C	-2.93831000	0.55218000	3.44522400
H	-3.73160700	1.17668400	3.86224600
H	-3.33567000	-0.45114400	3.25107900
H	-2.15533900	0.48534800	4.20393800
Cl	5.00320900	0.93194400	0.43074900

2e(ON) (acetonitrile)

Imaginary frequency: 0

G = -1576.740410 hartree

S	-2.32019100	-1.20017800	0.79297100
N	-1.27790600	-1.93754800	-0.28581500
C	0.06411600	-1.44486200	-0.32352100
C	-2.55708800	0.43711500	0.12845100
C	0.92911400	-1.73420400	0.72987000
H	0.57703900	-2.33121500	1.55665600
C	0.51331900	-0.68285500	-1.40083900
H	-0.15111600	-0.44430000	-2.21771900
C	1.81376500	-0.20541700	-1.41876200
H	2.15247000	0.39575400	-2.25045000

C	2.66884400	-0.48262400	-0.35788200
C	2.22658700	-1.24944400	0.71519100
H	2.89084300	-1.47093600	1.53795900
C	-1.62999800	1.48833100	0.28043100
C	-3.68466500	0.58836400	-0.67921600
H	-4.37773900	-0.23307300	-0.77697700
O	-1.65787900	-1.12930600	2.06797800
O	-3.56712500	-1.91166100	0.69338700
C	-1.89377100	2.67886400	-0.40505200
H	-1.19288900	3.49166800	-0.28230900
C	-0.42421000	1.42835500	1.13654300
H	-0.38633700	0.64113400	1.88630900
C	-3.91669300	1.78275600	-1.34245600
H	-4.79893100	1.89247700	-1.95806100
C	-3.01225000	2.82937700	-1.20667100
H	-3.18420500	3.76693000	-1.71838700
C	-1.88097300	-2.33910800	-1.56802000
H	-1.14423800	-2.93392200	-2.10203800
H	-2.17187200	-1.48733700	-2.18743700
H	-2.75488800	-2.94918100	-1.36759300
N	0.51256500	2.27231000	1.00945900
C	1.63151800	2.18014600	1.92323600
H	2.56107100	2.14041000	1.35359400
H	1.67034700	3.09025300	2.52723800
H	1.58170300	1.31149000	2.58987300
C	4.08492500	0.00813200	-0.39918100
F	4.19498100	1.20528900	-1.01486600
F	4.61834000	0.14916000	0.83220200
F	4.89934100	-0.84227900	-1.07370900

2e(OFF-1) (acetonitrile)

Imaginary frequency: 0

G = -1576.738862 hartree

S	1.50746100	-1.36240000	-1.04482200
N	0.60674400	-1.67846200	0.32353800
C	-0.71465000	-1.12531600	0.35109300
C	1.78232700	0.40019200	-1.00280000
C	-1.71682900	-1.71587900	-0.41494400
H	-1.48448000	-2.58266900	-1.01410400
C	-1.00457300	-0.01029200	1.13422300
H	-0.23211800	0.46012300	1.72432700
C	-2.28873700	0.51289200	1.15014100
H	-2.50662500	1.38144300	1.75476900
C	-3.28268100	-0.06951100	0.37203000

C	-2.99644500	-1.18583400	-0.40937200
H	-3.77005000	-1.64458800	-1.00871100
C	2.77756300	1.02543400	-0.22635600
C	0.83626900	1.15701400	-1.69523300
H	0.07894000	0.65284600	-2.27520400
O	0.66956800	-1.64051100	-2.18016900
O	2.74556100	-2.08227100	-0.89763500
C	2.78221900	2.42413200	-0.19060400
H	3.55296500	2.90816500	0.39146400
C	3.82436300	0.30313100	0.52939500
H	4.03666200	-0.72120400	0.22818400
C	0.86838800	2.54077200	-1.63852000
H	0.13461700	3.11715600	-2.18504800
C	1.84525200	3.17479400	-0.87955800
H	1.88150600	4.25495100	-0.83173500
C	1.33798800	-1.72952400	1.59892500
H	0.67386500	-2.17228700	2.33745800
H	1.65595100	-0.74635200	1.95146000
H	2.20865100	-2.36536800	1.47910700
N	4.44896000	0.86114600	1.48064500
C	5.47635000	0.09381600	2.15280600
H	6.42670300	0.62765300	2.07905600
H	5.23338200	0.02611200	3.21579900
H	5.60135500	-0.91771300	1.74843300
C	-4.68176200	0.46902500	0.41230100
F	-4.72272000	1.77905200	0.73170200
F	-5.31927700	0.33077400	-0.76977600
F	-5.44599100	-0.17265400	1.33295100

2e(OFF-2) (acetonitrile)

Imaginary frequency: 0

G = -1576.739678 hartree

C	-2.78223900	0.81458200	1.18312800
C	-2.28867700	1.23792600	-0.14352800
H	-3.29519200	-0.14691400	1.23908100
C	-1.90150000	0.32530500	-1.13903500
C	-2.18514700	2.59775900	-0.43577000
C	-1.42671700	0.75771700	-2.37097100
H	-2.48182000	3.30664800	0.32402300
C	-1.71962100	3.03572400	-1.66758100
H	-1.11495600	0.02871900	-3.10344500
C	-1.34195200	2.11734300	-2.63928500
H	-1.65329300	4.09656400	-1.86911800
H	-0.97611300	2.45330900	-3.59982700

C	0.37556700	-1.06780600	0.37873600
C	0.73966500	0.05034200	1.12196000
C	1.30864500	-1.70804500	-0.43127900
H	0.00255700	0.54173600	1.74078400
C	2.03738400	0.53391300	1.05423200
H	1.01460000	-2.57192300	-1.00874700
C	2.60556600	-1.22165600	-0.50515800
C	2.96662800	-0.10039300	0.23560000
H	2.31857400	1.40673200	1.62569700
H	3.32975200	-1.71214300	-1.13992400
S	-1.93679900	-1.42800300	-0.84913400
N	-0.97435000	-1.54490300	0.48579400
O	-3.25565400	-1.85402500	-0.44019000
O	-1.36177900	-2.07374900	-2.00659800
C	-1.24159500	-2.59230400	1.48091800
H	-0.84706000	-2.25016300	2.43576200
H	-0.76761900	-3.53859600	1.21216200
H	-2.31415700	-2.73400800	1.57049700
N	-2.62005100	1.54384300	2.20572800
C	-3.14791100	1.06513900	3.46676800
H	-3.85303300	1.80036700	3.86097200
H	-3.64867500	0.09284300	3.38858600
H	-2.33175500	0.98934700	4.18877400
C	4.38250600	0.39597700	0.18736600
F	5.16793500	-0.23264000	1.09829100
F	4.95855400	0.19116600	-1.01563600
F	4.47613600	1.71547500	0.45044400

2f(ON) (acetonitrile)

Imaginary frequency: 0

G = -1444.138590 hartree

S	-2.03471000	-1.20856500	0.79415900
N	-1.01823400	-1.90530100	-0.34502700
C	0.31968600	-1.42381600	-0.40049000
C	-2.29106600	0.44405500	0.18110700
C	1.19214800	-1.69157200	0.65794500
H	0.84555200	-2.27159700	1.49803200
C	0.76779300	-0.69568800	-1.50512000
H	0.10034100	-0.47708100	-2.32445800
C	2.06677500	-0.22288600	-1.54686400
H	2.41986800	0.35451600	-2.38664000
C	2.90938800	-0.48206200	-0.47545100
C	2.48809900	-1.21369800	0.62759200
H	3.17068000	-1.41098400	1.43897400
C	-1.35258700	1.48580700	0.32729200
C	-3.44286400	0.61872600	-0.58596100
H	-4.14288600	-0.19710300	-0.68126400
O	-1.33467500	-1.17501800	2.04946600
O	-3.27838200	-1.92472900	0.70467600
C	-1.62929700	2.69219500	-0.32382300
H	-0.92066100	3.49882800	-0.20458700
C	-0.12544200	1.40497000	1.15094900
H	-0.09768000	0.64167300	1.92609900
C	-3.68804200	1.82944100	-1.21442100
H	-4.58902000	1.95831200	-1.79826500
C	-2.77216700	2.86665000	-1.08597600
H	-2.95396800	3.81619500	-1.57141300
C	-1.66389300	-2.25865500	-1.62108500
H	-0.94912900	-2.83746100	-2.20033200
H	-1.97220700	-1.38395100	-2.19838700
H	-2.53276300	-2.87258500	-1.41158900
N	0.83715300	2.20945800	0.97213100
C	1.97187200	2.11260100	1.86603200
H	2.89003000	2.03731200	1.28146000
H	2.04398200	3.03671000	2.44512800
H	1.91404500	1.26372300	2.55707500
N	4.27645400	0.03251900	-0.50650500
O	4.97518200	-0.12546900	0.48747700
O	4.65776900	0.60364000	-1.52048500

2f(OFF-1) (acetonitrile)

Imaginary frequency: 0

G = -1444.136638 hartree

S	1.15797000	-1.40894600	-0.97668200
N	0.26676900	-1.62376800	0.42633100
C	-1.03526800	-1.04402700	0.45066000
C	1.46708100	0.34637800	-1.02598300
C	-2.04354800	-1.58094200	-0.35304600
H	-1.82724200	-2.43197200	-0.97858000
C	-1.31207800	0.04431400	1.28091500
H	-0.53809700	0.47044700	1.90067600
C	-2.57980100	0.59826100	1.30530100
H	-2.80321900	1.44397000	1.93641100
C	-3.56240700	0.05896500	0.48730500
C	-3.30943000	-1.02588000	-0.34324900
H	-4.09793400	-1.43329400	-0.95624400
C	2.49375600	0.98802500	-0.30610700
C	0.51595500	1.08663900	-1.72984400

H	-0.26409300	0.57041400	-2.26763100
O	0.30005900	-1.73043600	-2.08423500
O	2.37820400	-2.14948900	-0.79851100
C	2.52288600	2.38650000	-0.33667100
H	3.31720400	2.88286200	0.20165900
C	3.55057200	0.28362600	0.45287500
H	3.74624100	-0.75368800	0.18703000
C	0.57361100	2.47060800	-1.73931100
H	-0.16353800	3.03421000	-2.29445800
C	1.58082900	3.12107500	-1.03593000
H	1.63689400	4.20140100	-1.04016600
C	1.02976500	-1.62272700	1.68485900
H	0.36990700	-1.98880800	2.46761600
H	1.39816900	-0.63330700	1.96226100
H	1.86936000	-2.30161800	1.58328500
N	4.20222300	0.87208600	1.36669500
C	5.23855800	0.12191800	2.04447100
H	6.19147500	0.64312700	1.92674500
H	5.02062400	0.09931200	3.11475200
H	5.34465700	-0.90611100	1.67817200
N	-4.90203600	0.64500900	0.50438800
O	-5.74815600	0.17504500	-0.24593300
O	-5.11504400	1.57944300	1.26692900

2f(OFF-2) (acetonitrile)

Imaginary frequency: 0

G = -1444.136703 hartree

C	-1.83926800	-1.01072400	1.47587800
C	-1.66356200	0.44565900	1.30996800
H	-2.52497900	-1.50778400	0.78537300
C	-1.74912500	1.08713400	0.06447000
C	-1.40689000	1.23727800	2.42946700
C	-1.59752400	2.46370700	-0.05414600
H	-1.33670400	0.75117900	3.39202100
C	-1.25816400	2.61212900	2.31888500
H	-1.64757900	2.92309000	-1.02962700
C	-1.36157900	3.22980400	1.07836000
H	-1.06515600	3.20309500	3.20411400
H	-1.24859400	4.30118700	0.98704100
C	0.51232500	-0.67210400	-1.03748700
C	1.31055000	-1.64062400	-0.41087600
C	1.05046100	0.60025200	-1.28357500
H	0.91799200	-2.62370400	-0.20573300
C	2.60216700	-1.34296100	-0.02498700
H	0.47239100	1.34404200	-1.80623300
C	2.33732100	0.90279600	-0.88721500
C	3.10549100	-0.06935900	-0.25817100
H	3.21231400	-2.08389100	0.46741800
H	2.75326500	1.87962000	-1.07828200
S	-2.00378500	0.16949000	-1.43689500
N	-0.80517600	-0.99805200	-1.38939100
O	-3.25284500	-0.54787400	-1.38465800
O	-1.78053000	1.06305700	-2.54451400
C	-1.17852200	-2.40081600	-1.61334700
H	-1.28511100	-2.94888200	-0.67754100
H	-0.40724800	-2.86633900	-2.22307800
H	-2.11974400	-2.43741400	-2.14839500
N	4.46189500	0.24997000	0.15749000
O	5.13482800	-0.63452900	0.67696200
O	4.87387200	1.39049100	-0.02828900
N	-1.24059900	-1.64448600	2.39322600
C	-1.49037500	-3.06549800	2.51479200
H	-1.85597900	-3.27686900	3.52186500
H	-2.21308200	-3.44732500	1.78401200
H	-0.54650300	-3.60307200	2.39998300

1h-S_0 (acetonitrile)

E= -1697.94314720173 hartree

S	3.37383500	-1.29547200	-0.48417800
O	-0.54687400	0.52829300	1.43889600
O	2.63901900	-1.79549200	-1.61766600
O	4.68280100	-1.82649700	-0.20795500
N	2.45837600	-1.55936500	0.87358100
O	1.53454900	0.85739500	2.11211600
C	1.10897300	-1.11019100	0.83861300
C	-1.50467800	-0.15159100	0.74159200
C	3.49897700	0.47788600	-0.61155200
C	0.77490100	0.13514600	1.50359100
C	0.14915200	-1.82003900	0.17743200
H	0.42410200	-2.74633000	-0.30895600
C	-1.17837700	-1.35545800	0.09280700
C	-2.77038600	0.38713800	0.72276700
H	-2.93881100	1.30422100	1.26262300
C	2.44849600	1.29617500	-1.07066700
N	-5.06254500	0.24106400	-0.02016900
O	0.19105200	1.50160800	-1.77192000
C	4.65647000	1.04230100	-0.08621000
H	5.44618800	0.39799900	0.26932800

C	3.16001900	-1.61423500	2.16866900
H	3.96349200	-2.34041600	2.10326100
H	2.43697700	-1.95067000	2.90783400
H	3.55055800	-0.64359100	2.47089400
C	-3.80669800	-0.27036600	0.02339300
C	-2.21673900	-2.00796400	-0.59987300
H	-1.99692100	-2.93297900	-1.11736500
C	2.60380100	2.68054800	-0.97451700
H	1.79435200	3.30561300	-1.32443200
C	-3.48378200	-1.49262400	-0.64005600
H	-4.23875000	-2.01911700	-1.20029200
C	4.79181800	2.42258800	-0.02177900
H	5.70122700	2.85172800	0.37622400
C	-5.38787700	1.53505300	0.57361900
H	-4.55611200	2.22241700	0.42147400
H	-6.22639800	1.94521700	0.01075700
C	1.17779300	0.80054700	-1.66584300
H	1.17302100	-0.23158900	-2.02780200
C	3.76044800	3.24349700	-0.46109500
H	3.85990800	4.31904400	-0.40711900
C	-5.75113000	1.44164300	2.05264000
H	-6.00032200	2.43033000	2.44136100
H	-4.92270500	1.04190400	2.63818300
H	-6.61463400	0.79057200	2.19732900
C	-6.16849600	-0.46444200	-0.66350200
H	-6.06849200	-1.53477500	-0.48833800
H	-7.08224000	-0.15938600	-0.15330500
C	-6.28217900	-0.16634100	-2.15528200
H	-6.42513600	0.90187000	-2.32552300
H	-7.13552900	-0.69685500	-2.58089300
H	-5.38337900	-0.47761700	-2.68852000

2h-S_0 (acetonitrile)

E= -1717.38986964673 hartree

S	3.41728200	-1.17615300	-0.66356900
O	-0.46919600	0.16083800	1.64976000
O	2.67690200	-1.49844200	-1.85693800
O	4.75113400	-1.69954700	-0.51260800
N	2.54787500	-1.73366700	0.63820900
O	1.62156400	0.39028300	2.33148500
C	1.19075000	-1.31591200	0.72515000
C	-1.43057000	-0.38404500	0.84762400
C	3.47823600	0.59346300	-0.46251800
C	0.85775000	-0.21737400	1.61324600
C	0.22675500	-1.90427300	-0.03613100
H	0.50104000	-2.71900300	-0.69265900
C	-1.10772700	-1.44601100	-0.01294100
C	-2.69587900	0.15138300	0.93084500
H	-2.86020600	0.95666300	1.62755000
C	2.41708000	1.45692300	-0.79970700
N	-4.98578900	0.16132200	0.16617200
C	4.61383100	1.07460300	0.18779200
H	5.41001800	0.38921000	0.43534800
C	3.28763600	-2.01160000	1.88159400
H	4.10322200	-2.69273900	1.66201500
H	2.59289500	-2.49784500	2.56246000
H	3.66794400	-1.10578800	2.35176200
C	-3.73341600	-0.36041200	0.12094200
C	-2.14840100	-1.95947400	-0.80912300
H	-1.93089800	-2.77186600	-1.49065500
C	2.55320300	2.80560600	-0.45472000
H	1.74626300	3.47386900	-0.71821400
C	-3.41444400	-1.44159300	-0.75408600
H	-4.17029000	-1.85389800	-1.40193100
C	4.71935700	2.41788200	0.51068500
H	5.60822300	2.78308100	1.00677600
C	-5.30352700	1.32742300	0.98545500
H	-4.46506000	2.02328400	0.96485100
H	-6.13561700	1.84166900	0.50455900
C	1.18810000	1.04073600	-1.51195900
H	1.23499100	0.11321800	-2.07878900
C	3.67944100	3.28390300	0.19258300
H	3.75148300	4.33464500	0.44006100
C	-5.67524100	0.96805700	2.42099700
H	-5.91682200	1.87091700	2.98423400
H	-4.85368800	0.45876500	2.92562900
H	-6.54577000	0.31049600	2.44028500
C	-6.08913800	-0.39793500	-0.61049100
H	-6.00391800	-1.48358200	-0.63487500
H	-7.00727900	-0.17961000	-0.06498100
C	-6.17571100	0.17177400	-2.02320000
H	-6.30837000	1.25445000	-1.99515500
H	-7.02517600	-0.26231100	-2.55310600
H	-5.26992300	-0.04434100	-2.59063600
N	0.13613800	1.74820500	-1.47784200
C	-1.00175300	1.30330300	-2.25551300
H	-0.83110900	0.35343000	-2.77498100
H	-1.86780900	1.19847100	-1.59992300

H	-1.24945500	2.07262700	-2.99103900

1h_without_aldehyde-S_0 (acetonitrile)

E= -1584.57038230765 hartree

S	3.39873900	-1.28264000	-0.45225900
O	-0.50340500	0.80690500	1.16812500
O	2.60508200	-1.81591500	-1.53039700
O	4.67267400	-1.87978900	-0.13716800
N	2.48019200	-1.38149700	0.92869800
O	1.58348200	1.21923700	1.77395300
C	1.13437500	-0.92596200	0.83698000
C	-1.47537800	0.02570200	0.60987400
C	3.63324300	0.44229700	-0.75585200
C	0.81690300	0.41470700	1.29001100
C	0.16567800	-1.72255800	0.30793000
H	0.42978900	-2.71758900	-0.02447900
C	-1.16494600	-1.27061500	0.16719800
C	-2.74032300	0.56272800	0.51998300
H	-2.89641300	1.55785500	0.90278300
C	2.69294900	1.12964700	-1.51821000
N	-5.04778700	0.30915500	-0.14640200
C	4.70352600	1.10135200	-0.15863700
H	5.42673100	0.54638200	0.42108500
C	3.17384700	-1.27578100	2.22252400
H	4.01923900	-1.95606900	2.22497900
H	2.46866300	-1.58518200	2.99101700
H	3.50574400	-0.25980300	2.43406100
C	-3.79167500	-0.19665200	-0.03902600
C	-2.21779000	-2.02390000	-0.38500100
H	-2.01135300	-3.02506800	-0.74142200
C	2.82808300	2.50182100	-1.67805600
H	2.10422800	3.04619600	-2.26940100
C	-3.48524500	-1.51511400	-0.48959300
H	-4.25191200	-2.12560900	-0.93769300
C	4.82897700	2.47334700	-0.32847100
H	5.65915800	2.99604600	0.12715200
C	-5.35548000	1.68901600	0.21730500
H	-4.52174000	2.33168100	-0.06456100
H	-6.20020900	2.00308500	-0.39613500
C	3.89120200	3.17263000	-1.08203400
H	3.99251900	4.24226200	-1.20936300
C	-5.69589000	1.85955300	1.69500900
H	-5.93350500	2.90301400	1.90838900
H	-4.86074300	1.56142100	2.32955200
H	-6.56068200	1.25098400	1.96391000
C	-6.16954200	-0.49073500	-0.63096700
H	-6.06638400	-1.51513500	-0.27531000
H	-7.07060700	-0.09749000	-0.15967900
C	-6.32073200	-0.45591900	-2.14869700
H	-6.46743300	0.56717100	-2.49844200
H	-7.18478000	-1.04806100	-2.45481100
H	-5.43582000	-0.85938700	-2.64192400
H	1.87549400	0.59505400	-1.97935200

1h-S_1 (acetonitrile)

E= -1697.92677805726 hartree

S	3.33578800	-0.93285800	-0.79968500
O	-0.29627600	0.14090100	1.83845900
O	2.56741600	-1.61148300	-1.81315800
O	4.76267200	-1.13826700	-0.75472100
N	2.78809500	-1.55675100	0.71435600
O	1.76153200	0.40376600	2.58196600
C	1.43190300	-1.31122300	0.94811200
C	-1.20856200	-0.42761700	1.00796400
C	2.95667100	0.76995000	-0.74623700
C	1.03440100	-0.21111700	1.84941300
C	0.48254200	-1.98992100	0.24265500
H	0.78384600	-2.81250800	-0.38808500
C	-0.85620400	-1.55308400	0.22983000
C	-2.45179300	0.13285200	0.96196900
H	-2.64900600	0.99489000	1.57549200
C	1.67318300	1.33224200	-1.11156800
N	-4.66357800	0.11474100	0.02921300
O	-0.52023600	1.24470700	-2.03629800
C	3.95330300	1.56048100	-0.15826400
H	4.89988500	1.10184300	0.08750600
C	3.75450100	-1.48787100	1.82094600
H	4.60613900	-2.10990700	1.56172600
H	3.27423300	-1.89464000	2.70707000
H	4.08842700	-0.47343200	2.03369600
C	-3.44533300	-0.43346200	0.11640700
C	-1.85737300	-2.13104400	-0.58682900
H	-1.60343500	-2.99346100	-1.18669200
C	1.51229200	2.71384100	-0.78502900
H	0.55876300	3.15539200	-1.03798500
C	-3.10698300	-1.60111100	-0.64935900
H	-3.83539800	-2.05136200	-1.30117200
C	3.74927200	2.89831400	0.11069000

H	4.53429800	3.49085000	0.55776300
C	-5.01732200	1.35617300	0.72979600
H	-4.15938500	2.02263000	0.73173000
H	-5.79030200	1.83735800	0.13424300
C	0.58303600	0.68087800	-1.77106100
H	0.73035600	-0.35834900	-2.06747700
C	2.49531100	3.46606900	-0.20112200
H	2.30978200	4.51040400	0.01810500
C	-5.52436600	1.08972500	2.14349500
H	-5.81121800	2.03484200	2.60354900
H	-4.75432900	0.62418100	2.75802300
H	-6.39613300	0.43573200	2.12871600
C	-5.74100300	-0.47146900	-0.77949500
H	-5.67958400	-1.55451900	-0.73112100
H	-6.67517000	-0.18619300	-0.29992800
C	-5.70910700	0.02968400	-2.21976100
H	-5.79260700	1.11571500	-2.25684300
H	-6.54941100	-0.39850500	-2.76550500
H	-4.78506300	-0.26190000	-2.71813200

2h-S_1 (acetonitrile)

E= -1717.36388444763 hartree

S	3.02004600	-1.56296200	-0.31463100
O	-1.10591300	0.71565800	1.57495000
O	2.06143500	-1.75548500	-1.37718100
O	3.75848600	-2.71636100	0.14076000
N	2.07503600	-1.02573600	1.06497100
O	0.88128900	1.18408700	2.38639900
C	0.73481400	-0.77048700	1.00904500
C	-1.95663400	-0.00035800	0.79083800
C	4.05260100	-0.21942100	-0.55977000
C	0.22757800	0.42755800	1.71740600
C	-0.15273800	-1.53869800	0.27732600
H	0.19540000	-2.43431100	-0.20920800
C	-1.49066100	-1.16594900	0.13854900
C	-3.24467500	0.44094400	0.69231300
H	-3.52269100	1.33145100	1.23012800
C	3.50974800	1.10028700	-0.81487900
N	-5.45148600	0.12367400	-0.21026900
C	5.42742300	-0.41978500	-0.34512300
H	5.77885500	-1.42018300	-0.13797000
C	2.91007400	-0.66676600	2.21694700
H	3.71992700	-1.39063700	2.27175000
H	2.31788400	-0.73191600	3.12302600
H	3.32116600	0.33578000	2.12056300
C	-4.18131400	-0.29074000	-0.08693600
C	-2.43623200	-1.89705800	-0.62732600
H	-2.10769100	-2.79257000	-1.13639300
C	4.46225000	2.15800400	-0.78879000
H	4.08725500	3.15903300	-0.95165900
C	-3.72663300	-1.48538400	-0.74094400
H	-4.40522500	-2.05962400	-1.34848000
C	6.30708100	0.63544300	-0.37930700
H	7.36380300	0.46970100	-0.22479700
C	-5.92378300	1.38130300	0.37840000
H	-5.13799300	2.12925200	0.30437200
H	-6.74512700	1.72906800	-0.24568600
C	2.13094100	1.37267300	-1.06500000
H	1.48419400	0.51316200	-1.24021400
C	5.80057300	1.94511700	-0.59217700
H	6.48208300	2.78658100	-0.59883400
C	-6.39248300	1.20784500	1.81936900
H	-6.76019700	2.16130300	2.19884400
H	-5.57893500	0.87096300	2.46164300
H	-7.20180900	0.47970000	1.87986600
C	-6.46481900	-0.64472700	-0.94205400
H	-6.29480900	-1.70690300	-0.78645300
H	-7.42307200	-0.41270500	-0.48058200
C	-6.49501300	-0.29786300	-2.42653900
H	-6.69074800	0.76451500	-2.57439800
H	-7.28777200	-0.86373800	-2.91615700
H	-5.54772900	-0.54238200	-2.90713900
N	1.61535900	2.57067400	-1.11900700
C	0.19978600	2.64233500	-1.39464800
H	-0.26000300	1.66347300	-1.59929000
H	-0.33570800	3.09174200	-0.54937200
H	0.00616000	3.28754100	-2.25894800

1h_without_aldehyde-S_1 (acetonitrile)

E= -1584.56442927763 hartree

S	3.47746000	-1.24655600	-0.42507300
O	-0.55097000	0.69286600	1.45393800
O	2.73529800	-1.95490000	-1.43480500
O	4.79295700	-1.70839800	-0.05867400
N	2.55421200	-1.25708200	0.99741100
O	1.52274100	1.18259200	2.08137700
C	1.20555400	-0.87686500	0.90787400
C	-1.43855300	-0.03364600	0.70554700

C	3.53298800	0.45711000	-0.84786300
C	0.81392200	0.36492100	1.50832100
C	0.29294700	-1.67314400	0.19148400
H	0.61242100	-2.60445600	-0.24444400
C	-1.04571800	-1.23391900	0.06714900
C	-2.72090700	0.45444500	0.63718000
H	-2.93903800	1.36946200	1.16325200
C	2.40421000	1.03312200	-1.43609600
N	-4.99709300	0.23327500	-0.15383800
C	4.62383700	1.22977600	-0.45690800
H	5.48107400	0.75931900	0.00251700
C	3.31427000	-1.01632200	2.23381700
H	4.08216000	-1.78019700	2.31900100
H	2.62160500	-1.10534200	3.06498800
H	3.76900500	-0.02698800	2.26071100
C	-3.72208500	-0.24689300	-0.08603100
C	-2.05680900	-1.92392800	-0.64822200
H	-1.79312300	-2.84548000	-1.15040500
C	2.38114700	2.40722600	-1.63233600
H	1.51422200	2.86776600	-2.08696900
C	-3.34637600	-1.45696200	-0.72895700
H	-4.06604000	-2.01695700	-1.30335000
C	4.59069900	2.59997800	-0.67346400
H	5.43729000	3.20880900	-0.38626000
C	-5.37728400	1.51524800	0.42823000
H	-4.56435200	2.22777800	0.30002100
H	-6.21876900	1.89180100	-0.15282700
C	3.46723000	3.18873200	-1.25093600
H	3.44121600	4.25893700	-1.40773100
C	-5.77345900	1.40082600	1.90079900
H	-6.07142500	2.38099300	2.27538400
H	-4.94114300	1.03728500	2.50321300
H	-6.61279100	0.71578600	2.02413800
C	-6.06493400	-0.50701000	-0.81551100
H	-5.92822900	-1.57273500	-0.64121400
H	-6.99819000	-0.22575000	-0.32831400
C	-6.14929500	-0.20600400	-2.31222900
H	-6.32554100	0.85629000	-2.48396200
H	-6.97527300	-0.76701000	-2.75126000
H	-5.22782200	-0.48921100	-2.82061600
H	1.57504500	0.41401800	-1.74548100

9. References

S1. Frisch, M. J.; Trucks, G. W.; Schlegel, H. B.; Scuseria, G. E.; Robb, M. A.; Cheeseman, J. R.; Scalmani, G.; Barone, V.; Mennucci, B.; Petersson, G. A.; Nakatsuji, H.; Caricato, M.; Li, X.; Hratchian, H. P.; Izmaylov, A. F.; Bloino, J.; Zheng, G.; Sonnenberg, J. L.; Hada, M.; Ehara, M.; Toyota, K.; Fukuda, R.; Hasegawa, J.; Ishida, M.; Nakajima, T.; Honda, Y.; Kitao, O.; Nakai, H.; Vreven, T.; Montgomery, J. A., Jr.; Peralta, J. E.; Ogliaro, F.; Bearpark, M.; Heyd, J. J.; Brothers, E.; Kudin, K. N.; Staroverov, V. N.; Kobayashi, R.; Normand, J.; Raghavachari, K.; Rendell, A.; Burant, J. C.; Iyengar, S. S.; Tomasi, J.; Cossi, M.; Rega, N.; Millam, J. M.; Klene, M.; Knox, J. E.; Cross, J. B.; Bakken, V.; Adamo, C.; Jaramillo, J.; Gomperts, R.; Stratmann, R. E.; Yazyev, O.; Austin, A. J.; Cammi, R.; Pomelli, C.; Ochterski, J. W.; Martin, R. L.; Morokuma, K.; Zakrzewski, V. G.; Voth, G. A.; Salvador, P.; Dannenberg, J. J.; Dapprich, S.; Daniels, A. D.; Farkas, Ö.; Foresman, J. B.; Ortiz, J. V.; Cioslowski, J.; Fox, D. J. Gaussian 09, revision D.01; Gaussian, Inc.: Wallingford, CT, **2010**.

S2. Z. Tang, Y. Song, S. Zhang, W. Wang, Y. Xu, D. Wu, W. Wu, P. Su, *J. Comput. Chem*. **2021**, *42*, 2341-2351.

S3. E. D. Glendening, C. R. Landis, F. Weinhold, *Wiley Interdiscip. Rev.: Comput. Mol. Sci.* **2012**, *2*, 1-42.

S4. R. F. Bader, *Acc. Chem. Res.* **1985**, *18*, 9-15.

S5. E. R. Johnson, S. Keinan, P. Mori-Sánchez, J. A. Contreras-García, J. Cohen, W. Yang, *J. Am. Chem. Soc.* **2010**, *132*, 6498-6506.

S6. T. Lu, F. Chen, *J. Comput. Chem.* **2012**, *33*, 580-592.

S7. W. Humphrey, A. Dalke, K. Schulten, *J. Mol. Graphics* **1996**, *14*, 33-38.

S8. K. G. Rajeev, S. M. Shashidhar, K. Pius, V. M. Bhatt, *Tetrahedron* **1994**, *50*, 5425-5438.

S9. Y. Hai, H. Zou, H. Ye, L. You, *J. Org. Chem.* **2018**, *83*, 9858-9869.

S10. H. Zou, Y. Hai, H. Ye, L. You, *J. Am. Chem. Soc.* **2019**, *141*, 16344-16353.

S11. C. Reichardt, T. Welton, *Solvents and Solvent Effects in Organic Chemistry.* WILEY-VCH Verlag GmbH & Co. KGaA, Weinheim, **2011**.
